# Supplementary material for: Epstein-Barr virus orchestrates spatial reorganization and immunomodulation in the classic Hodgkin lymphoma tumor microenvironment
Source: Cell Rep Med. 2026 Mar 31;7(4):102722. doi: 10.1016/j.xcrm.2026.102722 (PMC13130692; doi:10.1016/j.xcrm.2026.102722)
Supplement: Document S1. Figures S1–S15 [file mmc1.pdf]

Cell Reports Medicine, Volume 7

Supplemental information

Epstein-Barr virus orchestrates spatial reorganization and immunomodulation in the classic Hodgkin lymphoma tumor microenvironment

Yao Yu Yeo, Huaying Qiu, Yunhao Bai, Bokai Zhu, Yuzhou Chang, Fabio Iannelli, Stephanie Pei Tung Yiu, Jason Yeung, Hendrik A. Michel, Yuchen Wang, Yang Wang, Wenrui Wu, Kyle Wright, Muhammad Shaban, Sam Sadigh, Dingani Nkosi, Vignesh Shanmugam, Philip Rock, Precious Cramer, Julia Paczkowska, Pierre Stephan, Guanrui Liao, Amy Y. Huang, Hongbo Wang, Han Chen, Leonie Frauenfeld, Louisa Kaufmann, Stefano Pileri, Bidisha Mitra, Benjamin E. Gewurz, Bo Zhao, Garry P. Nolan, Baochun Zhang, Alex K. Shalek, Michael Angelo, Christian M. Schürch, Faisal Mahmood, Roberto Chiarle, Qin Ma, W. Richard Burack, Margaret A. Shipp, Scott J. Rodig, and Sizun Jiang

Supplementary Figures

MIBI staining: Antibody Validation

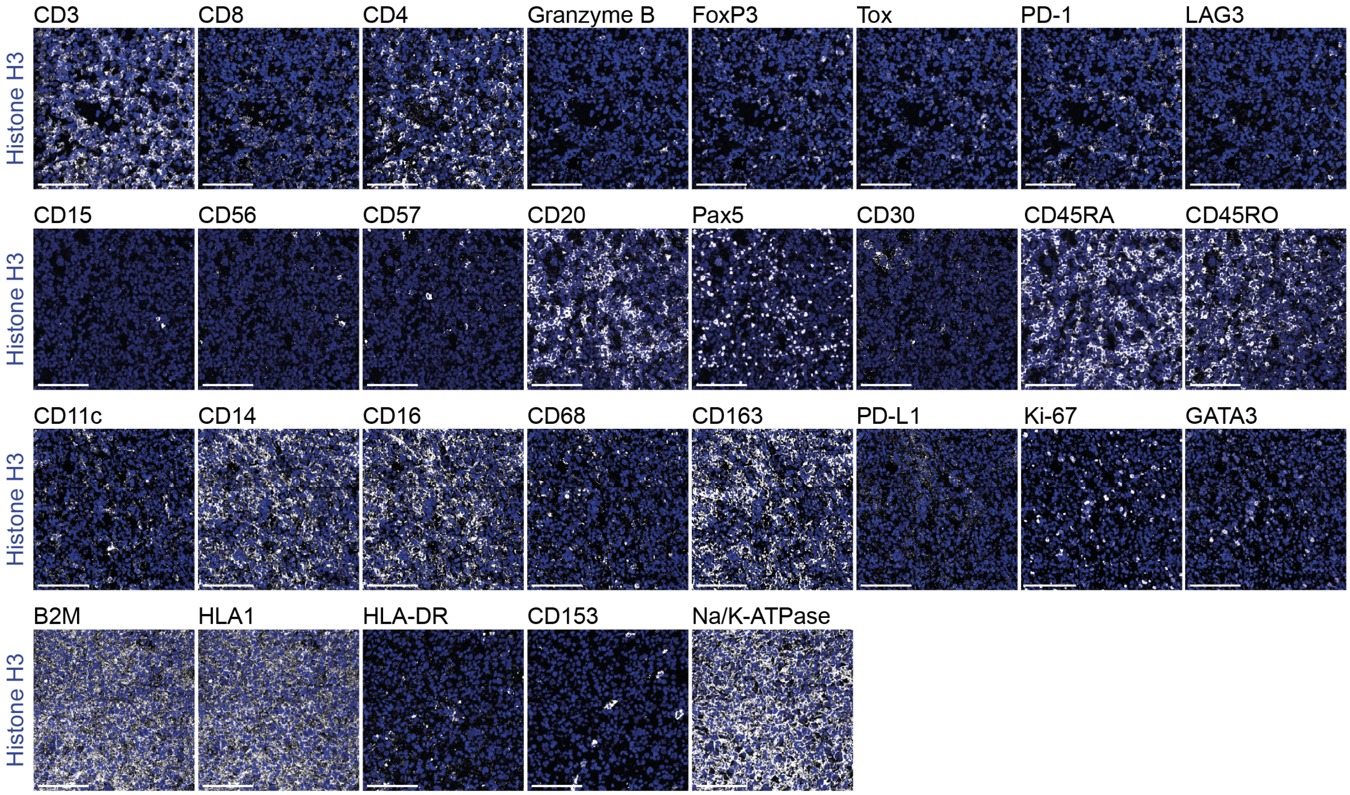

Figure S1, related to Figure 1. Validation of MIBI antibody staining specificity. Representative MIBI images across cHL tissue sections, showing 29 antibody markers (white) overlaid with the cell nucleus antibody marker Histone H3 (blue). Scale bar: 100 µm.

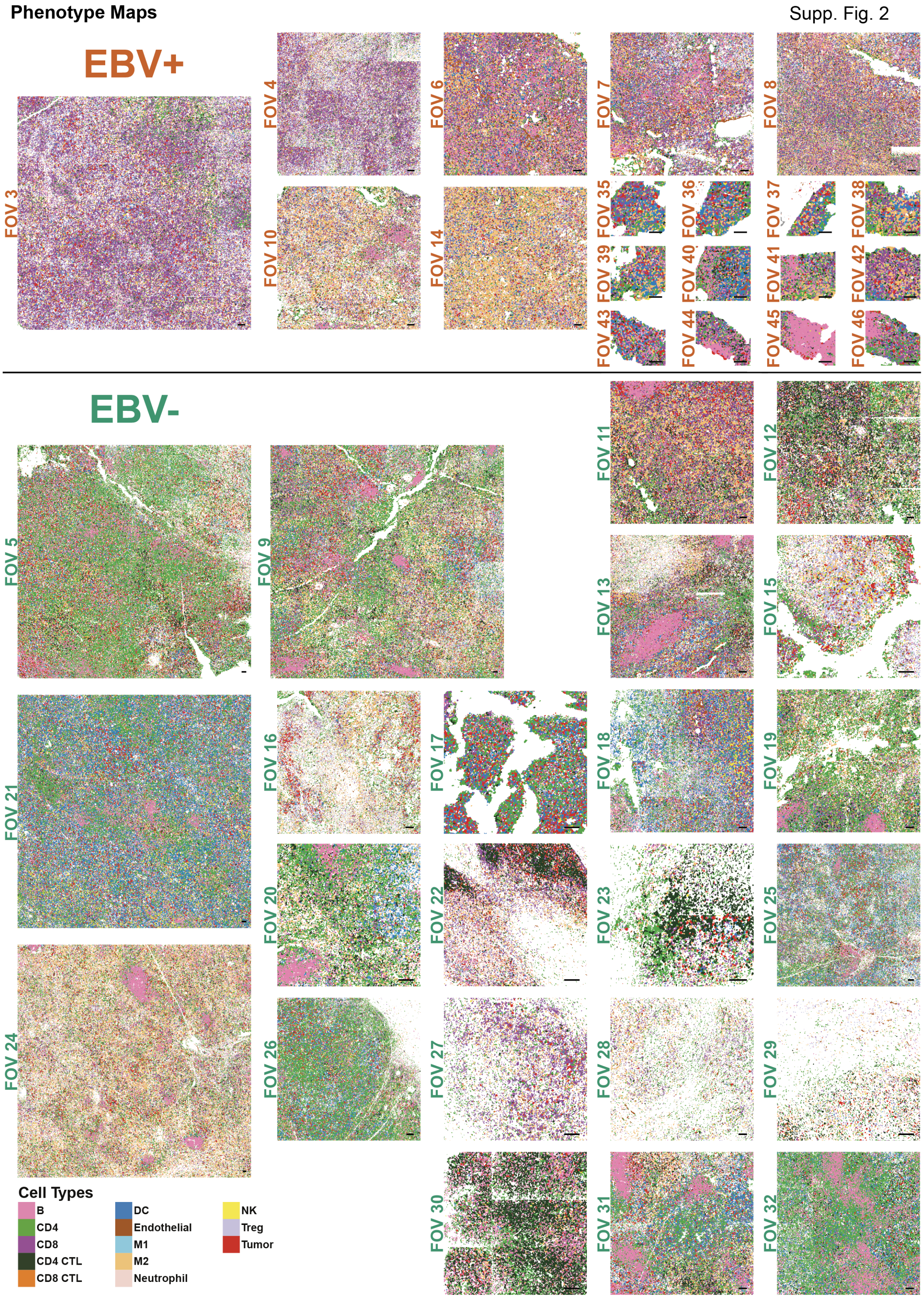

Figure S2, related to Figure 2. Validation of cell phenotyping from MIBI images. Phenotype maps of all the MIBI-acquired FOVs across cHL tissue sections, generated through iterative clustering and annotation based on single-cell MIBI features. Scale bar: 100 µm.

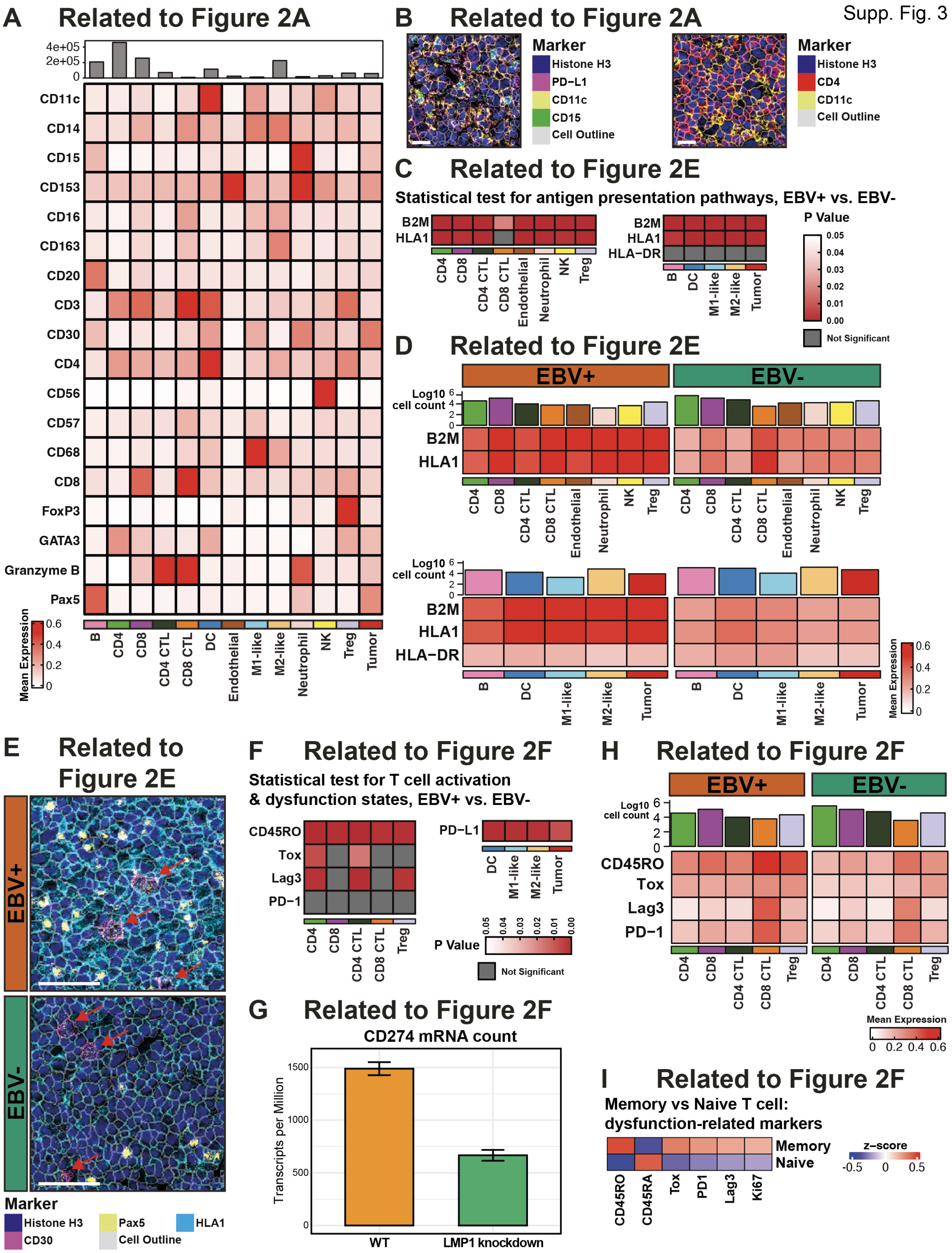

Figure S3, related to Figure 2. Validation and quantification of marker expression on a cell type level. **(A)** Relative mean expression levels of phenotypic markers for the annotated cell phenotypes in the EBV-positive and EBV-negative cHL MIBI dataset. **(B)** Representative MIBI images showing neutrophils (CD15) close to immune constituents that have express PD-L1, such as dendritic cells (CD11c) (left), and dendritic cells (CD11c) in close contact with CD4 T cells (CD4) (right). Cell outlines are shown to indicate segmented cell boundaries. Scale bar: 20 µm. **(C)** and **(F)** Test results for **Figs. 2E** & **2F**, respectively. P values were generated from one-sided Wilcoxon rank sum tests, with the alternative hypothesis that the distribution of a given marker for the EBV+ population is shifted to the right of the distribution for the EBV- population. The test results were adjusted for multiple comparisons using the Benjamini-Hochberg method with a targeted FDR at 0.05. Unadjusted p-value and Benjamini-Hochberg corrected test results are in **Supp Table 4**. **(D)** and **(H)** Relative mean expression level heatmaps, a re-representation of relative z-score expression level heatmaps in **Figs. 2E** & **2F**. **(E)** Representative MIBI images showing MHC Class I (HLA1) expression differences between the EBV-positive and EBV-negative cHL TME, with additional markers for B cells (Pax5^{hi}) and HRS cells (CD30, Pax5^{lo}) shown. Red arrows point to HRS cells. Cell outlines are shown to indicate segmented cell boundaries. Scale bar: 50 µm. **(G)** Relative expression of *CD274* that encodes for PD-L1 upon knockdown of LMP1 in the EBV-positive GM12878 cell line (n=3 each). The data used to generate this barplot is in **Supp Table 9**. **(I)** Expression heatmap of immune exhaustion markers on T cells stratified into memory and naive populations based on the relative expression of CD45RO and CD45RA. The data used to generate this heatmap is in **Supp Table 10**.

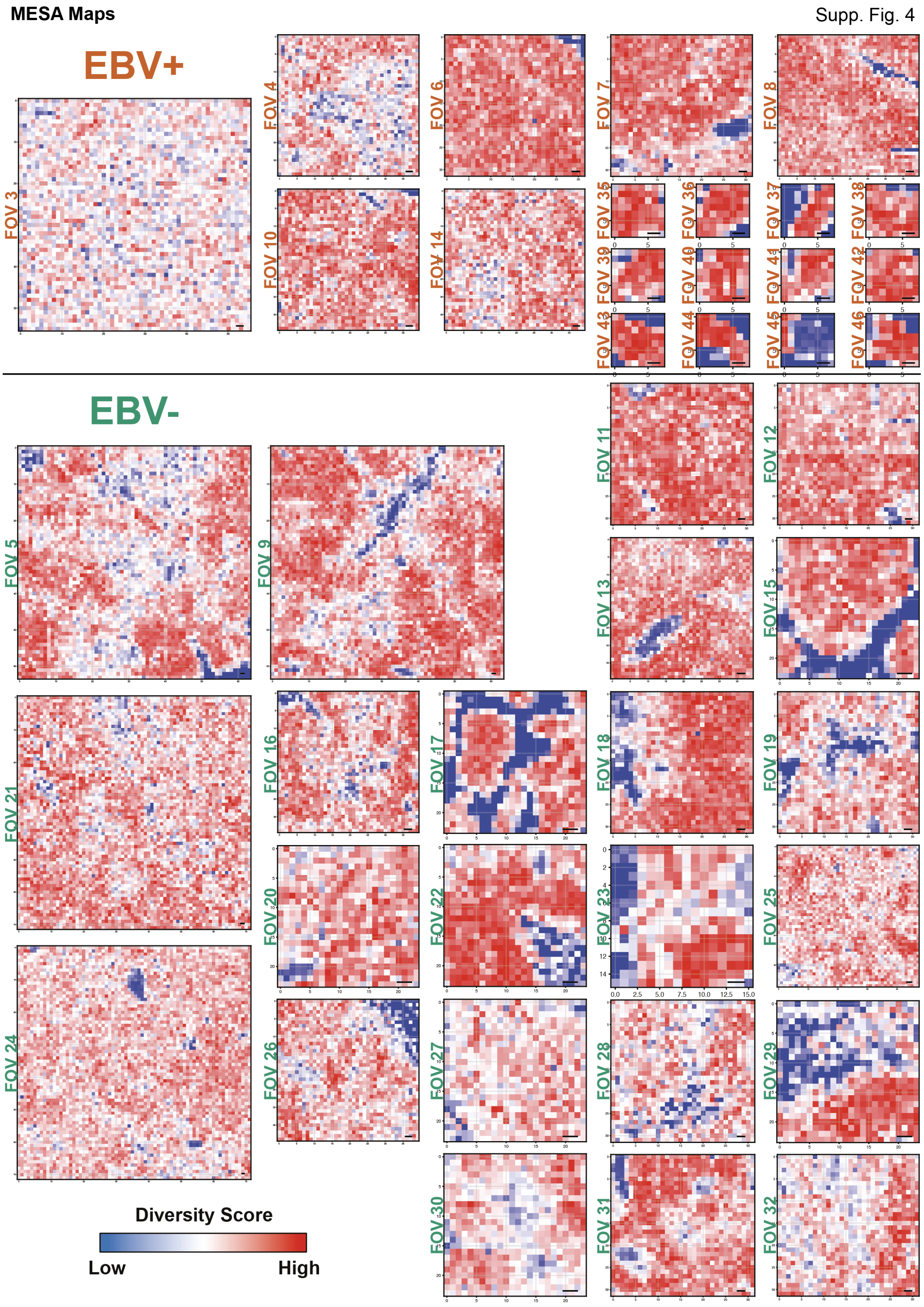

Figure S4, related to Figure 3. Validation of spatial diversity hotpot identification from the annotated cell types. Spatial diversity maps of all MIBI-acquired FOVs across cHL tissue sections, generated through MESA (1) based on annotated cell phenotypes (see **Supp Fig. S2**). Scale bar: 100 µm.

Cell Neighborhood Maps

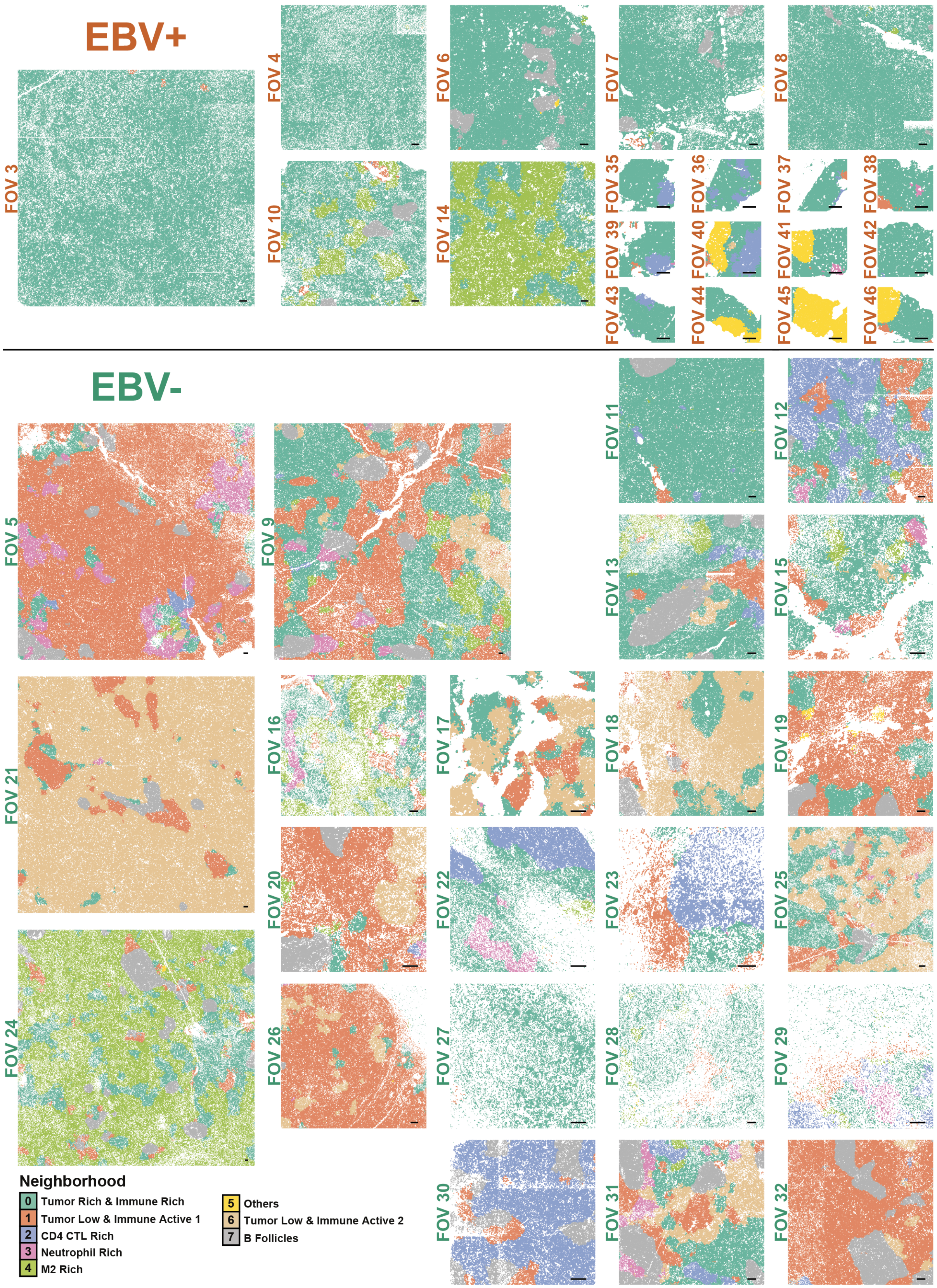

Figure S5, related to Figure 3. Validation of cell neighborhood identification from annotated cell types. Cell neighborhood maps of all MIBI-acquired FOVs across cHL tissue sections, generated through spatial LDA (2) based on annotated cell phenotypes (see **Supp Fig. S2**). Scale bar: 100 µm.

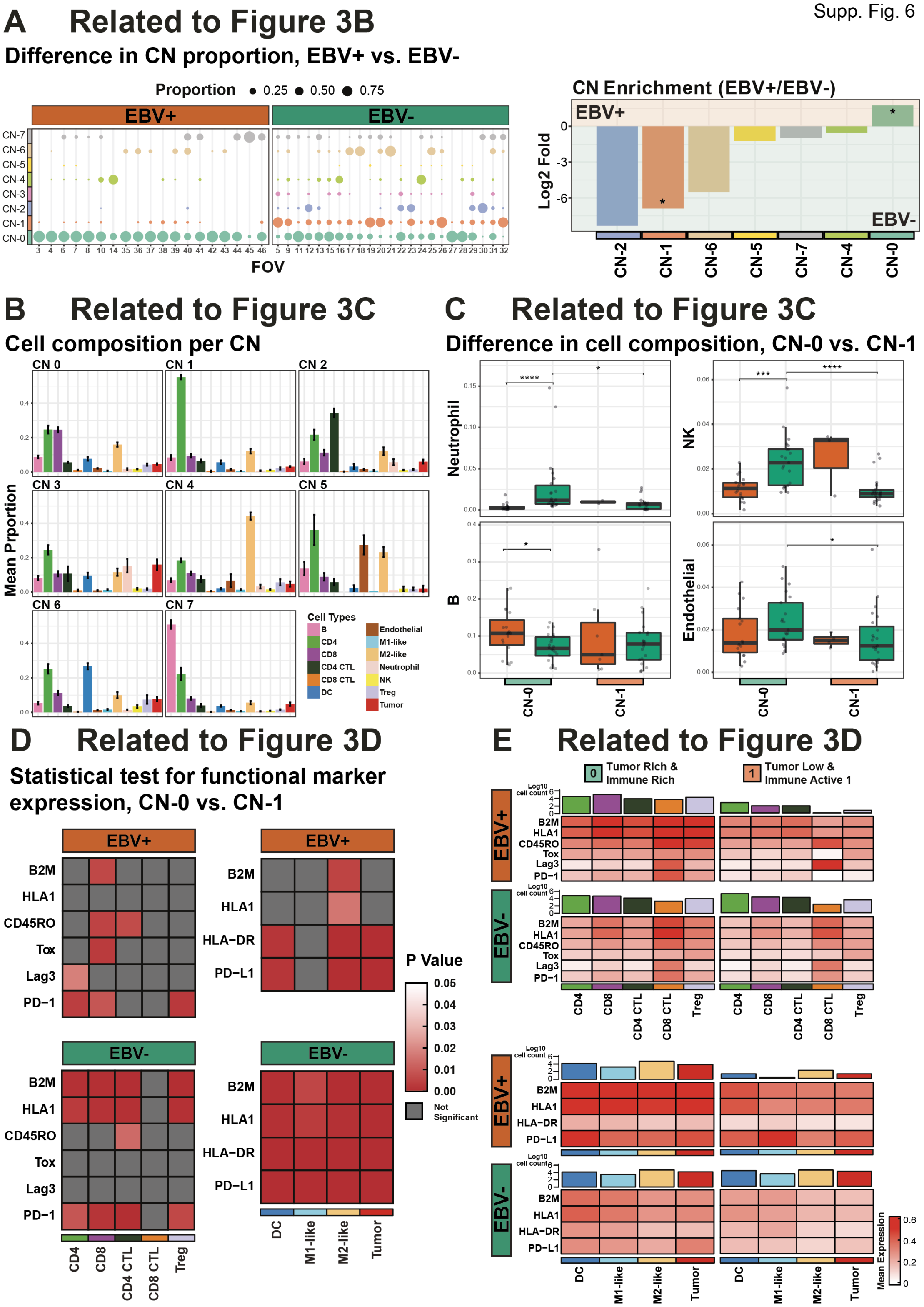

Figure S6, related to Figure 3. Statistical test results for comparison of cell proportions and functional marker expression on a cell neighborhood level. (A) Left: Relative proportion of cells assigned to each CN across EBV-positive and EBV-negative cHL FOVs. Right: Log2 fold enrichment plot of cell proportions assigned to each CN between EBV-positive and EBV-negative cHL FOVs. Note that CN3 is not shown due to its absence from the EBV+ cHL TME. Significance stars (* $p \leq 0.05$) are only shown for statistically significant comparisons ($p \leq 0.05$). Two-sided Wilcoxon rank sum tests were conducted for all cell type proportions within each CN, with alternative hypotheses that the proportions of the given cell type in each CN present in the two strata were not equal. Test results were adjusted for multiple comparisons using the Benjamini-Hochberg method with a targeted false discovery rate (FDR) at 0.05. **(B)** Abundance of each cell type within each CN (mean ± 1 s.e.). **(C)** Relative proportion of other cell types within CN-0 and CN-1, stratified by EBV status. Significance stars are only shown for statistically significant comparisons ($p \leq 0.05$). **(D)** Test results for **Fig. 3E**. P values were generated from two-sided Wilcoxon rank sum tests, with the alternative hypothesis that within the EBV+ or EBV- population respectively, the distribution of a given marker is different across CN-0 and CN-1. The test results were adjusted for multiple comparisons using the Benjamini-Hochberg method with a targeted FDR at 0.05. Unadjusted p-value and Benjamini-Hochberg corrected test results are in **Supp Table 4**. **(E)** Relative mean expression level heatmap, a re-representation of relative z-score expression level heatmap in **Fig. 3E**.

Tumor Score Maps

Supp. Fig. 7

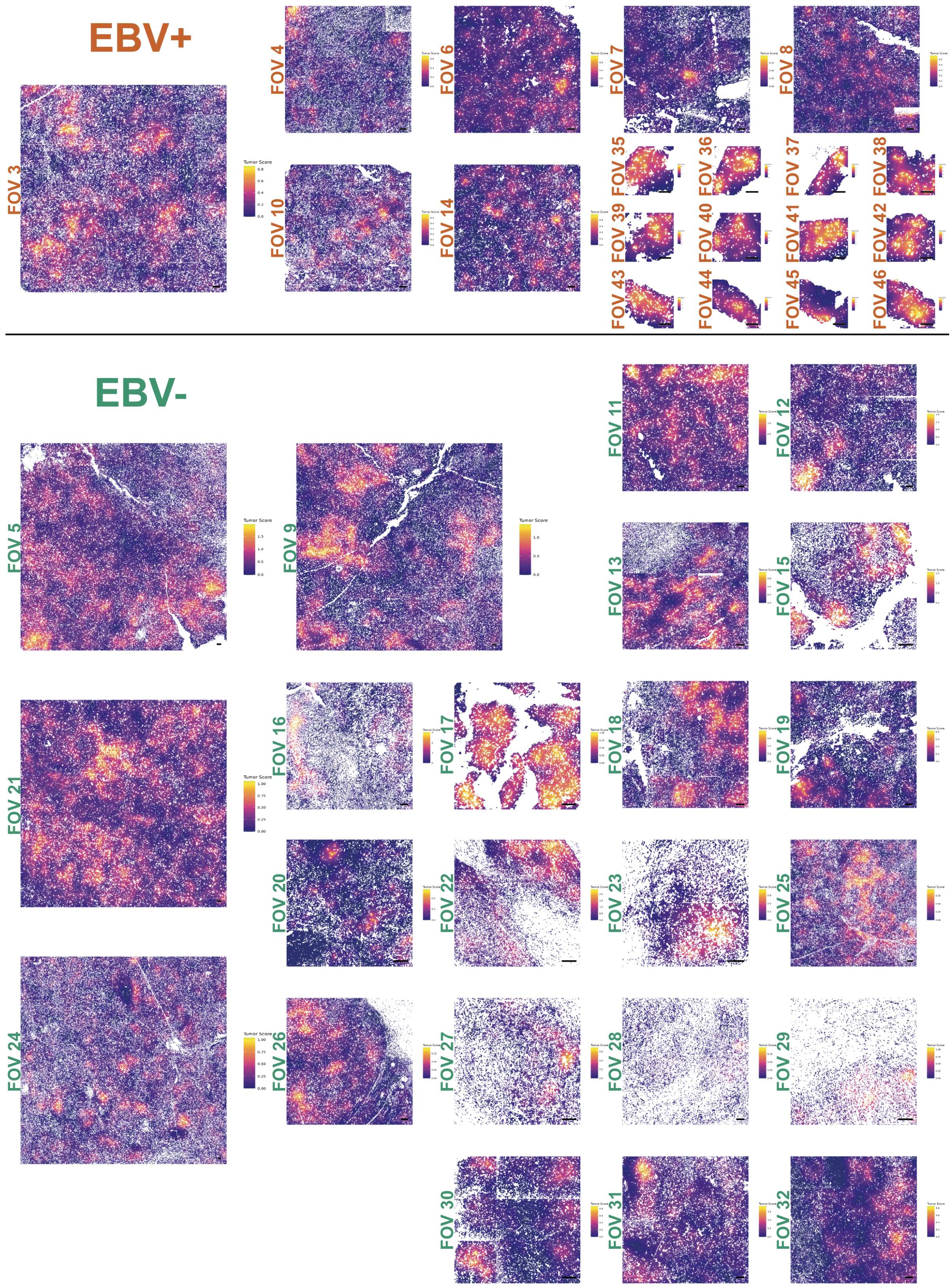

Figure S7, related to Figure 4. Validation of tumor score metric. Tumor score maps of all MIBI-acquired FOVs across cHL tissue sections, generated based on the spatial proximity to tumor cells (see **Materials & Methods**). HRS cells are not considered in the tumor score metric and do not have a color assigned. Scale bar: 100 µm.

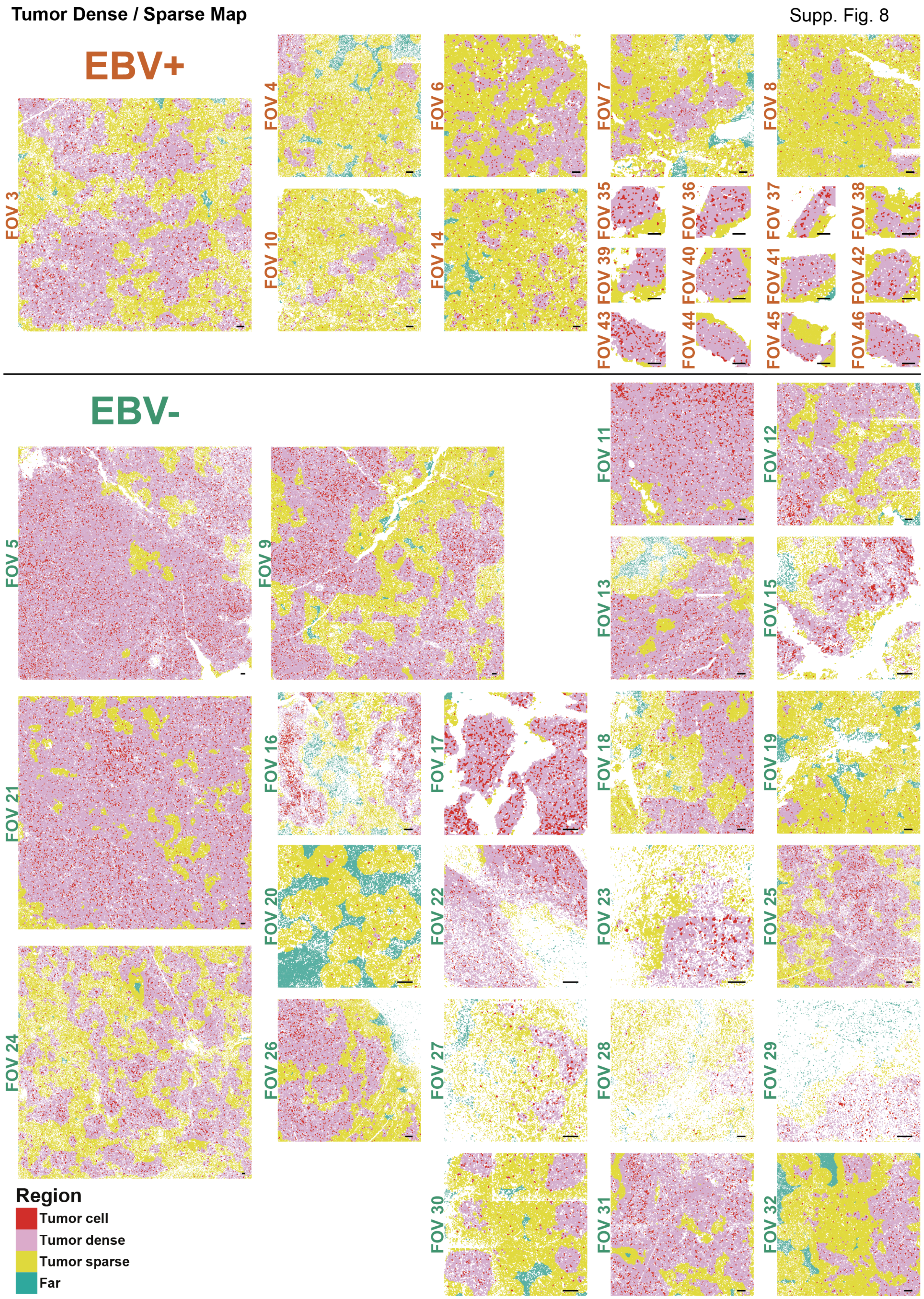

Figure S8, related to Figure 4. Validation of tumor dense/sparse region stratification. Tumor dense and tumor sparse maps of all 34 MIBI-acquired FOVs across cHL tissue sections, generated based on the tumor score metric of each non-tumor cell (see **Materials & Methods**). Scale bar: 100 µm.

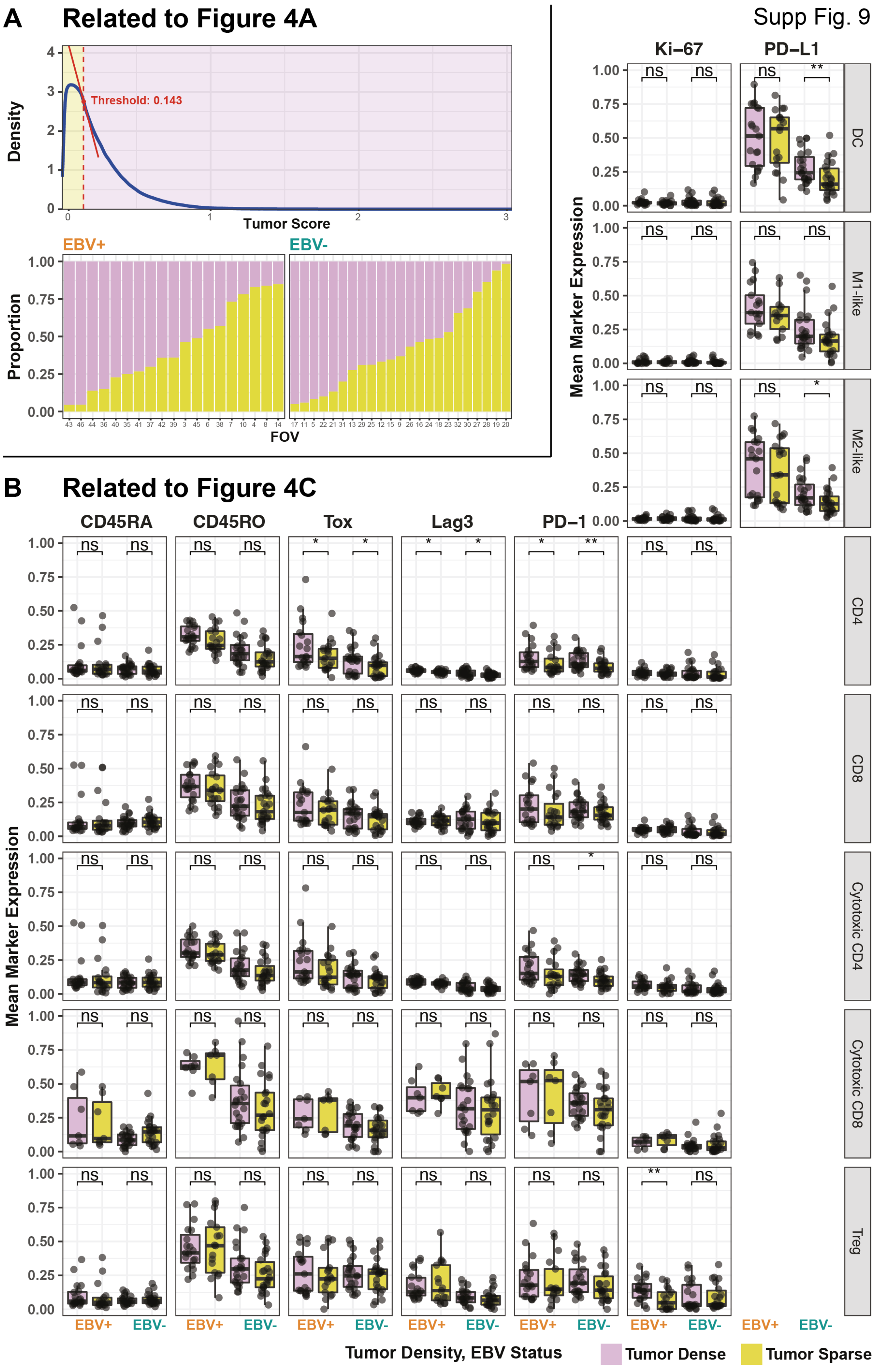

Figure S9, related to Figure 4. Tissue stratification into tumor dense/sparse regions and comparison of the corresponding functional marker expression. (A) Top: Cutoff point for stratifying cells into tumor dense/sparse regions. Bottom: Barplot showing the proportion of cells in each FOV that are assigned to tumor dense/sparse regions, stratified by EBV status. **(B)** Relative expression of markers that comprise the exhaustion score metric. For each tumor dense and tumor sparse pair, a one-sided T test was performed, with the alternative hypothesis that the difference between the mean exhaustion score of the tumor dense region in an FOV and that of the tumor sparse region is greater than 0 (* $p \leq 0.05$, ** $p \leq 0.01$, *** $p \leq 0.001$, **** $p \leq 0.0001$). The test results were adjusted for multiple comparisons using the Benjamini-Hochberg method with a targeted false discovery rate (FDR) at 0.05. Unadjusted p-value and Benjamini-Hochberg corrected test results are in **Supp Table 4**.

Supp Fig. 10

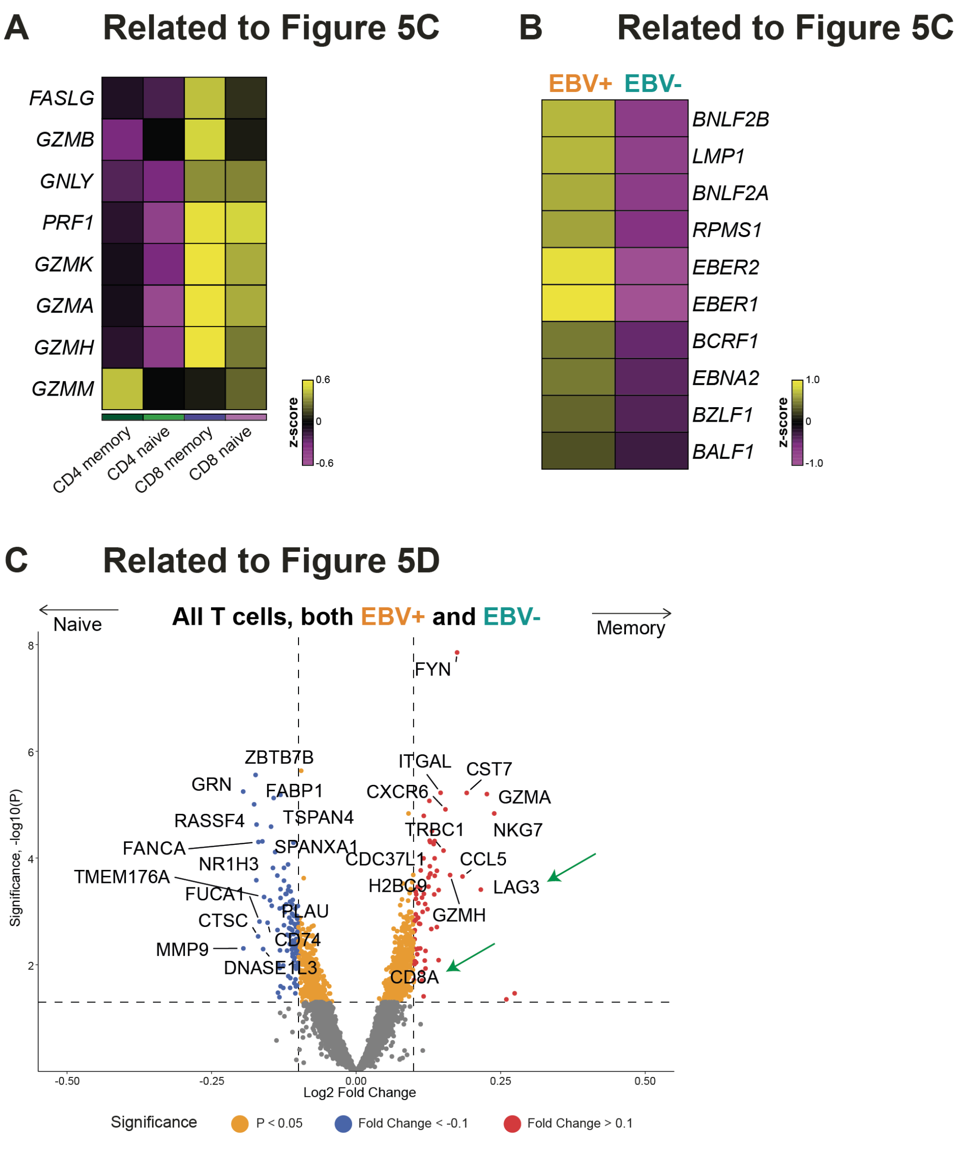

Figure S10, related to Figure 5. Comparison EBV-linked transcriptional differences. (A) Expression heatmap of cytotoxic transcripts associated with each annotated cell region. **(B)** Expression heatmap of EBV genes in EBV-positive and EBV-negative tumor regions. **(C)** Volcano plot of memory (CD45RO+) vs. naive (CD45RO-) T cells, with some of the most differentially expressed genes shown. *CD8A* and *LAG3* are indicated by the green arrows. The differential gene expression data for each capture region are in **Supp Table 11**.

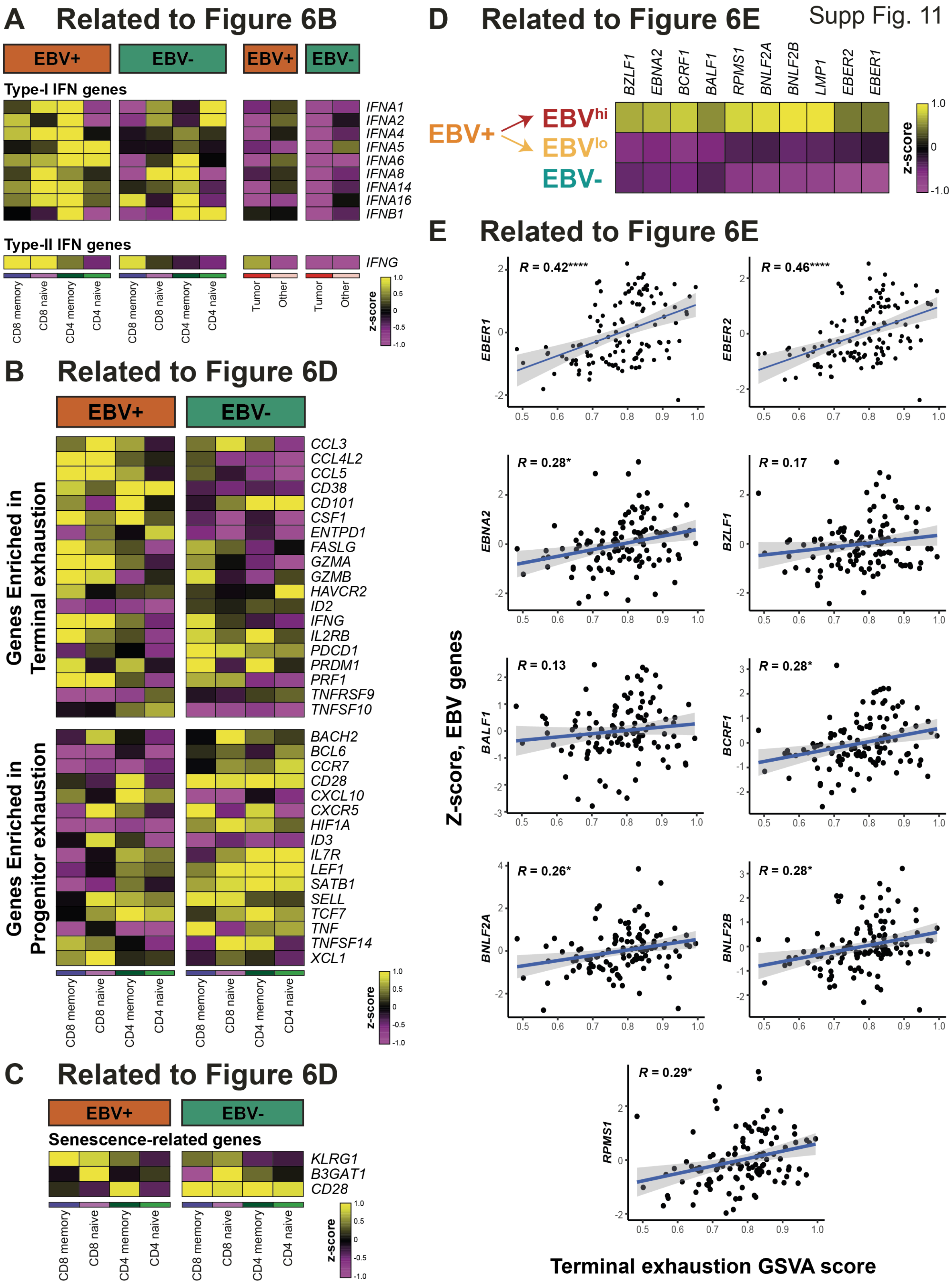

Figure S11, related to Figure 6. Expressions of genes used to score specific gene sets. (A) Expression heatmap of interferons used to score "Type-I IFN genes" and "Type-II IFN genes" pathways in **Fig. 6B**. This list does not include all known interferons because it only contains probes available in the GeoMx probe set. **(B)** Expression heatmap of key differentially expressed genes between terminal and progenitor T-cell exhaustion states (see Fig. 2D in (3)). **(C)** Expression heatmap of genes and pathways associated with T-cell senescence (4). **(D)** Spearman correlation of each EBV transcript with T-cell terminal exhaustion GSVA score. **(E)** Expression of each EBV transcript upon stratifying EBV-positive samples into EBV-high and EBV-low categories.

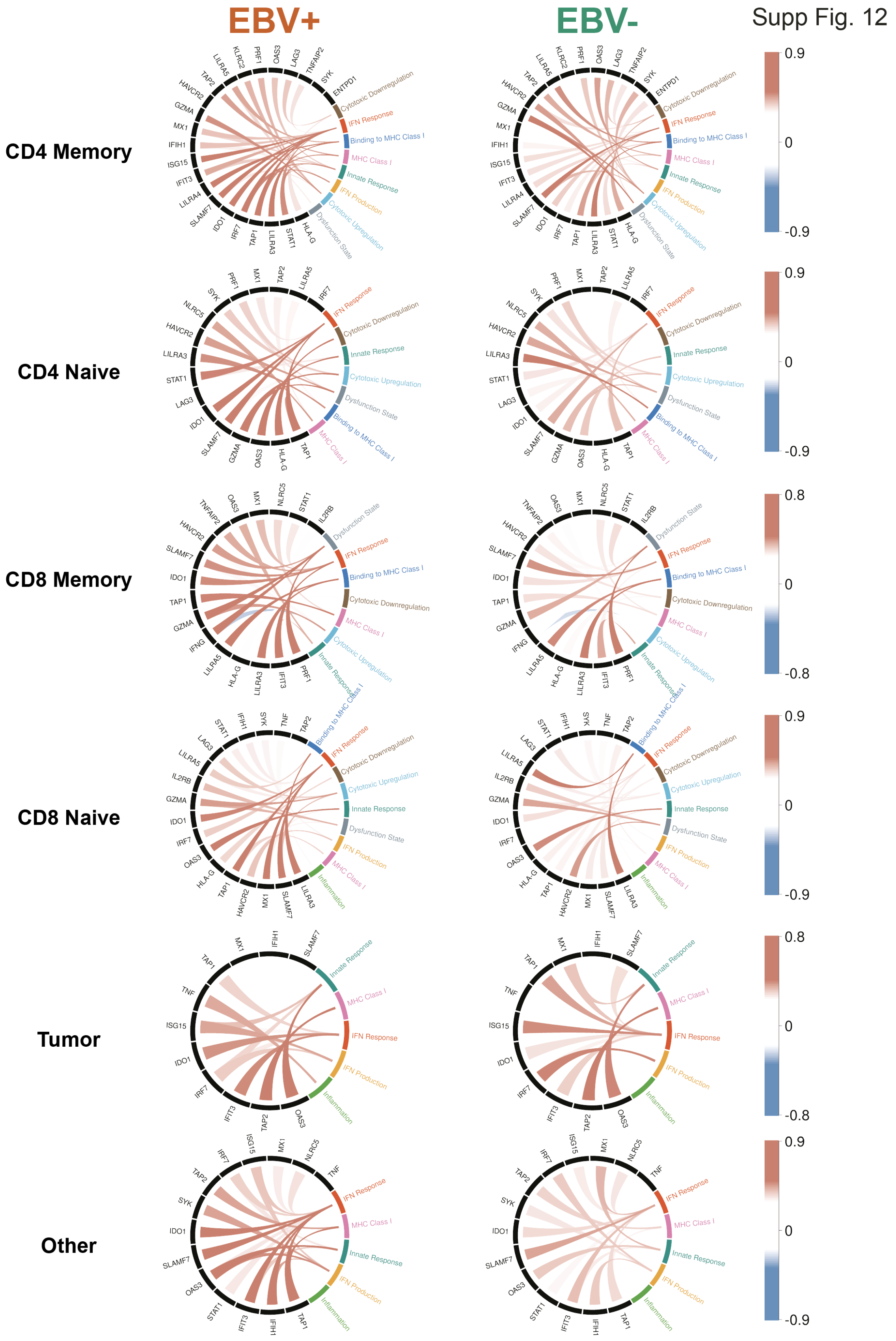

Figure S12, related to Figure 6. Comparison of representative gene-pathway correlations for each cell population, stratified by EBV status. Circos plot for each cell population profiled with the GeoMx.

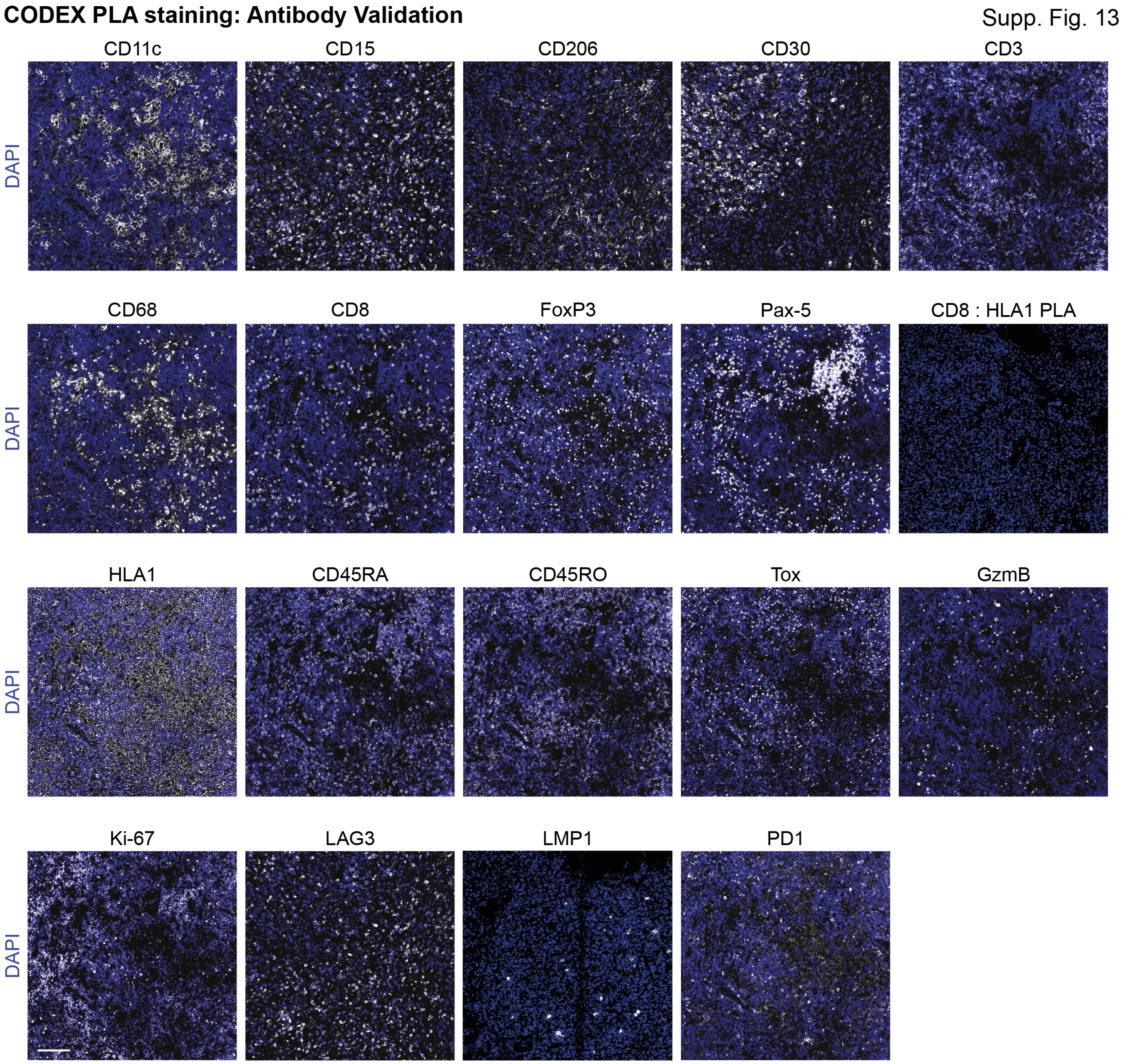

Figure S13, related to Figure 7. Validation of CODEX staining specificity. Representative CODEX images across cHL tissue sections, showing each (white) overlaid with the cell nucleus dye DAPI (blue). Scale bar: 100 µm.

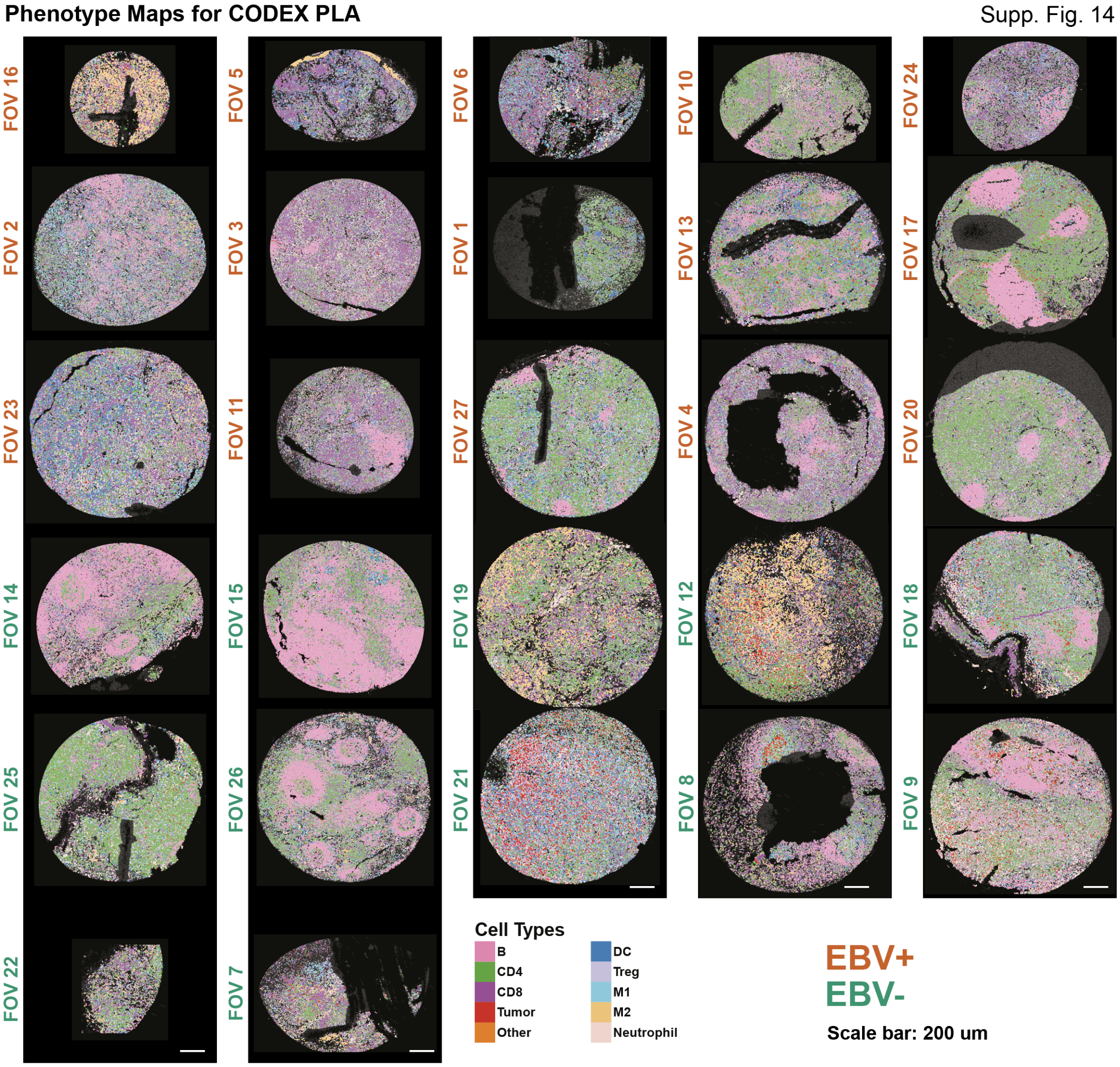

Figure S14, related to Figure 7. Validation of cell phenotyping from CODEX images. Phenotype maps of all the CODEX acquired FOVs across cHL TMA, generated through iterative clustering and annotation based on single-cell CODEX features. Scale bar: 200 µm.

A Related to Figure 7B

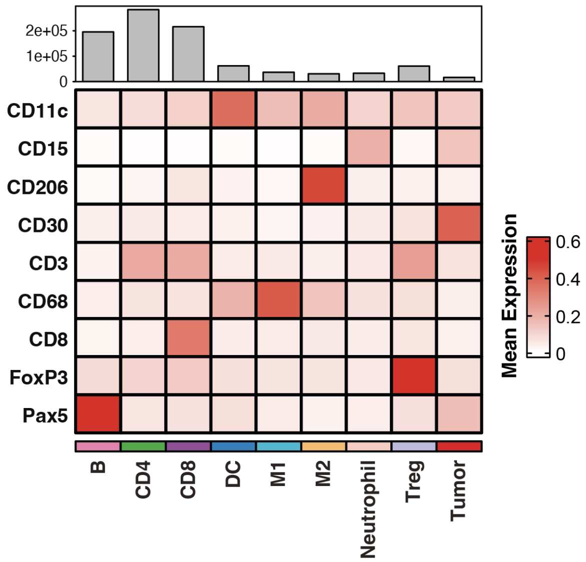

B Related to Figure 7F

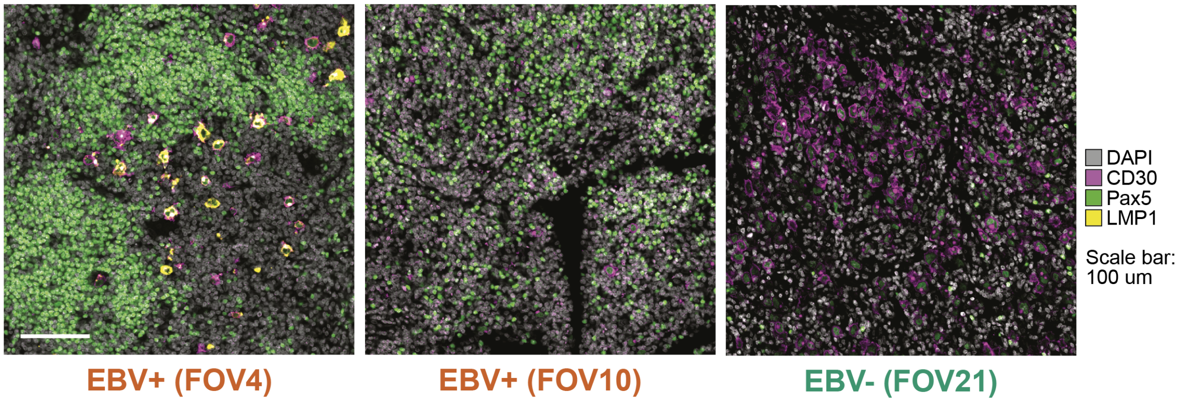

Figure S15, related to Figure 7. Validation and quantification of marker expression. (A) Relative mean expression levels of phenotypic markers for the annotated cell phenotypes across the CODEX-PLA dataset. **(B)** Representative images showing high and low abundance of LMP1 expression in HRS cells within EBV-positive cHL, as well as the lack of LMP1 staining in EBV-negative cHL.

Reference

1. Daisy Yi Ding, Zeyu Tang, Bokai Zhu, Hongyu Ren, Alex K Shalek, Robert Tibshirani, and Garry P Nolan. Quantitative characterization of tissue states using multiomics and ecological spatial analysis. *Nature Genetics*, pages 1–12, 2025.
2. Z. Chen, I. Soifer, H. Hilton, L. Keren, and V. Jojic. Modeling multiplexed images with spatial-lda reveals novel tissue microenvironments. *J Comput Biol*, 27(8):1204–1218, 2020. doi: 10.1089/cmb.2019.0340.
3. BC Miller, DR Sen, RA Abosy, K Bi, YV Virkud, MW LaFleur, KB Yates, A Lako, K Felt, GS Naik, and et al. Subsets of exhausted cd8+ t cells differentially mediate tumor control and respond to checkpoint blockade. *Nat Immunol*, 20(3):326–336, 2019. doi: 10.1038/s41590-019-0312-6.
4. EJ Wherry and M Kurachi. Molecular and cellular insights into t cell exhaustion. *Nat Rev Immunol*, 15(8):486–499, 2015. doi: 10.1038/nri3862.
