# Supplementary material for: Epstein-Barr virus orchestrates spatial reorganization and immunomodulation in the classic Hodgkin lymphoma tumor microenvironment
Source: Cell Rep Med. 2026 Mar 31;7(4):102722. doi: 10.1016/j.xcrm.2026.102722 (PMC13130692; doi:10.1016/j.xcrm.2026.102722)
Supplement: Document S2. Article plus supplemental information [file mmc13.pdf]

Cell Reports Medicine

Article

Epstein-Barr virus orchestrates spatial reorganization and immunomodulation in the classic Hodgkin lymphoma tumor microenvironment

Graphical abstract

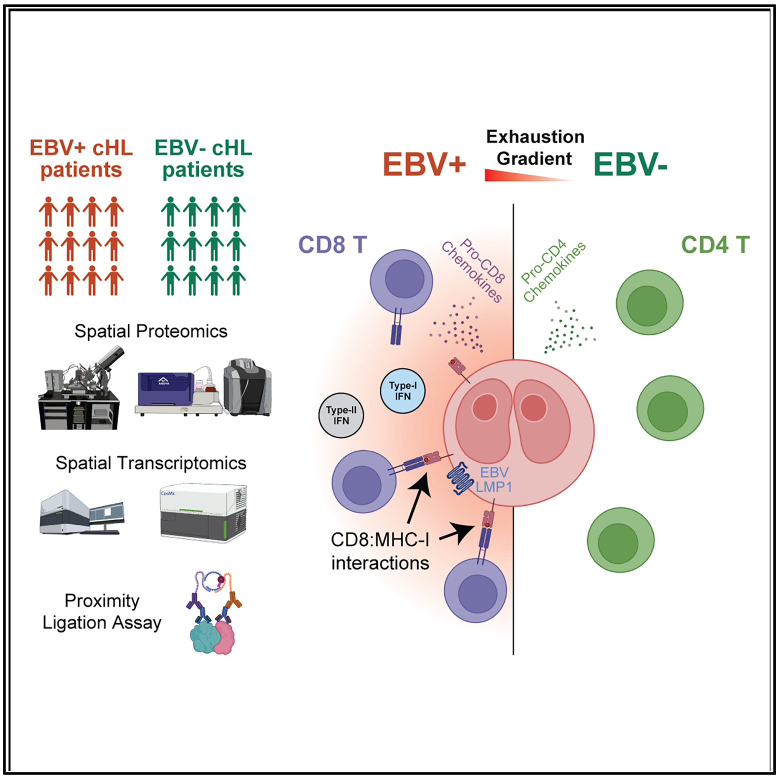

Authors

Yao Yu Yeo, Huaying Qiu, Yunhao Bai, ..., Margaret A. Shipp, Scott J. Rodig, Sizun Jiang

Correspondence

sjiang3@bidmc.harvard.edu

In brief

Yeo et al. use spatial multi-omics to reveal EBV-driven remodeling of the classic Hodgkin lymphoma microenvironment. EBV-positive disease shows CD8 T cell enrichment with terminal exhaustion linked to LMP1 and MHC class I on HRS cells, contrasting progenitor exhaustion in EBV-negative disease.

Highlights

- Spatial-omics reveal contrasting CD8-T-enriched EBV+ cHL versus CD4-T-enriched EBV− cHL
- EBV+ cHL exhibits terminal T cell exhaustion; EBV− disease shows progenitor exhaustion
- T cell dysfunction correlates with proximity to HRS cells and EBV expression
- LMP1 and MHC class I on HRS cells augment CD8 T cell terminal exhaustion

Yeo et al., 2026, Cell Reports Medicine 7, 102722
April 21, 2026 © 2026 The Author(s). Published by Elsevier Inc.
https://doi.org/10.1016/j.xcrm.2026.102722

CellPress

Cell Reports Medicine

CellPress
OPEN ACCESS

Article

Epstein-Barr virus orchestrates spatial reorganization and immunomodulation in the classic Hodgkin lymphoma tumor microenvironment

Yao Yu Yeo,[1,2,22] Huaying Qiu,[1,22] Yunhao Bai,[3,4,22] Bokai Zhu,[3,5,22] Yuzhou Chang,[1,6] Fabio Iannelli,[7] Stephanie Pei Tung Yiu,[1] Jason Yeung,[1] Hendrik A. Michel,[1,2] Yuchen Wang,[1] Yang Wang,[1] Wenrui Wu,[1] Kyle Wright,[8] Muhammad Shaban,[8,9] Sam Sadigh,[8] Dingani Nkosi,[10] Vignesh Shanmugam,[8,9] Philip Rock,[10] Precious Cramer,[1] Julia Paczkowska,[11] Pierre Stephan,[1,8,12] Guanrui Liao,[1] Amy Y. Huang,[8] Hongbo Wang,[13] Han Chen,[3] Leonie Frauenfeld,[14] Louisa Kaufmann,[14] Stefano Pileri,[7] Bidisha Mitra,[13] Benjamin E. Gewurz,[2,13] Bo Zhao,[13] Garry P. Nolan,[3] Baochun Zhang,[11] Alex K. Shalek,[9,16] Michael Angelo,[3] Christian M. Schürch,[14,15] Faisal Mahmood,[8,9] Roberto Chiarle,[7,17,18] Qin Ma,[6,19] W. Richard Burack,[10] Margaret A. Shipp,[11] Scott J. Rodig,[8,21] and Sizun Jiang[1,2,8,9,20,21,23,*]

[1]Center for Virology and Vaccine Research, Beth Israel Deaconess Medical Center, Harvard Medical School, Boston, MA, USA
[2]Program in Virology, Division of Medical Sciences, Harvard Medical School, Boston, MA, USA
[3]Department of Pathology, Stanford University, Stanford, CA, USA
[4]Department of Chemistry, Stanford University, Stanford, CA, USA
[5]Department of Microbiology and Immunology, Stanford University, Stanford, CA, USA
[6]Pelotonia Institute for Immuno-Oncology, The Ohio State University, Columbus, OH, USA
[7]Division of Haematopathology, European Institute of Oncology IRCCS, Milan, Italy
[8]Department of Pathology, Brigham and Women's Hospital, Harvard Medical School, Boston, MA, USA
[9]Broad Institute of Harvard and MIT, Cambridge, MA, USA
[10]Department of Pathology and Laboratory Medicine, University of Rochester Medical Center, Rochester, NY, USA
[11]Department of Medical Oncology, Dana-Farber Cancer Institute, Harvard Medical School, Boston, MA, USA
[12]Université de Paris, Assistance Publique-Hôpitaux de Paris, Hemato-oncologie, Saint-Louis Hôspital, Paris, France
[13]Division of Infectious Disease, Department of Medicine, Brigham and Women's Hospital and Harvard Medical School, Boston, MA, USA
[14]Department of Pathology and Neuropathology, University Hospital and Comprehensive Cancer Center Tübingen, Tübingen, Germany
[15]Cluster of Excellence iFIT (EXC 2180) "Image-Guided and Functionally Instructed Tumor Therapies", University of Tübingen, Tübingen, Germany
[16]Department of Chemistry, Massachusetts Institute of Technology, Cambridge, MA, USA
[17]Department of Molecular Biotechnology and Health Sciences, University of Torino, Torino, Italy
[18]Department of Pathology, Boston Children's Hospital, Harvard Medical School, Boston, MA, USA
[19]Department of Biomedical Informatics, The Ohio State University College of Medicine, Columbus, OH, USA
[20]Department of Pathology, Dana-Farber Cancer Institute, Boston, MA, USA
[21]Senior author
[22]These authors contributed equally
[23]Lead contact
*Correspondence: sjiang3@bidmc.harvard.edu
https://doi.org/10.1016/j.xcrm.2026.102722

SUMMARY

Classic Hodgkin lymphoma (cHL) is composed of rare malignant Hodgkin and Reed-Sternberg (HRS) cells within a T-cell-rich tumor microenvironment (TME). Epstein-Barr virus (EBV) is present in ∼25% of cases, but its contribution to pathogenesis and immunomodulation remains unclear due to technical barriers. Using complementary spatial proteomics and transcriptomics across multi-institutional cohorts, we systematically map key EBV-linked TME reorganization. EBV-positive cHL exhibits distinct immunological features, including memory CD8 T cell enrichment, heightened T cell dysfunction spatially correlated with HRS proximity, and terminally exhausted T cell signatures contrasting with progenitor-exhausted patterns in EBV-negative disease. We identify EBV-encoded LMP1 as a factor in T cell dysfunction through enhanced HRS:CD8 interactions, and its expression level correlates with T cell terminal exhaustion in a distance-dependent manner. This spatial framework dissects viral-mediated immune evasion in the cHL TME, highlighting potential therapeutic opportunities to target virus-associated T cell dysfunction for precision immunotherapy in virus-associated malignancies.

© 2026 The Author(s). Published by Elsevier Inc.
This is an open access article under the CC BY license (http://creativecommons.org/licenses/by/4.0/).

INTRODUCTION

Classic Hodgkin lymphoma (cHL) is a B cell lymphoid malignancy that most frequently occurs in young adults and less commonly in the elderly.[1] The cHL tumor microenvironment (TME) has unique features, including rare malignant Hodgkin and Reed-Sternberg (HRS) multinucleated cells (comprising 1%–5% of the cHL TME cell composition) nested within a dense T-cell-rich inflammatory immune cell infiltrate. Although the etiology of cHL remains to be defined, cHL is associated with the oncogenic Epstein-Barr virus (EBV) in ~25% of cases.[2–4] Of note, cHL has lower cure rates and is more frequently EBV-positive in older patients.[1] It remains elusive as to whether this is due to poorer tolerance of aggressive chemotherapy or inherent biological differences associated with EBV infection.

EBV-positive and EBV-negative HRS cells are morphologically indistinguishable, but emerging evidence suggests that the cHL TME is modulated by the presence of the virus.[5] EBV generally establishes latency II in HRS cells to express oncoproteins, including latent membrane protein 1 (LMP1), LMP2A, and EBV nuclear antigen 1 (EBNA1). LMP1 and EBNA1 expression in HRS cells is associated with the upregulation of immunomodulatory cytokines including CXCL10,[6] interleukin (IL)-6,[7] IL-10,[8,9] and CCL20,[10] secreted factors that can significantly alter immune cell responses and organization within the TME.[11] EBV-positive HRS cells also retain major histocompatibility complex (MHC) class I expression more frequently than their virus-negative counterparts, which often have a genetic basis for beta-2 microglobulin (B2M) loss and a higher mutational burden.[12–15] These results are consistent with the retention of MHC class I on EBV-positive HRS cells to present latent EBV oncoprotein antigens[16] and potential increase in CD8 T cell activation within the EBV-positive cHL TME.[17] Furthermore, virus-driven cHL was recently proposed as a distinct subtype of cHL associated with different clinical outcomes from virus-negative cHL based on circulating tumor DNA,[18] supporting a distinctive TME between EBV-positive and EBV-negative cHL.

To determine how EBV immunoorchestrates immunomodulation and reorganization of the cHL TME, we utilized multiplexed ion beam imaging (MIBI)[19] and co-detection by indexing (CODEX)[20] spatial proteomics, as well as NanoString GeoMx[21] and CosMx[22] spatial transcriptomics, to interrogate and delineate tumor-immune interactions within the native TME. We define distinct immunosuppression mechanisms in EBV-positive and EBV-negative cHL associated with different T cell dysfunction states. These findings establish EBV as a critical modulator of the cHL TME, which may inform immunotherapeutic strategies in EBV-positive cHL and other viral-associated malignancies.

RESULTS

Spatial proteomics to interrogate differential TME responses between EBV-positive and EBV-negative cHL

We developed and applied a 30-plex MIBI antibody panel (Table S1) with phenotyping and functional markers to archival excisional FFPE tumor tissues from cHL patients (6 EBV-positive, 14 EBV-negative; Table S2) for cell-type annotation and cellular neighborhood (CN) analysis of EBV-linked tumor-immune responses (Figure 1A). Antibodies were titrated and optimized in reactive lymph nodes and cHL cases (Figures 1B and S1) as previously described.[23–25] The panel included markers for M2-like macrophages (CD163), B cells ($Pax5^{hi}$), HRS cells ($Pax5^{lo}$), dendritic cells (DCs) (CD11c), PD-L1, and T cell subsets defined by lineage (CD4, CD8), memory (CD45RO), and dysfunction (Tox) markers (Figure 1B); representative staining for all 30 markers is shown in Figure S1.

Immune components and higher T cell dysfunction signatures distinguish EBV-positive from EBV-negative cHL TME

We next performed cell segmentation (Table S3) and clustering across all 20 patients as previously described,[23–25] analyzing 41 stitched field of views (FOVs) comprising 794 tiles of 400 × 400 μm images selected by expert hematopathologists using adjacent H&Es (STAR Methods). Cell annotations (Figure S2) were confirmed on the original MIBI images, and key phenotypic markers showed expected enrichment across annotated cell types (Figure 2A, left; Figure S3A). Background signal due to lateral spillover, a known feature of spatial proteomics data,[26] did not impact cell quantitation, as exemplified by apparent CD3/CD4 signal on DCs and programmed death-ligand1 (PD-L1) signal on neutrophils arising from proximity to marker-positive cells (Figure S3B).

We observed relative differences in the TME composition between EBV-positive and EBV-negative cHL TMEs (Figure 2A, right) and further quantified the differences in cell-type proportions (Figure 2B) and enrichment (Figure 2C). Of note, there were significantly higher proportions of CD8 T cells in the EBV-positive TME, in stark contrast with the significant increase of CD4 T cell and neutrophil proportions in the EBV-negative cHL TME (Figures 2A–2C). This result is also consistent when evaluated on individual FOV levels (Figure 2D). Our data also revealed the prevalence of M2-like macrophages and regulatory T cells (Tregs) in the EBV-positive TME (Figure 2C) and observed a generally higher number of HRS cells in EBV-negative cHLs (Figure 2C).

The enrichment of CD8 T cells with impaired effector function implicates EBV in conditioning the cHL TME to suppress T cell responses. To test this, we quantified MHC class I (B2M and HLA1) and class II (HLA-DR) expression across the imaged FOVs (Figure 2E). EBV-positive cHL exhibited significantly higher MHC class I expression across immune and tumor cell types, including HRS cells, whereas MHC class II expression did not differ significantly by EBV status (Figures 2E and S3C). Given that MHC class I is broadly expressed by non-neoplastic cells and a subset of HRS cells,[15] mean expression heatmaps and raw image inspection confirmed a tissue-level increase in MHC class I expression in EBV-positive TMEs beyond HRS cells (Figures S3D and S3E). These findings extend prior reports of EBV-associated differences in antigen presentation[12,13,15,16,27] and reveal global EBV-linked remodeling of antigen presentation across the cHL TME.

The cHL TME is enriched in PD-L1,[27,28] implicating $PD1^{+}$ T cells as key immunosuppressive targets. We therefore examined memory (CD45RO[29]) and checkpoint regulators (Tox,[30–33]

CellPress
OPEN ACCESS

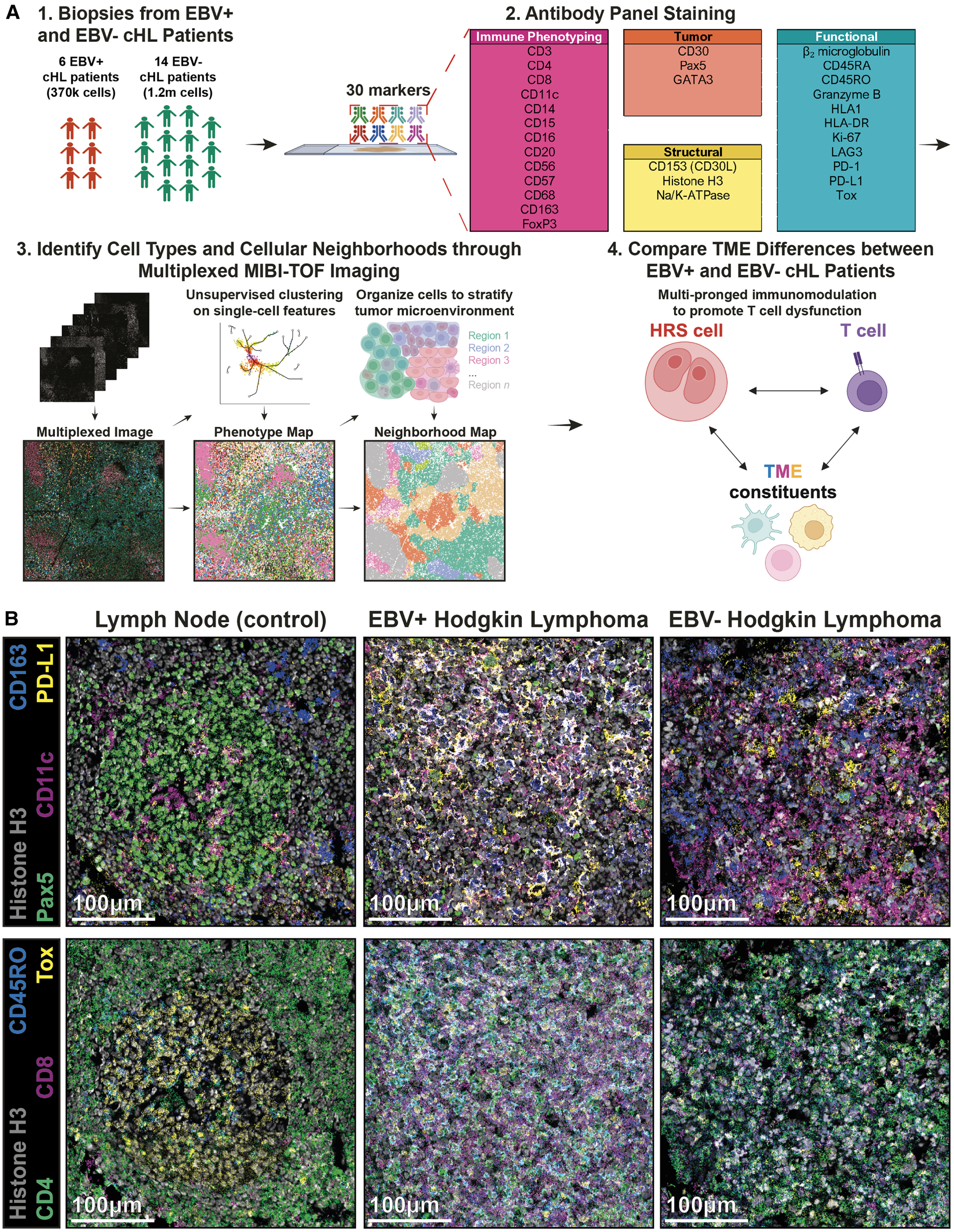

Figure 1. An overview of the spatial-omics framework to interrogate differences between EBV-positive and EBV-negative cHL TME

(A) Study overview to spatially dissect the EBV-positive and EBV-negative cHL TME. (1) Biopsies from the initial cohort of EBV-positive (number of patients = 6, number of FOVs = 19) and EBV-negative (number of patients = 14, number of FOVs = 23) patients were collected, (2) stained with 30 lanthanide-conjugated

(legend continued on next page)

Lag3,[34] and programmed cell death protein 1 [PD-1][35]) on T cells. Although CD8 and CD4 T cell dysfunction states differ molecularly,[36] Lag3 and PD-1 are shared features of dysfunction,[36,37] and Tox was used as an additional proxy for CD4 T cell dysfunction. EBV-positive TMEs showed increased CD45RO and dysfunction markers (Tox and Lag3) on CD4 T cells but not PD-1 (Figure 2F, **top**; Figure S3F). PD-L1 was also elevated on HRS cells and antigen-presenting cells (DCs, M1-like, and M2-like macrophages) in EBV-positive TMEs (Figure 2F, bottom; Figure S3F). This is consistent with EBV-mediated PD-L1 upregulation, as LMP1 knockdown reduced *CD274* transcripts (Figure S3G). Mean expression heatmaps confirmed PD-L1 expression on HRS and antigen-presenting cells[27,28] and T cell dysfunction markers[38,39] irrespective of EBV status (Figure 2F, **bottom**; Figure S3H). Stratification by CD45RO/CD45RA further demonstrated enrichment of dysfunction (Tox, Lag3, and PD-1) and proliferation (Ki67) markers in memory T cells (Figure S3I).

These results expand upon previous observations regarding PD-1 and Lag3 expression on CD4 T cells and PD-L1 on macrophages within the immunoprivileged cHL TME[39] to include increased M2-like macrophage and Treg populations (Figure 2C) in the EBV-positive cHL TME and a paired increase of PD-1 and PD-L1 between T cells, HRS cells, and surrounding immune constituents to coordinate EBV-linked immune dysregulation of the cHL TME. These results suggest an EBV-dependent mechanism for suppressing T cell effector functions through reorganization and re-education of the cHL TME.

Tumor spatial heterogeneity and cellular-neighborhood-specific T cell dysfunction and antigen presentation in the cHL TME

We next compared the spatial organization of EBV-positive and EBV-negative cHL TMEs by quantifying cellular diversity using ecology-inspired indices, including the Shannon index[40] and MESA spatial biodiversity metrics[41] (Figures 3A, left; Figure S4). While Shannon diversity did not differ by EBV status, EBV-positive TMEs exhibited significantly lower MESA Diversity Proximity and Global Diversity indices (Figure 3B, right). These findings indicate that although overall cellular diversity is comparable, EBV-positive TMEs are spatially less compartmentalized and more locally homogenized than EBV-negative tissues.

We defined cellular neighborhoods (CNs) using spatial latent Dirichlet allocation[42] and identified eight CNs across EBV-positive and EBV-negative cHL samples (Figure 3B, left; Figure S5). HRS cells localized primarily to CN-0, CN-1, and CN-6, each characterized by distinct T cell compositions. CN-0 (tumor rich and immune rich) exhibited diverse immune populations, CN-1 (tumor low and immune active 1) had fewer CD8 T cells, and CN-6 (tumor low and immune active 2) was largely T-cell-poor except for Tregs. M2-like macrophages were enriched in CN-0 relative to CN-1 and CN-2 (Figure 3B, right). Stratification by EBV status showed CN-0 dominance in EBV-positive cHL, whereas CN-1 and CN-6 were more prevalent in EBV-negative disease (Figure S6A), consistent with diversity index analyses.

As HRS cells were most abundant in CN-0 followed by CN-1 (Figure 3B, right), with CN-0 enriched in EBV-positive TMEs and CN-1 enriched in EBV-negative TMEs (Figure S6A), we focused subsequent analyses on these two CNs (Figure 3C). In CN-0, EBV-positive TMEs showed enrichment of CD8 and CD8 CTL populations, whereas EBV-negative TMEs were enriched for CD4 T cells (Figure 3D), consistent with prior observations (Figures 2C and 2D). Other immune and stromal populations, including HRS cells, Tregs, DCs, macrophages, and endothelial cells, were largely comparable between EBV-positive and EBV-negative TMEs, with increased B cells and reduced neutrophils and natural killer (NK) cells in EBV-positive CN-0 regions (Figures 3D and S6B). In CN-1, immune composition was similarly conserved, apart from a modest increase in M2-like macrophages in EBV-positive TMEs (Figures 3D and S6B). The limited differences in HRS cell abundance across CNs (Figure 3D) indicate that EBV-linked immune reprogramming reflects altered functional states rather than tumor cell quantity alone.

Given PD-L1 enrichment in the cHL TME[27,28] and EBV-linked T cell dysfunction (Figure 2F), we compared antigen presentation, T cell activation, and dysfunction states between CN-0 and CN-1 (Figures 3D, S6B, and S6C). CN-0, particularly in EBV-positive TMEs, exhibited increased MHC class I (HLA1 and B2M), T cell memory (CD45RO), dysfunction markers (Tox, Lag3, and PD-1), and PD-L1 expression on HRS and antigen-presenting cells (DCs and macrophages). Mean expression heatmaps confirmed these features across CN-0 and CN-1 (Figure S6E). Together, these findings support a model in which HRS cells shape their local neighborhoods to promote T cell dysfunction, with EBV-positive tumors further enhancing MHC-I-associated antigen presentation and TME reorganization.

Identification of immune dysfunction as a function of spatial organization around HRS cells in the cHL TME

Our results suggest that HRS cells modulate immune dysfunction within their local microenvironment (Figures 3D and 3E). We therefore tested whether immune dysfunction depends on proximity to HRS cells by developing a distance- and density-weighted "Tumor Score" metric for immune cells within 100 μm (Figure 4A). Cells were stratified into "tumor dense" and "tumor sparse" regions using a global cutoff (Figures 4A, 4B, S7, S8, and S9A; see STAR Methods). Although EBV-positive TMEs contain fewer tumor cells overall (Figures 2C and 2D), this approach standardizes regional classification across samples, enabling EBV-independent comparison of spatial tumor effects.

We next assigned each immune cell a "Dysfunction Score," negatively weighting activation markers (CD45RA and Ki-67)

antibodies, and acquired by the MIBI spatial proteomics platform to (3) generate multiplexed images for the identification of cell phenotypes and neighborhoods, where upon (4) computational analysis illustrated key differences between EBV-positive and EBV-negative cHL TME.

(B) Representative MIBI images of lymph node (left), EBV-positive (middle), and EBV-negative (right) sections, with markers for nuclei (Histone H3), M2-like macrophages (CD163), HRS cells (Pax5^{lo}), dendritic cells (CD11c), programmed death-ligand 1 (PD-L1), memory T cells (CD45RO), CD4 T cells (CD4), CD8 T cells (CD8), and T-cell-dysfunction-associated transcription factor (Tox). Scale bars: 100 μm.

CellPress
OPEN ACCESS

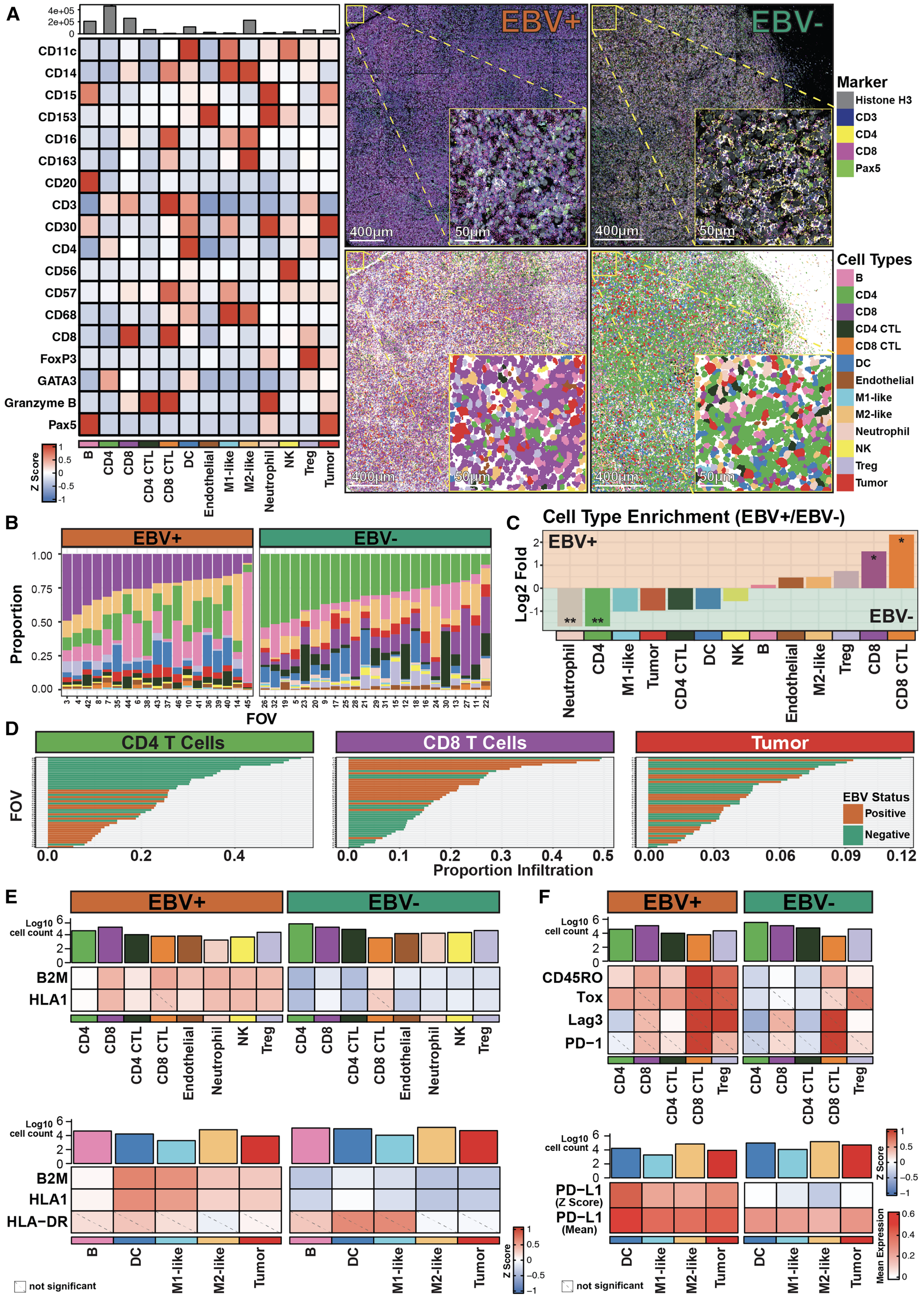

(legend on next page)

and positively weighting dysfunction-associated markers (CD45RO, Tox, PD-1, and Lag3). For HRS cells and antigen-presenting cells (DCs and macrophages), PD-L1 expression was also included (see STAR Methods).[37] Dysfunction scores were compared between tumor dense and tumor sparse regions defined by Tumor Scores, revealing significantly higher dysfunction in tumor dense regions across both EBV-positive and EBV-negative TMEs (Figures 4C and S9B), affecting both CD8 and CD4 T cells. Proximity-linked dysfunction was further elevated in EBV-positive regions relative to EBV-negative counterparts (Figures 4C and S9B), supporting a central role for HRS cells in modulating immune cell states.

Our data support a model in which EBV enhances T cell dysfunction and reorganizes the cHL TME relative to EBV-negative disease (Figure 4D). EBV-positive TMEs exhibit increased MHC class I presentation (B2M and HLA1) and a higher proportion of dysfunctional memory CD8 T cells, whereas EBV-negative TMEs show enriched CD4 T cells and greater HRS cell density. Proximity-dependent modulation of immune cells near HRS cells further amplifies dysfunction, accompanied by EBV-associated upregulation of Tox, Lag3, PD-1, and PD-L1, potentially through direct chromatin regulation by EBV transcription factors.[43]

Dissection of immune and HRS cells in the EBV-linked cHL TME through spatial multi-omics on a multi-institutional cohort

As T cell dysfunction was most pronounced near HRS cells (Figure 4C), we applied a spatial multi-omics strategy to refine HRS-T-cell interactions in the cHL TME. In a multi-institutional cohort (22 EBV-positive and 24 EBV-negative; Table S2), GeoMx genome-wide transcriptomic profiling[21] was performed on 62 EBV-positive and 62 EBV-negative regions of interest (ROIs) enriched for HRS cells across six cell populations (tumor-rich, CD4 naive, CD4 memory, CD8 naive, CD8 memory, and others) (Figure 5A; see STAR Methods). These populations were defined by combinatorial antibody staining (Table S1), single-cell segmentation (Table S3), and clustering (Table S5), with representative staining and annotations shown in Figure 5B.

Given the regional (non–single-cell) resolution of GeoMx and the potential presence of CD45RA-reexpressing effector memory T cells (Temra),[44] we verified that annotated regions were enriched for the intended cell populations. Consistent with MIBI results (Figure 2C, top), EBV-positive cHL showed enrichment of CD8 T cells, whereas EBV-negative cHL showed increased CD4 and tumor cells (Figure 5C, top). Cell-type specificity was further supported by differential gene expression, including enrichment of *TNFRSF8*, *EBER1*, and *EBER2* in tumor-enriched regions; T cell lineage markers (*CD3D*, *CD3E*, *CD4*, *CD7*, *CD8A*, and *CD8B*) in CD4 and CD8 regions; cytotoxic transcripts (*PRF1*, *GZMK*, *GZMA*, and *GZMH*) in memory T cell regions (Figure S10A); and B cell transcripts (*CD19*, *MS4A1*, *CD79A*, *IGH*s, *IGL*s, and *IGK*s) in the "Others" region (Figure 5C, bottom). EBV-positive tissues also showed specific detection of EBV transcripts, including *EBER1*, *EBER2*, *LMP1*, and additional latent and lytic genes (*EBNA2*, *BALF1*, *BCRF1*, *BNLF2A*, *BNLF2B*, *BZLF1*, and *RPMS1*) (Figure S10B), consistent with prior bulk transcriptomic studies.[45]

Differential gene expression analysis between memory and naive T cells, defined by CD45RO, showed enrichment of *LAG3* transcripts in memory T cells (Figure S10C), consistent with MIBI findings (Figure S3H) and its role as an exhaustion marker in CD8 and CD4 T cells.[36] Stratification by EBV status further revealed enrichment of *CD8A*, *CD8B*, and *LAG3* in memory T cell populations in EBV-positive tissues (Figure 5D, top), with no clear differences in EBV-negative tissues (Figure 5D, bottom); EBV-associated *LAG3* enrichment remained consistent within CD8 and CD4 subsets. These findings align with MIBI data showing predominance of memory CD8 T cells with enhanced dysfunction in EBV-positive TMEs (Figures 2F and 3E). Memory T cells also displayed elevated cytotoxic transcripts, including *GZMA*, *GZMH*, and *GZMK* (Figure 5D), supporting concurrent effector features within dysfunctional populations.

We next analyzed EBV-linked transcriptomic differences across tumor, CD8 T, and CD4 T cell populations (Figure 5C, top; Figure 2C). EBV-positive tumors showed reduced density associated with lower G2M cell-cycle activity (Figure 5E, left), consistent with fewer Ki67$^+$ HRS cells in MIBI data (Figure 5E,

Figure 2. Distinct immune components and T cell dysfunction features between EBV-positive and EBV-negative cHL TME

(A) Relative *Z* score expression levels of phenotypic markers (left) for the annotated cell phenotypes in this MIBI dataset; a mean expression visualization is in Figure S3A. Representative MIBI images of EBV-positive (middle, top) and EBV-negative cHL FOVs (right, top), with markers for nuclei (Histone H3), T (CD3), CD4 T (CD4), CD8 T (CD8), B (Pax5^{hi}), and HRS (Pax5^{lo}) cells, along with the corresponding EBV-positive (middle, bottom) and EBV-negative cell phenotype maps (right, bottom). Phenotype maps for the other FOVs are in Figure S2. Scale bars: 400 μm (main) and 50 μm (inset).

(B) Relative proportions of annotated cell types across EBV-positive and EBV-negative cHL FOVs.

(C) Log2 fold enrichment plot of annotated cell types between EBV-positive and EBV-negative cHL FOVs. Significance stars are only shown for significant comparisons. Two-sided Wilcoxon tests were conducted for all cell type proportions; test results were adjusted for multiple comparisons using the Benjamini-Hochberg (BH) method with a targeted false discovery rate (FDR) at 0.05. Unadjusted *p* value and BH corrected test results are in Table S4.

(D) Relative proportion infiltration of CD4 T, CD8 T, and HRS cells across all FOVs.

(E) Top: relative expression of the MHC class I (B2M and HLA1) on annotated cell types, apart from antigen-presenting cells and HRS cells, in the cHL TME. Bottom: Relative expression of MHC class I and MHC class II (HLA-DR) on antigen-presenting cells and HRS cells in the cHL TME. Representative MIBI images of MHC class I differences are in Figure S3E.

(F) Top: relative expression of memory (CD45RO) and T cell dysfunction (Tox, Lag3, and PD-1) markers on T cell populations, with the relative cell counts inscribed above. Bottom: relative expression of PD-L1 on antigen-presenting cells (DCs and macrophages) and HRS cells, with the cell counts inscribed above; both *Z* score and mean expression representations are here. Statistical test results for (E) and (F) are in Figures S3C and S3F. Mean expression visualization for (E) and (F) heatmaps are in Figures S3D and S3H. All statistical tests were performed by comparing EBV-positive to EBV-negative populations, and unadjusted *p* value and BH corrected test results are in Table S4. Significant stars: *$p \leq 0.05$, **$p \leq 0.01$.

CellPress
OPEN ACCESS

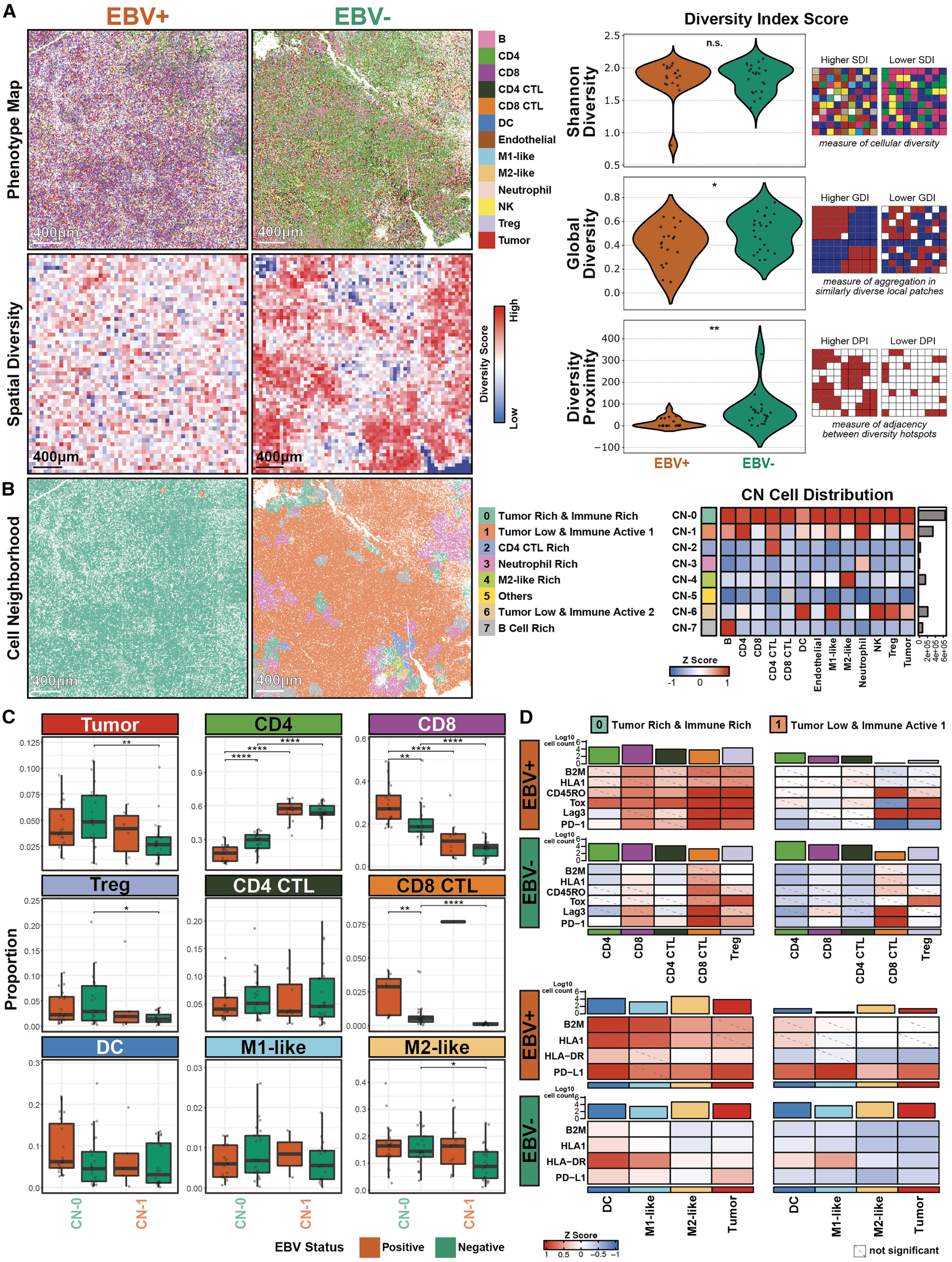

(legend on next page)

right). Differential expression analysis of tumor-rich regions revealed enrichment of *CXCL9*, a CD8-T-cell-recruiting chemokine,[46] in EBV-positive regions and enrichment of *CCL17*, which recruits CD4 T cells,[47,48] in EBV-negative regions (Figure 5F). Receptor-ligand interaction analysis[49,50] further demonstrated enhanced CD8-T-cell-attracting chemokine signaling in EBV-positive TMEs, including the *CXCR3-CXCL9/10/11*[46] and *CXCR6-CXCL16*[51] axes (Figure 5G, top), whereas CD4-T-cell-associated signaling via the *CCR4–CCL17/22* axis[47,48] was reduced in EBV-positive TMEs (Figure 5G, middle). Engagement of *CCR5-CCL5/7/8* interactions was also increased in EBV-positive tumor-CD8 T cell contexts (Figure 5G, bottom).[52,53]

Systems-level inflammatory responses and terminal T cell dysfunction state in the EBV-positive cHL TME

Elevated CD8 T cell infiltration and MHC class I presentation in EBV-positive cHL suggest enhanced antiviral and inflammatory responses. We therefore computed global correlations of gene sets categorized as "antiviral responses" and "T cell dysfunction" in EBV-positive and EBV-negative spatial transcriptomic data. EBV-positive TMEs exhibited coordinated enrichment of these pathways (Figure 6A), indicating a distinct EBV-associated immunological landscape.

We further delineated the enrichment scores of these associated gene sets stratified by cell population and EBV status to identify a tissue-level increase in immune responses linked to an antiviral state, including innate cellular immune responses, chronic inflammation, secretion and response to both type I and type II interferons (IFNs) (Figure S11A), and the expression of MHC class I (Figure 6B). The role of these pathways in promoting the expression of MHC class I[54] implicates them in our observations of tissue-level increase in MHC class I (Figure 2E).

Our cumulative data and prior results[55] implicate T cell dysfunction likely through unique EBV-linked mechanisms. We observed that although T cells in the EBV-positive cHL TME have higher expression of molecules that promote binding to MHC class I and a paired increase in cytotoxic pathways, they also involved an increase in pathways that dampen cytotoxic responses (Figure 6C). This contrasts the EBV-negative TME, in which antigen presentation and cytotoxicity were generally dampened.

Given the EBV-associated enhancement of HRS-cell-dependent T cell dysfunction in the cHL TME (Figure 4C), we evaluated T cell exhaustion along the progenitor-terminal continuum.[37] Using established progenitor and terminal exhaustion gene signatures,[56] we found that T cells in EBV-positive TMEs exhibited a stronger terminal exhaustion signature, whereas EBV-negative TMEs showed enrichment of progenitor exhaustion signatures, consistent across both CD8 and CD4 T cell lineages (Figures 6D and S11B). Analysis of antiviral-associated and dysfunction-related gene programs (Figures 6B and 6C) revealed an EBV-linked increase in CD8 T cell antiviral responses and dysfunction signatures (Figure S12), which were consistently observed across multiple cell populations within the cHL TME. Multivariate regression further identified EBV status as the dominant clinical variable associated with terminal T cell exhaustion (Table S8). In addition to exhaustion, EBV-positive TMEs showed increased expression of *KLRG1* and *B3GAT1* (CD57) and decreased *CD28* expression (Figure S11C), consistent with T cell senescence and related dysfunctional states.[57]

To assess the contribution of EBV to T cell dysfunction, we first stratified EBV-positive samples into "EBV-low" and "EBV-high" categories (Figure S11E) based on the expression of EBV transcripts and found a graded increase in T cell terminal exhaustion signatures from EBV-negative to EBV-high (Figure 6E, left). We next correlated the expression of each EBV transcript detected in the tumor population to T cell terminal dysfunction signatures within EBV-positive samples and found robust positive correlations for *LMP1* (Figure 6E, right), one of the main EBV oncogenes,[58] as well as *EBER1* and *EBER2*, the main diagnostic feature of EBV status (Figure S11D). These data support a key role of EBV in promoting T cell dysfunction, potentially through the expression of LMP1.

Although GeoMx provides regional transcriptomic profiling, its limited spatial resolution prompted further analysis using CosMx single-cell spatial transcriptomics,[22] profiling 961 human and 6 EBV genes (Figure 6F) in an independent cohort of 22 EBV-positive and 21 EBV-negative cHL samples (Table S2). This approach identified 12 distinct cell populations (Figure 6G). EBV-positive TMEs were enriched for CD8 T cells and Tregs, whereas EBV-negative TMEs showed higher CD4 T cell proportions (Figure 6H). Tumor-dense regions exhibited elevated T cell dysfunction signatures exclusively in EBV-positive samples (Figure 6I), consistent with MIBI and GeoMx findings and supporting a proximity-dependent role for EBV-positive tumor cells in driving T cell dysfunction (Figure 4C).

We additionally found that *LMP1* expression level on tumor cells is positively correlated with CD4 and CD8 T cell terminal exhaustion (Figure 6J) consistent with our GeoMx data (Figure 6D), leading us to assess if T cell dysfunction is spatially

Figure 3. Cell neighborhood and EBV-linked T cell dysfunction in the cHL TME

(A) Representative annotated cell phenotype maps of EBV-positive and EBV-negative cHL FOVs (left, top), along with the visualization of their corresponding spatial diversity scores (left bottom), with three different spatial diversity index scores quantified (right).

(B) Visualization of the corresponding cellular neighborhoods (CN) for representative tissues (left), along with the proportions of cell phenotypes across the eight identified CNs (right). Abundances of cell types within each CN are in Figure S6B.

(C) Relative proportion of the major immunomodulatory cell types identified in Figure 2F within CN-0 and CN-1, and stratified by EBV status. Two-sided Wilcoxon tests were conducted for all combination of cell phenotype, cell neighborhood, and EBV status; test results were BH-adjusted with a 0.05 FDR. Unadjusted *p* value and BH corrected test results are in Table S8. Significance stars are only shown for significant comparisons. Data for the other cell types in the cHL TME are in Figure S6C.

(D) Relative *Z* score expression level of markers for MHC class I (B2M and HLA-1), MHC class II (HLA-DR), memory T cells (CD45RO), and T cell dysfunction features (Tox, Lag3, PD-1, and PD-L1) on the relevant cell types with the cell counts inscribed above, stratified by CN (columns) and EBV status (rows). Statistical test results for (D) are in Figure S6D. Mean expression visualizations are in Figure S6E. All statistical tests were performed by comparing EBV-positive to EBV-negative populations, and unadjusted *p*-value and BH corrected test results are in Table S4. Significant stars: *$p \leq 0.05$, **$p \leq 0.01$, ***$p \leq 0.001$, ****$p \leq 0.0001$.

CellPress
OPEN ACCESS

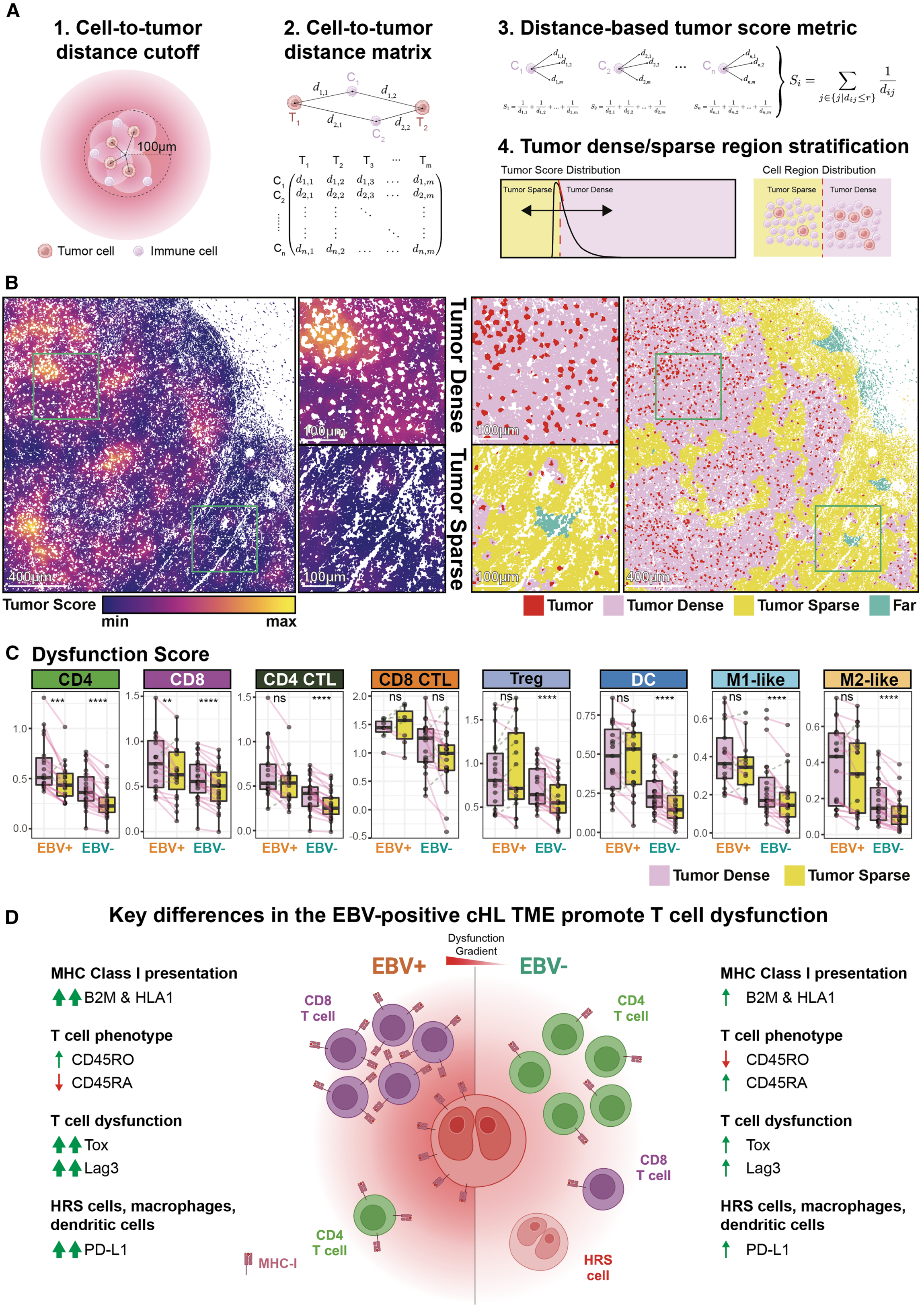

(legend on next page)

organized around LMP1-expressing tumors. We found that both CD4 and CD8 T cells consistently showed highest exhaustion signatures that negatively correlated with increasing distance from tumor cells, but only in LMP1-high regions (Figure 6K). This suggests that EBV-positive HRS cells skew T cell dysfunction from a progenitor-like to a terminal-like exhausted state, a spatially linked process linked to the expression of the LMP1 viral oncogene.

HRS:CD8 T cell interactions promote T cell dysfunction in EBV-positive cHL

The spatial predominance of memory-differentiated, highly dysfunctional CD8 T cells surrounding EBV-positive HRS cells retaining MHC class I expression suggests enhanced CD8: HRS engagement as a mechanism driving dysfunction. To test this, we integrated a proximity ligation assay (PLA) targeting CD8-MHC class I interactions[59] into a CODEX panel incorporating key T cell functional markers (Figures 7A andS13). This CODEX-PLA panel was applied to an independent cHL cohort to identify CD8:HRS interaction pairs *in situ* and relate them to dysfunction states in EBV-positive and EBV-negative disease. Cell annotations were confirmed on CODEX-PLA images (Figures 7B and S15A). Consistent with prior datasets, EBV-positive samples exhibited lower HRS density and higher CD8 T cell density (Figure 7C).

To determine if CD8:HRS engagement elevates T cell dysfunction in EBV-positive cHL, we built a cell network graph[60] to identify the cells immediately adjacent on HRS cells (Figure 7D, left). In EBV-positive samples, we found not only significantly higher proportions of CD8 T cells that are HRS-adjacent (Figure 7D, middle) but also significantly more interaction events between these CD8 T cells with adjacent HRS cells (Figure 7D, right). Further dissection of T cell functional markers revealed that while HRS-adjacent CD8 T cells were consistently more dysfunctional in both EBV-positive and EBV-negative samples (Figure 7E, left), this was markedly greater in EBV-positive cases (Figure 7E, right). This demonstrates that EBV can tune the CD8:HRS interaction axis to exacerbate T cell dysfunction, in support of a more terminal-like exhaustion state we observed across our spatial transcriptomics datasets (Figure 6).

We next evaluated whether LMP1 expression on HRS cells modulates T cell dysfunction in EBV-positive cHL. HRS-adjacent CD8 T cells were assigned a "Dysfunction Score" (Figure 4) and correlated with HRS LMP1 expression. A moderate positive correlation was observed in samples enriched for LMP1-expressing HRS cells but not in LMP1-low samples (Figure 7F, top). CosMx analysis further demonstrated increased differentiation, receptor signaling, and activation pathways, along with reduced PI3K and TOR signaling,[56,61] consistent with terminal exhaustion, in CD8 T cells proximal to LMP1-high HRS cells (Figure 7F, bottom). These data identify LMP1 as a viral driver of CD8 T cell dysfunction in EBV-positive cHL.

Our results support a model (Figure 7G) in which EBV induces HRS cells, in part through the LMP1 viral oncogene, to condition their immediate microenvironment. This includes lower tumor proliferation and altered expression of chemokines and immunomodulatory ligands, for a CD8-heavy and highly dysfunctional T cell landscape. The chronic viral persistence provokes an inflammatory response, including elevated IFN pathways and CD8:HRS engagement through MHC class I, associated with a T cell effector state that is limited in effective tumoricidal programs due to a terminally exhausted functional state.

DISCUSSION

Immunosuppression is a central hallmark of cHL, characterized by a complex immune milieu surrounding malignant HRS cells.[1] Established mechanisms include somatic mutations and genomic alterations in HRS cells that drive frequent downregulation of MHC class I, less frequent loss of MHC class II, and overexpression of the checkpoint ligands PD-L1 and PD-L2.[14,15,27,62,63] Despite these advances, the mechanisms underlying immunosuppression in EBV-linked cHL remain poorly understood, particularly given the lower mutational burden of EBV-positive HRS cells compared to EBV-negative disease.[18,63] Here, we address this gap by defining EBV-associated immune reorganization and suppression within the cHL TME.

Our data is consistent with known features of the cHL TME, including the diversity of immune infiltrates such as lymphocytes, DCs, macrophages, neutrophils, and natural killer cells (Figure 2A), low density of HRS cells and general abundance of T cells (Figure 2A),[64] high expression of PD-L1 on HRS cells and macrophages (Figures 2A and 2E),[27,63,65–67] retention of MHC Class I expression in EBV-positive HRS cells (Figure 2E),[12,13] and a paired increase of PD-1 and PD-L1 (Figures 2F and 3F) that support favorable patient responses to anti-PD-1 blockade.[68]

A key finding of our study is that T cells in EBV-positive cHL are predominantly CD8 and memory-differentiated and exhibit cytotoxic-associated transcripts alongside exhaustion-associated

Figure 4. Immune dysfunction in the cHL TME is a function of spatial proximity to HRS cells

(A) Schematic of our approach to stratify cells into tumor-dense and tumor-sparse populations. (1) Cell-to-tumor distances for non-tumor cells within 100 μm from each tumor cell were (2) organized into a cell-to-tumor distance metric and used to (3) calculate the "tumor score" metric for each non-tumor cell; this "tumor score" metric was used to (4) stratify cells into tumor-sparse and tumor-dense regions based on the global tumor score distribution. The tumor dense/sparse cutoff point and the corresponding cell proportion per FOV in each category are in Figure S9A.

(B) Representative tumor score map (left), along with the visualization of tumor dense and tumor sparse regions (right), in a cHL FOV. Tumor cells are white-masked because they lack a tumor score. Tumor score map and dense/sparse regions for all FOVs are in Figures S7 and S8 respectively. Scale bars: 400 μm (main) and 100 μm (inset).

(C) Dysfunction scores of the major immunomodulatory cell types identified in Figure 2F within tumor dense and tumor spare regions are stratified by EBV status, with each dot representing one FOV. For each tumor-dense and tumor-sparse pair, a one-sided *t* test was performed, with the alternative hypothesis that the mean difference is greater than 0; test results were adjusted for multiple comparisons using the BH method with a targeted FDR at 0.05. The expression of individual markers used to generate the dysfunction score metric is in Figure S9B, and the unadjusted *p* value and BH corrected test results are in Table S4

(D) Cartoon depicting the key differences between EBV-positive and EBV-negative cHL TME. Significant stars: *$p \leq 0.05$, **$p \leq 0.01$, ***$p \leq 0.001$, ****$p \leq 0.0001$.

CellPress
OPEN ACCESS

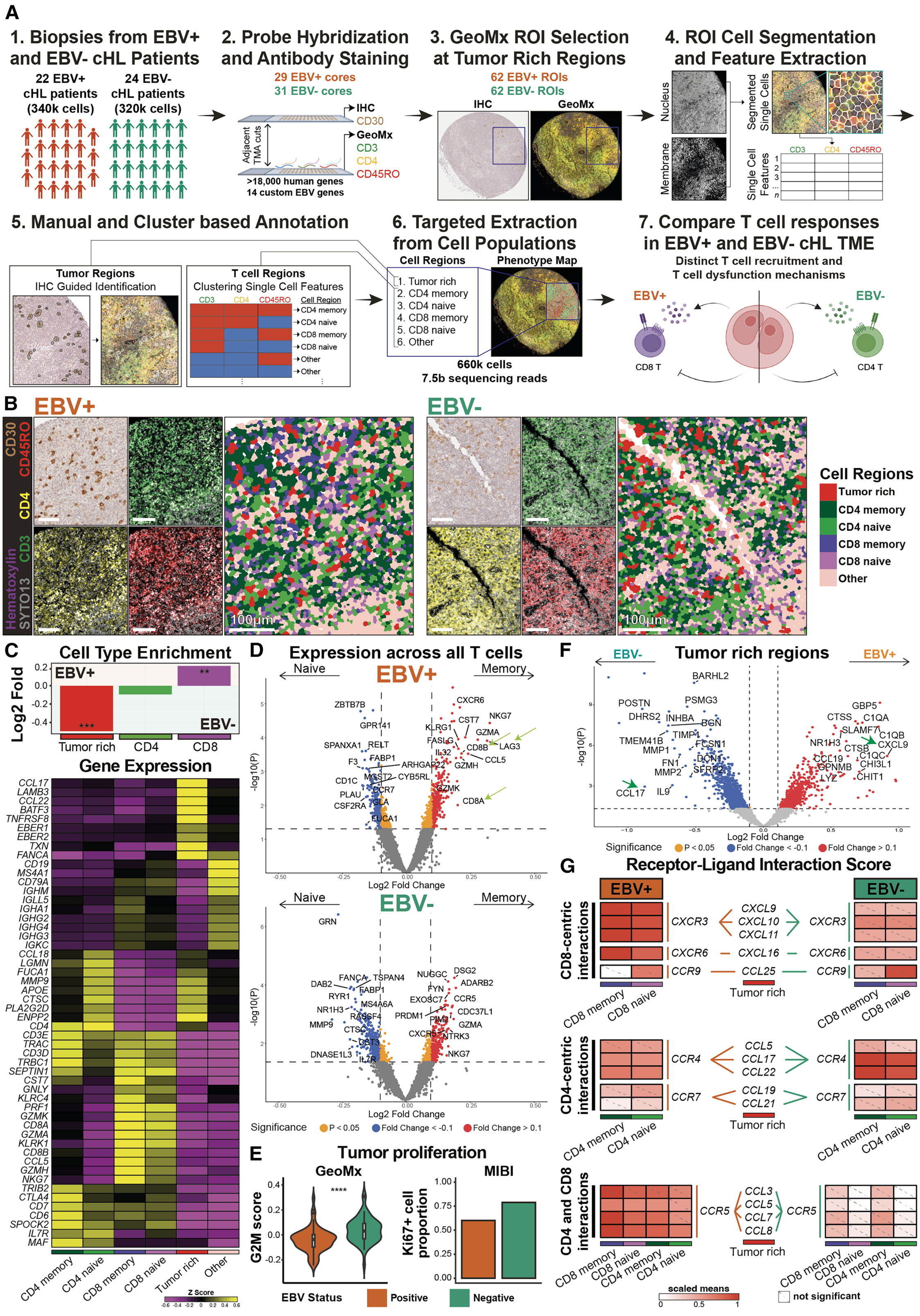

(legend on next page)

signatures, including terminal-exhaustion-like programs. This pattern is consistent with recent reports describing clonally expanded CD8 T cell populations in the cHL TME with combined effector and exhaustion phenotypes.[55,69] Integrating our spatial analyses, we propose a working model in which the accumulation of effector memory CD8 T cells coincides with tissue-level increases in MHC class I expression (HLA1; Figures 2E and 3E), likely reflecting antiviral immune responses driven by IFN signaling (Figure 6B), a potent inducer of MHC class I,[70] and enhanced CD8 T cell chemotaxis into the TME (Figure 5G). In EBV-positive cHL, HRS cells counteract this immunoactivatory state by driving CD8 T cells toward terminal exhaustion in a distance-dependent manner (Figures 2F, 3E, 4D, and 6D), accompanied by increased dysfunction programs and elevated HRS:CD8 T cell engagement (Figure 7F). Supporting this model, antigen-dependent CD8 T cell receptor engagement with MHC class I has been shown to promote terminal exhaustion and limit responsiveness to immune checkpoint blockade in glioblastoma.[71]

Given the paucity of HRS cells, the cHL TME may engage additional immune constituents to sustain T cell interactions and suppress effector responses. DCs and macrophages comprise a substantial fraction of the TME (Figures 2A and 2B) and exhibit distance-dependent PD-L1 upregulation relative to HRS cells (Figures 4C and S9). Although PD-1/PD-L1-mediated suppression by HRS cells and macrophages has been described,[27,28] DC involvement has not, identifying a potential additional contributor to checkpoint blockade response.[68] EBV-associated PD-L1 upregulation in DCs and macrophages (Figures 4C and S9) may relate to increased IFN-γ signaling (Figures 6B and S11A), a known inducer of PD-L1.[72] Elevated PD-L1 expression in EBV-positive HRS cells (Figures 2F and 3E) may reflect both IFN-γ activity[72] (Figures 6B and S11A) and viral oncogene expression, including LMP1 and EBNA2 (Figures 5D and S10B). LMP1 promotes PD-L1 through activation of promoter elements and nuclear factor NF-κB signaling,[43,66] and EBNA2 has been detected in HRS cells,[45] with enhancer binding linked to PD-L1 and PD-L2.[43]

T cell populations in EBV-positive cHL exhibit a terminal-like exhaustion state (Figure 6D). Terminal exhaustion is associated with impaired tumor control and reduced responsiveness to PD-1 blockade compared to progenitor exhaustion and can arise in the setting of chronic viral infection and sustained MHC engagement,[56,71] both observed in EBV-positive cHL (Figures 6 and 7). This is consistent with reports of increased relapse risk in virus-positive cHL.[18] However, T cells in the cHL TME can retain effector capacity upon *ex vivo* restimulation,[55] suggesting incomplete functional impairment. Together, these findings underscore the importance of clinical stratification by viral status in checkpoint blockade responses[18] and implicate LMP1 as a potential therapeutic target in virus-positive cHL (Figures 6 and 7).

Our spatial multi-modal framework integrates discovery via spatial proteomics (Figures 1, 2, 3, and 4) with orthogonal validation and mechanistic analysis using spatial transcriptomics (Figures 5 and 6) and PLA-enhanced spatial proteomics (Figure 7), enabling detailed interrogation of tumor-immune interactions in archival samples. These results refine prior observations and define EBV-associated differences in T cell infiltration and cellular states. This adaptable framework supports broader application to virus-associated diseases and underscores the need for biologically informed stratification of virus-positive and -negative tumors.

Limitations of the study

A key limitation of this work reflects inherent constraints of spatial profiling. Our findings are based on marker-defined phenotypes and gene signature enrichment in archival tissues and lack direct functional validation. Although analyses span

Figure 5. Spatial multi-omics framework reveals EBV-linked modulation of tumor proliferation and cytokine signatures within the cHL TME

(A) Our customized multi-omics workflow to evaluate EBV-positive and EBV-negative cHL TME. (1) Biopsies from two cohorts of EBV-positive (*n* patient = 22, *n* core = 22) and EBV-negative (*n* patient = 24, *n* core = 22) patients were assembled into TMAs, where (2) one section was stained with anti-CD30 to visualize HRS cells through IHC (top) and the adjacent hybridized with a panel of >18,000 RNA probes targeting human and EBV genes and stained with three fluorescent antibodies for GeoMx (bottom). This allowed the (3) selection of 124 tumor-enriched ROIs (*n* = 62 each) and (4) computational identification of single-cell marker expression within to enable the (5) annotation of tumor, T, and immune cell populations and (6) targeted whole-transcriptome capture within each cell region for (7) dissecting the key differences underlying distinct T cell populations and dysregulation between EBV-positive and EBV-negative cHL TME.

(B) Representative IHC and immunofluorescent images of EBV-positive (left) and EBV-negative cHL (right) sections, with markers for HRS (CD30), CD8 T ($CD3^+$ & $CD4^-$), CD4 T ($CD3^+$ & $CD4^+$), memory T (CD45RO), and nuclei (Hematoxylin and SYTO13) shown in the smaller panels and the corresponding cell annotations shown in the larger panels. Scale bars: 100 μm.

(C) Top: log2 fold enrichment plot of annotated cell regions between EBV-positive and EBV-negative cHL ROIs. Significance stars are only shown for significant comparisons. Two-sided Wilcoxon tests were conducted for all cell-type proportions; test results were BH adjusted with a 0.05 FDR. Unadjusted *p* value and BH corrected test results are in Table S4. Bottom: expression heatmap of the key genes associated with each annotated cell region. Expression heatmap of other T cell cytotoxic genes and EBV genes are respectively in Figures S10A and S9B.

(D) Volcano plots showing differences in gene expression between memory (CD45RO+) and naive T cells (CD45RO−) stratified by EBV status, with a few of the most differentially expressed genes indicated. *CD8A*, *CD8B*, and *LAG3* transcripts are indicated by the green arrows. The volcano plot without EBV stratification is in Figure S10C.

(E) Orthogonal validation of G2M cell proliferation transcriptomic signatures between EBV-positive and EBV-negative tumor regions from GeoMx cohort (left) and Ki67+ HRS cell proportion from MIBI cohort (right). Two-sided Wilcoxon test was conducted for the G2M signature; none were performed for the Ki67+ cell proportion as it shows only two discrete values.

(F) Volcano plot of EBV-positive- vs. EBV-negative-tumor-rich regions, with some of the highly differentially expressed host genes shown. *CXCL9* and *CCL17* are indicated by the green arrows.

(G) Receptor-ligand analysis of chemokines that promote T cell recruitment. Significance stars are only shown for significant comparisons. *p* values were generated from one-sided permutation tests, with the alternative hypothesis that the distribution of a given interaction is greater than the null distribution. Significant stars: $^{**}p \leq 0.01$, $^{***}p \leq 0.001$, $^{****}p \leq 0.0001$.

CellPress
OPEN ACCESS

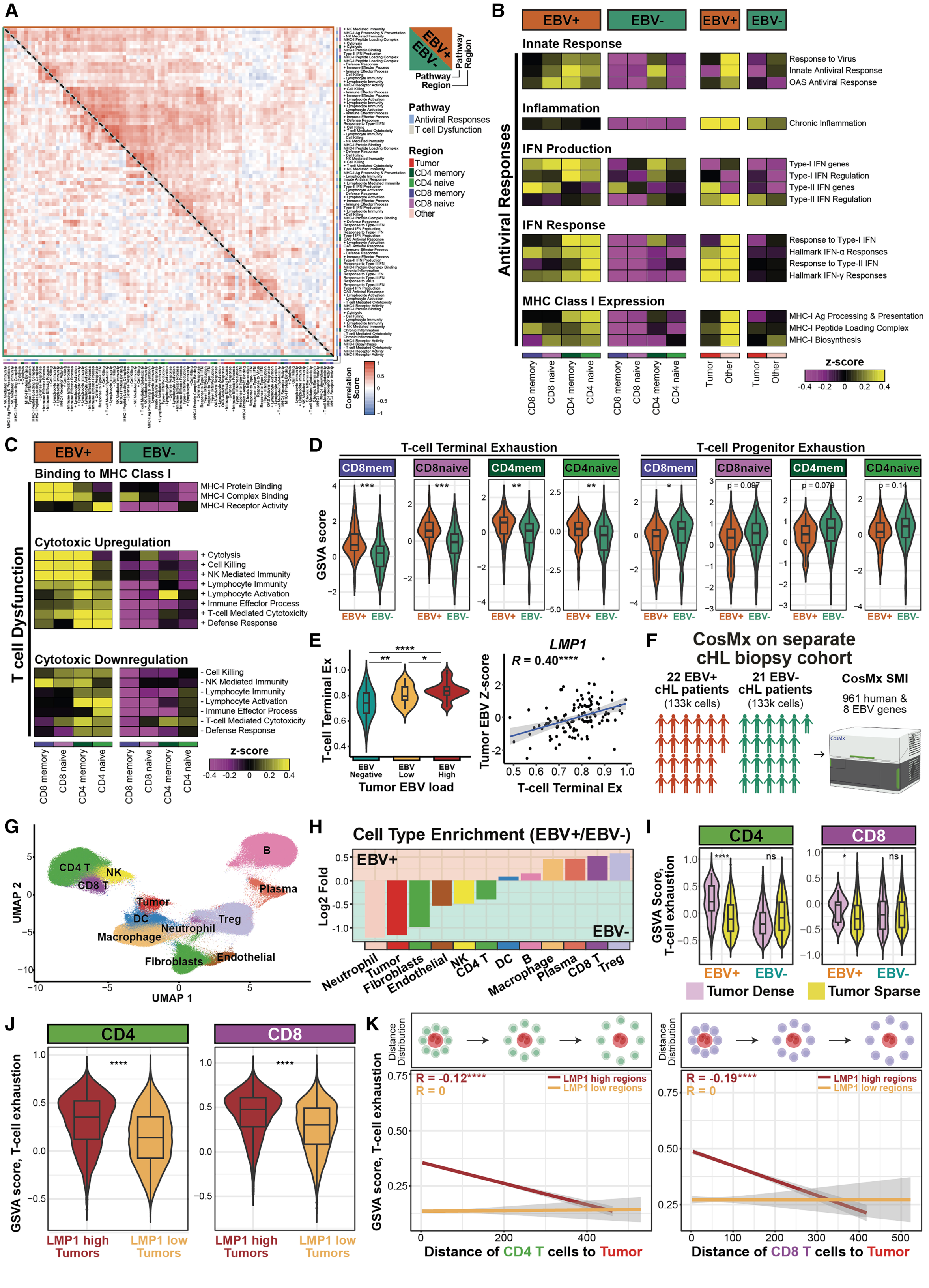

(legend on next page)

increasing spatial resolution, they do not establish causality; for example, increased CD8:MHC-I proximity does not confirm productive T cell receptor signaling despite supportive proteomic and transcriptomic evidence (Figure 7). This limitation stems from the absence of suitable *in vitro* or *in vivo* models linking EBV infection in HRS cells to tissue-level TME reorganization, compounded by the lack of appropriate animal models,[73,74] challenges in generating representative cHL cell lines,[75] and absence of organoid or organ-on-chip systems. Nevertheless, in the absence of established models, emerging spatial technologies provide a valuable framework to interrogate EBV-driven TME remodeling at high resolution.

T cell dysfunction is well characterized in CD8 T cells but less defined in CD4 T cells; our assessment assumes that protein markers (Tox, Lag3, and PD-1) and transcriptional signatures (Figure S11B;[56]) reflect comparable dysfunction states across lineages, despite known molecular differences.[36,37] These dysfunction states are inferred from established markers and transcriptomic programs and lack direct *in vitro* validation using paired patient-derived T cells. Additionally, CD45RO-based stratification in the GeoMx dataset should be interpreted as operational enrichment rather than definitive classification of naive, effector, or memory states. Because GeoMx captures pseudobulk transcriptomes from antibody-defined regions, further subdivision of CD45RO-negative populations into naive versus effector (e.g., Temra) subsets using markers such as CD62L or CCR7 was not feasible, restricting conclusions to region-level comparisons.

Although cHL patients exhibit high response rates to PD-1 blockade (65%–85%), long-term disease-free survival remains lower.[68,76] The relationship between EBV status and outcomes following PD-1 inhibition has not been systematically examined. While our findings suggest a potential association between EBV status and checkpoint response, this study was designed as a discovery analysis of EBV-driven TME reorganization and was not powered to assess clinical outcomes. In addition, cohort sizes were insufficient to evaluate response to checkpoint inhibitors. Future studies should assess overall response rates and long-term outcomes by EBV status, particularly given the higher relapse rates reported in virus-positive cHL.[18]

RESOURCE AVAILABILITY

Lead contact

Further information and requests for resources and reagents should be directed to and will be fulfilled by the lead contact, Sizun Jiang (sjiang3@bidmc.harvard.edu).

Materials availability

No reagents were generated in the study.

Data and code availability

- All proteomics and transcriptomics data, including the images, segmentation masks, and Seurat objects have been deposited at Zenodo. All code necessary to reproduce the analyses have been deposited at Zenodo and also GitHub.
- All data and code are publicly available as of the date of publication. Accession numbers are listed in the key resources table. Any additional information required to reanalyze the data reported in this paper is available from the lead contact upon request.

ACKNOWLEDGMENTS

We thank Matthew Newgren and Maciej Zerkowski (formerly of Ionpath) for technical support; Jim DeCaprio and members of the Shipp, Rodig, and Jiang labs for helpful discussions; Marvin Nayan, Adam Limb, and Mike Chen (NanoString) for GeoMx support; Aniket Gad (Akoya Biosciences) for CODEX support; and Subham Basu, Sara Twist, Felipe Olivera, Magnus Simonsson, and colleagues at Navinci Biosciences for proximity ligation assay support. S.J. is supported in part by NIH DP2AI171139, P01AI177687, and R01AI149672; the Gilead Research Scholars Program in Hematologic Malignancies; a Sanofi Award; the Bill & Melinda Gates Foundation INV-002704; the Dye Family Foundation; and previously the Leukemia Lymphoma Society Career Development Program. S.J.R. is supported by the Bristol-Myers Squibb (BMS) International Immuno-Oncology Network (II-ON). S.J.R. and M.A.S. and a portion of this work were supported by the Blood Cancer Discoveries Grant Program (Leukemia Lymphoma Society, The Mark Foundation, and The Paul G. Allen Frontiers Group). R.C. is supported by NIH/NCI R01 CA196703-01. S.P. and F.I. are supported by Associazione Italiana Ricerca sul Cancro (AIRC) Special 5x1000 Program Metastases (21198). F.M. is funded by R35GM138216 and the Fredrick National Laboratory. G.P.N. holds the Rachford and Carlota A. Harris Endowed Professorship. B.E.G. is funded by NIH CA275301 and CA228700. P.S. is supported by the Lymphoma Research Foundation and a mobility grant from Institut Servier, with institutional research funding from AbbVie and BeiGene. S.P.T.Y. is a Life Science Research Foundation postdoctoral fellow supported by the MacMillan Family Foundation. Y.Y.Y. is a recipient of the Albert J Ryan Fellowship. This article reflects the

Figure 6. Systems-level functional dissection of the cHL TME via multi-scaled spatial transcriptomics

(A) Spearman correlation heatmap of gene set enrichment scores across relevant cell regions between EBV-positive and EBV-negative cHL TME.

(B) Heatmap of gene set enrichment scores for pathways associated with antiviral responses, stratified by EBV status and cell region. Expression of interferons associated with "type-I IFN genes" and "type-II IFN genes" pathways are in Figure S11A.

(C) Heatmap of gene set enrichment scores for pathways associated with T cell dysfunction, stratified by EBV status and cell region.

(D) Comparison of T cell terminal and progenitor exhaustion signatures between EBV-positive and EBV-negative tissues. Expression of genes associated with "terminal exhaustion" and "progenitor exhaustion" are in Figure S11B.

(E) Left: comparison of T cell terminal exhaustion GSVA score between EBV negative, low, and high tumors. Expression of EBV transcripts associated with each group are in Figure S11D. Right: Spearman correlation plots between specific EBV transcripts and T cell terminal exhaustion GSVA score. Correlations for other EBV transcripts are in Figure S11E.

(F) Schematic of CosMx single-cell spatial transcriptomics experiment on a separate cHL cohort with EBV-positive (n patient = 22, n core = 22) and EBV-negative (n patient = 24, n core = 24) samples.

(G) UMAP plot colored by each annotated cell population.

(H) Log2 fold enrichment plot of annotated cell types between EBV-positive and EBV-negative cHL FOVs.

(I) Comparison of CD4 and CD8 T cell exhaustion signatures in tumor dense and sparse regions, stratified by EBV status.

(J) Comparison of CD4 and CD8 T cell exhaustion signatures in EBV-positive samples, stratified by *LMP1* expression.

(K) Spearman correlation of CD4 and CD8 T cell exhaustion signature with the T cell's distance from tumor cells in EBV-positive samples, similarly stratified by *LMP1* expression. Significant stars: *$p \leq 0.05$, **$p \leq 0.01$, ***$p \leq 0.001$, ****$p \leq 0.0001$.

CellPress
OPEN ACCESS

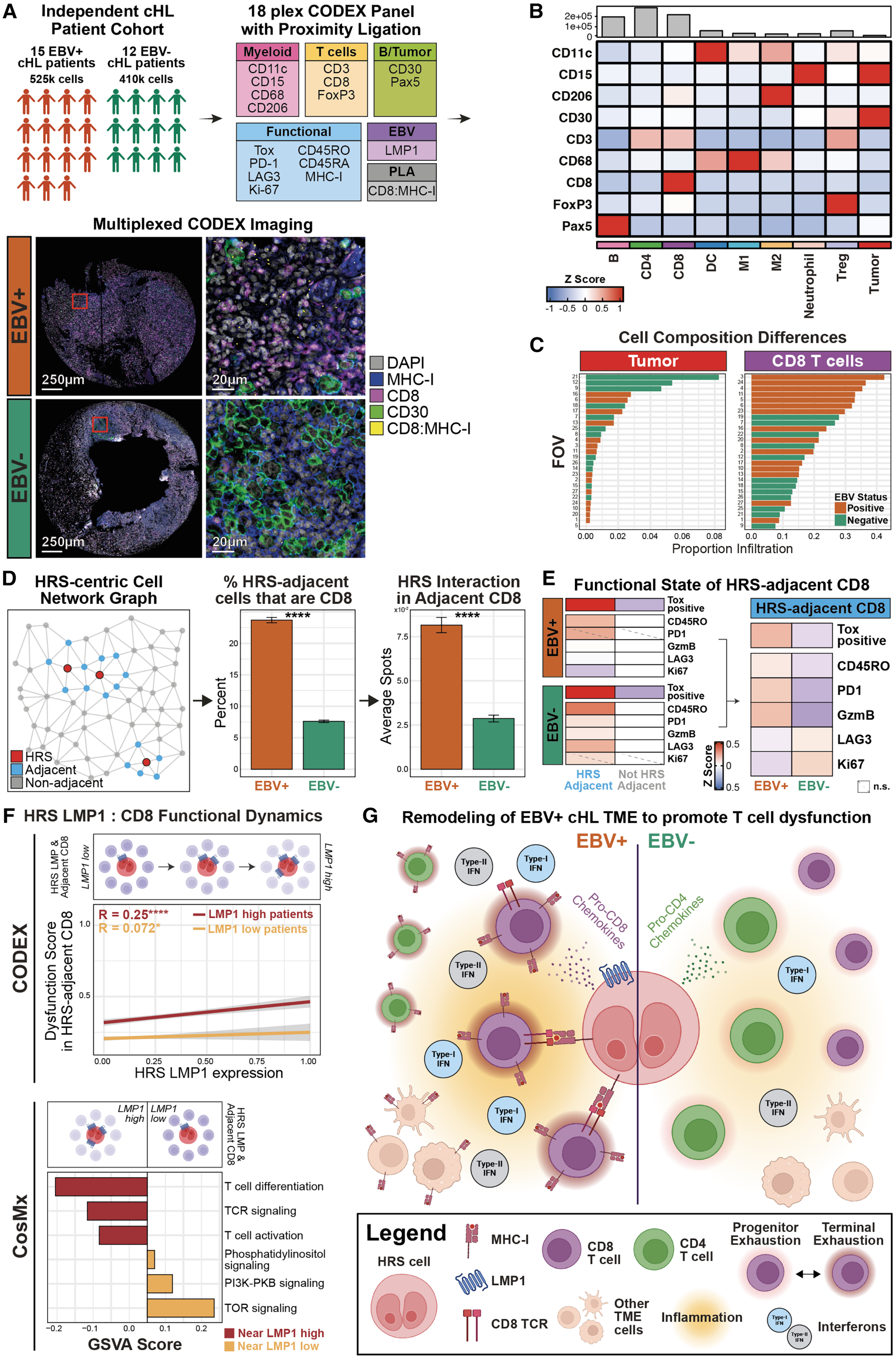

(legend on next page)

views of the authors and does not represent the views or policies of the funding institutions.

AUTHOR CONTRIBUTIONS

S.J., S.J.R., and M.A.S. conceived of the study and planned the experiments with input from all authors. Y.Y.Y., Y.B., S.P.T.Y., H.A.M., K.W., H.C., and S.J. performed experiments. H.Q., Y.Y.Y., B.Z., Y.B., Y.C., F.I., Yuchen Wang, Yang Wang, W.W., J.Y., and S.J. performed data analysis. K.W., M.S., S.S., D.N., V.S., P.R., P.C., J.P., P.S., G.L., A.Y.H., H.W., H.C., L.F., L.K., S.P., B.M., B.E.G., B.Z., G.P.N., B.Z., A.K.S., M.A., C.M.S., F.M., R.C., Q.M., W.R.B., and M.A.S. contributed to the samples, reagents, or pathological and/or technical expertise. Work by G.L., W.W., and Yang Wang was conducted during their tenure as visiting members of the Jiang laboratory. V.S., K.W., P.R., W.R.B., and S.J.R. performed the pathological analysis for the archival human cohort used in this paper. Y.Y.Y., H.Q., Y.B., B.Z, Y.C., F.I., M.A.S., S.J.R., and S.J. wrote the manuscript with input from all authors. S.J. and S.J.R. provided supervision and funding acquisition. All authors reviewed and edited the final manuscript.

DECLARATION OF INTERESTS

S.J. is a co-founder of Elucidate Bio Inc, has received speaking honorariums from Cell Signaling Technology, and has received research support from Roche and Novartis unrelated to this work. S.J.R. has received research support from Affimed, Merck, and BMS, is on the Scientific Advisory Board for Immunitas Therapeutics, and is a part of the BMS II-ON. M.A.S. has received research funding from BMS, Bayer, Abbvie, and AstraZeneca and is on advisory boards for AstraZeneca and BMS. G.P.N. received research grants from Pfizer, Inc.; Vaxart, Inc.; Celgene, Inc.; and Juno Therapeutics, Inc. during the time of and unrelated to this work. C.M.S. is a co-founder and shareholder of Vicinity Bio GmbH and serves on the scientific advisory board of Enable Medicine Inc., which provided research funding unrelated to this work. G.P.N. is a co-founder of Akoya Biosciences, Inc., is an inventor on patent US9909167, and is a Scientific Advisory Board member for Akoya Biosciences, Inc. A.K.S. reports compensation for consulting and/or scientific advisory board membership from Honeycomb Biotechnologies, Cellarity, Ochre Bio, Relation Therapeutics, IntrECate Biotherapeutics, Bio-Rad Laboratories, Fog pharma, Passkey Therapeutics, and Dahlia Biosciences unrelated to this work. R.C. is the founder and consultant of ALKEMIST Bio. R.C. receives research support from Calico Life Sciences LLC.

STAR★METHODS

Detailed methods are provided in the online version of this paper and include the following:

- KEY RESOURCES TABLE
- EXPERIMENTAL MODEL AND STUDY PARTICIPANT DETAILS
- METHOD DETAILS
  - MIBI antibody conjugation
  - Antibody panel titration and validation
  - MIBI gold slide preparation
  - MIBI slides vectabonding
  - MIBI staining
  - MIBI-TOF imaging and image extraction
  - MIBI channel crosstalk removal
  - MIBI image denoising
  - MIBI cell segmentation
  - MIBI image intensity normalization
  - MIBI image to cell expression matrix, REDSEA signal compensation, and across-runs normalization
  - MIBI cell phenotyping
  - GeoMx staining
  - IHC staining
  - GeoMx ROI selection
  - GeoMx cell segmentation
  - GeoMx cell phenotyping
  - GeoMx transcript extraction and sequencing library preparation
  - GeoMx transcript mapping and counting
  - GeoMx data normalization and batch effect removal
  - GeoMx data assessment after batch effect removal
  - CosMx data processing
  - CODEX-PLA antibody conjugation
  - CODEX-PLA antibody staining
  - CODEX-PLA antibody panel imaging
  - CODEX-PLA interaction detection and imaging
  - CODEX-PLA image processing
  - CODEX-PLA cell segmentation
  - CODEX-PLA cell phenotyping
- QUANTIFICATION AND STATISTICAL ANALYSIS
  - MIBI cell level analysis
  - Cell neighborhood calling by spatial LDA
  - Cell phenotype and cell neighborhood enrichment
  - Simpson's diversity index calculation
  - Tumor score and tumor density classification
  - Immune dysfunction level stratified by tumor density and EBV status
  - Bulk RNAseq analysis
  - GeoMx data analysis
  - Receptor-ligand interaction analysis
  - Regression analysis for clinical metadata
  - Analysis related to EBV genes and T cell functional state
  - Circos plots
  - CosMx data analysis
  - Sample stratification based on EBV genes
  - Distance analysis
  - CODEX-PLA cell level analysis
  - Cell network graph

Figure 7. Promotion of CD8 T cell dysfunction in EBV-positive cHL through HRS:T cell interactions and LMP1

(A) CODEX workflow to evaluate CD8 T cell dysfunction state. The bottom panel show representative images of EBV-positive (*n* patient = 15, *n* core = 15) and EBV-negative samples (*n* patient = 12, *n* core = 12), with markers for MHC class I (HLA1), CD8 T cells (CD8), HRS cells (CD30), and CD8:MHC class I interactions. Phenotype maps for all samples are in Figure S14.

(B) Relative *Z* score expression levels of phenotypic markers for the annotated cell phenotypes in this dataset. A mean expression visualization is in Figure S15A.

(C) Relative HRS and CD8 T cell compositions between EBV-positive and EBV-negative samples.

(D) Left: cartoon schematic of the network graph approach used to identify HRS-adjacent cells. Middle: relative proportion of HRS-adjacent cells that are CD8 T cells between EBV-positive and EBV-negative samples (mean ± 1 SE). Right: relative abundance of CD8:HRS cell interactions between EBV-positive and EBV-negative samples (mean ± 1 SE). Two-sided Wilcoxon tests were conducted.

(E) Left: comparison of T cell functional markers between HRS-adjacent and HRS-nonadjacent CD8 T cells within EBV-positive (top) and EBV-negative (bottom) samples. Right: the same comparison, but for HRS-adjacent CD8 T cells between EBV-positive and EBV-negative samples. Tox was binarized into positive/negative expression and shown as percent positive cells. Two-sided Wilcoxon tests were conducted and BH adjusted with a 0.05 FDR.

(F) Top: correlation between LMP1 expression on HRS cells and dysfunction score of HRS-adjacent CD8 T cells in EBV-positive cHL samples that are either abundant (red line) or sparse (yellow line) in LMP1-expressing HRS cells. Images showing the heterogeneity of LMP1 expression on HRS cells are in Figure S15B. Bottom: log2 fold change enrichment of GSVA scores for pathways related to T cell functional states, using the CosMx dataset in Figures 6F–6K.

(G) Proposed mechanism of T cell dysfunction promoted by EBV in the cHL TME. Significant stars: *$p \leq 0.05$, **$p \leq 0.01$, ***$p \leq 0.001$, ****$p \leq 0.0001$.

CellPress
OPEN ACCESS

- Stratification into LMP1 high and LMP1 low samples
- Data visualization

SUPPLEMENTAL INFORMATION

Supplemental information can be found online at https://doi.org/10.1016/j.xcrm.2026.102722.

Received: December 3, 2025
Revised: January 21, 2026
Accepted: March 6, 2026
Published: March 31, 2026

REFERENCES

1. Brice, P., de Kerviler, E., and Friedberg, J.W. (2021). Classical hodgkin lymphoma. Lancet *398*, 1518–1527. https://doi.org/10.1016/S0140-6736(20)32207-8.
2. Alaggio, R., Amador, C., Anagnostopoulos, I., Attygalle, A.D., Araujo, I.B.de O., Berti, E., Bhagat, G., Borges, A.M., Boyer, D., Calaminici, M., et al. (2022). The 5th edition of the world health organization classification of haematolymphoid tumours: lymphoid neoplasms. Leukemia *36*, 1720–1748. https://doi.org/10.1038/s41375-022-01620-2.
3. Weiss, L.M., Movahed, L.A., Warnke, R.A., and Sklar, J. (1989). Detection of epstein–barr viral genomes in reed–sternberg cells of hodgkin's disease. N. Engl. J. Med. *320*, 502–506. https://doi.org/10.1056/nejm198902233200806.
4. Shannon-Lowe, C., Rickinson, A.B., and Bell, A.I. (2017). Epstein–barr virus-associated lymphomas. Philos. Trans. R. Soc. B: Biol. Sci. *372*, 20160271. https://doi.org/10.1098/rstb.2016.0271.
5. Murray, P.G., and Young, L.S. (2019). An etiological role for the epstein-barr virus in the pathogenesis of classical hodgkin lymphoma. Blood *134*, 591–596. https://doi.org/10.1182/blood.2019000568.
6. Teichmann, M., Meyer, B., Beck, A., and Niedobitek, G. (2005). Expression of the interferon-inducible chemokine ip-10 (cxcl10), a chemokine with proposed anti-neoplastic functions, in hodgkin lymphoma and nasopharyngeal carcinoma. J. Pathol. *206*, 68–75. https://doi.org/10.1002/path.1745.
7. Herbst, H., Samol, J., Foss, H.D., Raff, T., and Niedobitek, G. (1997). Modulation of interleukin-6 expression in hodgkin and reed–sternberg cells by epstein–barr virus. J. Pathol. *182*, 299–306. https://doi.org/10.1002/(sici)1096-9896(199707)182:3<299::aid-path856>3.0.co;2-8.
8. Herbst, H., Foss, H.D., Samol, J., Araujo, I., Klotzbach, H., Krause, H., Agathanggelou, A., Niedobitek, G., and Stein, H. (1996). Frequent expression of interleukin-10 by epstein-barr virus-harboring tumor cells of hodgkin's disease. Blood *87*, 2918–2929.
9. Herling, M., Rassidakis, G.Z., Medeiros, L.J., Vassilakopoulos, T.P., Kliche, K.-O., Nadali, G., Viviani, S., Bonfante, V., Giardini, R., Chilosi, M., et al. (2003). Expression of epstein-barr virus latent membrane protein-1 in hodgkin and reed-sternberg cells of classical hodgkin's lymphoma: associations with presenting features, serum interleukin 10 levels, and clinical outcome. Clin. Cancer Res. *9*, 2114–2120.
10. Baumforth, K.R.N., Birgersdotter, A., Reynolds, G.M., Wei, W., Kapatai, G., Flavell, J.R., Kalk, E., Piper, K., Lee, S., Machado, L., et al. (2008). Expression of the epstein-barr virus-encoded epstein-barr virus nuclear antigen 1 in hodgkin's lymphoma cells mediates up-regulation of ccl20 and the migration of regulatory t cells. Am. J. Pathol. *173*, 195–204. https://doi.org/10.2353/ajpath.2008.070845.
11. Briukhovetska, D., Dörr, J., Endres, S., Libby, P., Dinarello, C.A., and Kobold, S. (2021). Interleukins in cancer: from biology to therapy. Nat. Rev. Cancer *21*, 481–499. https://doi.org/10.1038/s41568-021-00363-z.
12. Oudejans, J.J., Jiwa, N.M., Kummer, J.A., Horstman, A., Vos, W., Baak, J.P., Kluin, P.M., van der Valk, P., Walboomers, J.M., and Meijer, C.J. (1996). Analysis of major histocompatibility complex class i expression on reed-sternberg cells in relation to the cytotoxic t-cell response in epstein-barr virus-positive and -negative hodgkin's disease. Blood *87*, 3844–3851.
13. Murray, P.G., Constandinou, C.M., Crocker, J., Young, L.S., and Ambinder, R.F. (1998). Analysis of major histocompatibility complex class i, tap expression, and lmp2 epitope sequence in epstein-barr virus-positive hodgkin's disease. Blood *92*, 2477–2483.
14. Roemer, M.G.M., Advani, R.H., Redd, R.A., Pinkus, G.S., Natkunam, Y., Ligon, A.H., Connelly, C.F., Pak, C.J., Carey, C.D., Daadi, S.E., et al. (2016). Classical hodgkin lymphoma with reduced β2m/mhc class i expression is associated with inferior outcome independent of 9p24.1 status. Cancer Immunol. Res. *4*, 910–916. https://doi.org/10.1158/2326-6066.cir-16-0201.
15. Roemer, M.G.M., Redd, R.A., Cader, F.Z., Pak, C.J., Abdelrahman, S., Ouyang, J., Sasse, S., Younes, A., Fanale, M., Santoro, A., et al. (2018). Major histocompatibility complex class ii and programmed death ligand 1 expression predict outcome after programmed death 1 blockade in classic hodgkin lymphoma. J. Clin. Oncol. *36*, JCO.2017.77.399. https://doi.org/10.1200/jco.2017.77.3994.
16. Lee, S.P., Constandinou, C.M., Thomas, W.A., Croom-Carter, D., Blake, N.W., Murray, P.G., Crocker, J., and Rickinson, A.B. (1998). Antigen presenting phenotype of hodgkin reed-sternberg cells: analysis of the hla class i processing pathway and the effects of interleukin-10 on epstein-barr virus-specific cytotoxic t-cell recognition. Blood *92*, 1020–1030.
17. Wu, R., Sattarzadeh, A., Rutgers, B., Diepstra, A., van den Berg, A., and Visser, L. (2016). The microenvironment of classical hodgkin lymphoma: heterogeneity by epstein–barr virus presence and location within the tumor. Blood Cancer J. *6*, e417. https://doi.org/10.1038/bcj.2016.26.
18. Heger, J.-M., Mammadova, L., Mattlener, J., Sobesky, S., Cirillo, M., Altmüller, J., Kirst, E., Reinke, S., Klapper, W., Bröckelmann, P.J., et al. (2024). Circulating tumor dna sequencing for biologic classification and individualized risk stratification in patients with hodgkin lymphoma. J. Clin. Oncol. *42*, 4218–4230.
19. Angelo, M., Bendall, S.C., Finck, R., Hale, M.B., Hitzman, C., Borowsky, A.D., Levenson, R.M., Lowe, J.B., Liu, S.D., Zhao, S., et al. (2014). Multiplexed ion beam imaging of human breast tumors. Nat. Med. *20*, 436–442. https://doi.org/10.1038/nm.3488.
20. Goltsev, Y., Samusik, N., Kennedy-Darling, J., Bhate, S., Hale, M., Vazquez, G., Black, S., and Nolan, G.P. (2018). Deep profiling of mouse splenic architecture with codex multiplexed imaging. Cell *174*, 968–981.e15.
21. Merritt, C.R., Ong, G.T., Church, S.E., Barker, K., Danaher, P., Geiss, G., Hoang, M., Jung, J., Liang, Y., McKay-Fleisch, J., et al. (2020). Multiplex digital spatial profiling of proteins and rna in fixed tissue. Nat. Biotechnol. *38*, 586–599. https://doi.org/10.1038/s41587-020-0472-9.
22. He, S., Bhatt, R., Brown, C., Brown, E.A., Buhr, D.L., Chantranuvatana, K., Danaher, P., Dunaway, D., Garrison, R.G., Geiss, G., et al. (2022). High-plex imaging of rna and proteins at subcellular resolution in fixed tissue by spatial molecular imaging. Nat. Biotechnol. *40*, 1794–1806.
23. Phillips, D., Schürch, C.M., Khodadoust, M.S., Kim, Y.H., Nolan, G.P., and Jiang, S. (2021). Highly multiplexed phenotyping of immunoregulatory proteins in the tumor microenvironment by codex tissue imaging. Front. Immunol. *12*, 687673. https://doi.org/10.3389/fimmu.2021.687673.
24. Jiang, S., Chan, C.N., Rovira-Clavé, X., Chen, H., Bai, Y., Zhu, B., McCaffrey, E., Greenwald, N.F., Liu, C., Barlow, G.L., et al. (2022). Combined protein and nucleic acid imaging reveals virus-dependent b cell and macrophage immunosuppression of tissue microenvironments. Immunity *55*, 1118–1134.e8. https://doi.org/10.1016/j.immuni.2022.03.020.
25. Wright, K.T., Weirather, J.L., Jiang, S., Kao, K.Z., Sigal, Y., Giobbie-Hurder, A., Shipp, M.A., and Rodig, S.J. (2023). Diffuse large b-cell lymphomas have spatially-defined tumor-immune microenvironments revealed by high-parameter imaging. Blood Adv. *7*, 4633–4646. https://doi.org/10.1182/bloodadvances.2023009813.

26. Bai, Y., Zhu, B., Rovira-Clave, X., Chen, H., Markovic, M., Chan, C.N., Su, T.-H., McIlwain, D.R., Estes, J.D., Keren, L., et al. (2021). Adjacent cell marker lateral spillover compensation and reinforcement for multiplexed images. Front. Immunol. *12*, 652631. https://doi.org/10.3389/fimmu.2021.652631.

27. Roemer, M.G.M., Advani, R.H., Ligon, A.H., Natkunam, Y., Redd, R.A., Homer, H., Connelly, C.F., Sun, H.H., Daadi, S.E., Freeman, G.J., et al. (2016). Pd-l1 and pd-l2 genetic alterations define classical hodgkin lymphoma and predict outcome. J. Clin. Oncol. *34*, 2690–2697. https://doi.org/10.1200/jco.2016.66.4482.

28. Carey, C.D., Gusenleitner, D., Lipschitz, M., Roemer, M.G.M., Stack, E.C., Gjini, E., Hu, X., Redd, R., Freeman, G.J., Neuberg, D., et al. (2017). Topological analysis reveals a pd-l1-associated microenvironmental niche for reed-sternberg cells in hodgkin lymphoma. Blood *130*, 2420–2430. https://doi.org/10.1182/blood-2017-03-770719.

29. Farber, D.L., Yudanin, N.A., and Restifo, N.P. (2014). Human memory t cells: generation, compartmentalization and homeostasis. Nat. Rev. Immunol. *14*, 24–35. https://doi.org/10.1038/nri3567.

30. Alfei, F., Kanev, K., Hofmann, M., Wu, M., Ghoneim, H.E., Roelli, P., Utzschneider, D.T., von Hoesslin, M., Cullen, J.G., Fan, Y., et al. (2019). Tox reinforces the phenotype and longevity of exhausted t cells in chronic viral infection. Nature *571*, 265–269. https://doi.org/10.1038/s41586-019-1326-9.

31. Khan, O., Giles, J.R., McDonald, S., Manne, S., Ngiow, S.F., Patel, K.P., Werner, M.T., Huang, A.C., Alexander, K.A., Wu, J.E., et al. (2019). Tox transcriptionally and epigenetically programs cd8+ t cell exhaustion. Nature *571*, 211–218. https://doi.org/10.1038/s41586-019-1325-x.

32. Scott, A.C., Dündar, F., Zumbo, P., Chandran, S.S., Klebanoff, C.A., Shakiba, M., Trivedi, P., Menocal, L., Appleby, H., Camara, S., et al. (2019). Tox is a critical regulator of tumour-specific t cell differentiation. Nature *571*, 270–274. https://doi.org/10.1038/s41586-019-1324-y.

33. Yao, C., Sun, H.-W., Lacey, N.E., Ji, Y., Moseman, E.A., Shih, H.-Y., Heuston, E.F., Kirby, M., Anderson, S., Cheng, J., et al. (2019). Single-cell rna-seq reveals tox as a key regulator of cd8+ t cell persistence in chronic infection. Nat. Immunol. *20*, 890–901. https://doi.org/10.1038/s41590-019-0403-4.

34. Anderson, A.C., Joller, N., and Kuchroo, V.K. (2016). Lag-3, tim-3, and tigit: Co-inhibitory receptors with specialized functions in immune regulation. Immunity *44*, 989–1004. https://doi.org/10.1016/j.immuni.2016.05.001.

35. Sharpe, A.H., and Pauken, K.E. (2018). The diverse functions of the pd1 inhibitory pathway. Nat. Rev. Immunol. *18*, 153–167. https://doi.org/10.1038/nri.2017.108.

36. Crawford, A., Angelosanto, J.M., Kao, C., Doering, T.A., Odorizzi, P.M., Barnett, B.E., and Wherry, E.J. (2014). Molecular and transcriptional basis of cd4+ t cell dysfunction during chronic infection. Immunity *40*, 289–302. https://doi.org/10.1016/j.immuni.2014.01.005.

37. Wherry, E.J., and Kurachi, M. (2015). Molecular and cellular insights into t cell exhaustion. Nat. Rev. Immunol. *15*, 486–499. https://doi.org/10.1038/nri3862.

38. Cader, F.Z., Schackmann, R.C.J., Hu, X., Wienand, K., Redd, R., Chapuy, B., Ouyang, J., Paul, N., Gjini, E., Lipschitz, M., et al. (2018). Mass cytometry of hodgkin lymphoma reveals a cd4+ regulatory t-cell–rich and exhausted t-effector microenvironment. Blood *132*, 825–836. https://doi.org/10.1182/blood-2018-04-843714.

39. Patel, S.S., Weirather, J.L., Lipschitz, M., Lako, A., Chen, P.-H., Griffin, G.K., Armand, P., Shipp, M.A., and Rodig, S.J. (2019). The microenvironmental niche in classic hodgkin lymphoma is enriched for ctla-4–positive t cells that are pd-1–negative. Blood *134*, 2059–2069. https://doi.org/10.1182/blood.2019002206.

40. Claude, E.S. (1948). A mathematical theory of communication. Bell Syst. Tech. J. *27*, 379–423.

41. Ding, D.Y., Tang, Z., Zhu, B., Ren, H., Shalek, A.K., Tibshirani, R., and Nolan, G.P. (2025). Quantitative characterization of tissue states using multiomics and ecological spatial analysis. Nat. Genet. *57*, 910–921.

42. Chen, Z., Soifer, I., Hilton, H., Keren, L., and Jojic, V. (2020). Modeling multiplexed images with spatial-lda reveals novel tissue microenvironments. J. Comput. Biol. *27*, 1204–1218. https://doi.org/10.1089/cmb.2019.0340.

43. Jiang, S., Zhou, H., Liang, J., Gerdt, C., Wang, C., Ke, L., Schmidt, S.C.S., Narita, Y., Ma, Y., Wang, S., et al. (2017). The epstein-barr virus regulome in lymphoblastoid cells. Cell Host Microbe *22*, 561–573.e4. https://doi.org/10.1016/j.chom.2017.09.001.

44. Sallusto, F., Geginat, J., and Lanzavecchia, A. (2004). Central memory and effector memory t cell subsets: function, generation, and maintenance. Annu. Rev. Immunol. *22*, 745–763. https://doi.org/10.1146/annurev.immunol.22.012703.104702.

45. Bayda, N., Tilloy, V., Chaunavel, A., Bahri, R., Halabi, M.A., Feuillard, J., Jaccard, A., and Ranger-Rogez, S. (2021). Comprehensive epstein-barr virus transcriptome by rna-sequencing in angioimmunoblastic t cell lymphoma (aitl) and other lymphomas. Cancers *13*, 610. https://doi.org/10.3390/cancers13040610.

46. Kohli, K., Pillarisetty, V.G., and Kim, T.S. (2022). Key chemokines direct migration of immune cells in solid tumors. Cancer Gene Ther. *29*, 10–21. https://doi.org/10.1038/s41417-021-00303-x.

47. Gunn, M.D., Tangemann, K., Tam, C., Cyster, J.G., Rosen, S.D., and Williams, L.T. (1998). A chemokine expressed in lymphoid high endothelial venules promotes the adhesion and chemotaxis of naive t lymphocytes. Proc. Natl. Acad. Sci. *95*, 258–263. https://doi.org/10.1073/pnas.95.1.258.

48. Campbell, J.J., O'Connell, D.J., and Wurbel, M.-A. (2007). Cutting edge: Chemokine receptor ccr4 is necessary for antigen-driven cutaneous accumulation of cd4 t cells under physiological conditions. J. Immunol. *178*, 3358–3362. https://doi.org/10.4049/jimmunol.178.6.3358.

49. Efremova, M., Vento-Tormo, M., Teichmann, S.A., and Vento-Tormo, R. (2020). Cellphonedb: inferring cell–cell communication from combined expression of multi-subunit ligand–receptor complexes. Nat. Protoc. *15*, 1484–1506. https://doi.org/10.1038/s41596-020-0292-x.

50. Troulé, K., Petryszak, R., Cakir, B., Cranley, J., Harasty, A., Prete, M., Tuong, Z., Teichmann, S.A., Garcia-Alonso, L., and Vento-Tormo, R. (2023). CellPhoneDB v5: inferring cell–cell communication from single-cell multiomics data. Nat. Protoc. *20*, 3412–3440. https://doi.org/10.1038/s41596-024-01137-1.

51. Di Pilato, M., Kfuri-Rubens, R., Pruessmann, J.N., Ozga, A.J., Messemaker, M., Cadilha, B.L., Sivakumar, R., Cianciaruso, C., Warner, R.D., Marangoni, F., et al. (2021). Cxcr6 positions cytotoxic t cells to receive critical survival signals in the tumor microenvironment. Cell *184*, 4512–4530.e22. https://doi.org/10.1016/j.cell.2021.07.015.

52. Lee, B., Sharron, M., Montaner, L.J., Weissman, D., and Doms, R.W. (1999). Quantification of cd4, ccr5, and cxcr4 levels on lymphocyte subsets, dendritic cells, and differentially conditioned monocyte-derived macrophages. Proc. Natl. Acad. Sci. *96*, 5215–5220. https://doi.org/10.1073/pnas.96.9.5215.

53. Jiao, X., Nawab, O., Patel, T., Kossenkov, A.V., Halama, N., Jaeger, D., and Pestell, R.G. (2019). Recent advances targeting ccr5 for cancer and its role in immuno-oncology. Cancer Res. *79*, 4801–4807. https://doi.org/10.1158/0008-5472.can-19-1167.

54. Hansen, T.H., and Bouvier, M. (2009). Mhc class i antigen presentation: learning from viral evasion strategies. Nat. Rev. Immunol. *9*, 503–513. https://doi.org/10.1038/nri2575.

55. Chen, X., Yu, J., Venkataraman, G., Smith, S.M., Chen, M., Cooper, A., Tumuluru, S., Brody, J.D., Godfrey, J., and Kline, J. (2024). T cell states, repertoire and function in classical hodgkin lymphoma revealed through single-cell analyses. Cancer Immunol. Res. *12*, 296–307. https://doi.org/10.1158/2326-6066.cir-23-0547.

56. Miller, B.C., Sen, D.R., Al Abosy, R., Bi, K., Virkud, Y.V., LaFleur, M.W., Yates, K.B., Lako, A., Felt, K., Naik, G.S., et al. (2019). Subsets of exhausted cd8+ t cells differentially mediate tumor control and respond to checkpoint blockade. Nat. Immunol. *20*, 326–336. https://doi.org/10.1038/s41590-019-0312-6.

57. Crespo, J., Sun, H., Welling, T.H., Tian, Z., and Zou, W. (2013). T cell anergy, exhaustion, senescence, and stemness in the tumor microenvironment. Curr. Opin. Immunol. *25*, 214–221. https://doi.org/10.1016/j.coi.2012.12.003.

58. Wang, L.W., Jiang, S., and Gewurz, B.E. (2017). Epstein-barr virus lmp1-mediated oncogenicity. J. Virol. *91*, e01718-16.

59. Fredriksson, S., Gullberg, M., Jarvius, J., Olsson, C., Pietras, K., Gústafsdóttir, S.M., Östman, A., and Landegren, U. (2002). Protein detection using proximity-dependent dna ligation assays. Nat. Biotechnol. *20*, 473–477.

60. Yeo, Y.Y., Chang, Y., Qiu, H., Yiu, S.P.T., Michel, H.A., Wu, W., Jin, X., Kure, S., Parmelee, L., Luo, S., et al. (2024). Same-slide spatial multiomics integration reveals tumor virus-linked spatial reorganization of the tumor microenvironment. bioRxiv. https://doi.org/10.1101/2024.12.20.629650.

61. Franco, F., Jaccard, A., Romero, P., Yu, Y.-R., and Ho, P.-C. (2020). Metabolic and epigenetic regulation of t-cell exhaustion. Nat. Metab. *2*, 1001–1012.

62. Steidl, C., Shah, S.P., Woolcock, B.W., Rui, L., Kawahara, M., Farinha, P., Johnson, N.A., Zhao, Y., Telenius, A., Neriah, S.B., et al. (2011). Mhc class ii transactivator ciita is a recurrent gene fusion partner in lymphoid cancers. Nature *471*, 377–381. https://doi.org/10.1038/nature09754.

63. Wienand, K., Chapuy, B., Stewart, C., Dunford, A.J., Wu, D., Kim, J., Kamburov, A., Wood, T.R., Cader, F.Z., Ducar, M.D., et al. (2019). Genomic analyses of flow-sorted hodgkin reed-sternberg cells reveal complementary mechanisms of immune evasion. Blood Adv. *3*, 4065–4080. https://doi.org/10.1182/bloodadvances.2019001012.

64. Poppema, S., Bhan, A.K., Reinherz, E.L., Posner, M.R., and Schlossman, S.F. (1982). In situ immunologic characterization of cellular constituents in lymph nodes and spleens involved by hodgkin's disease. Blood *59*, 226–232.

65. Green, M.R., Monti, S., Rodig, S.J., Juszczynski, P., Currie, T., O'Donnell, E., Chapuy, B., Takeyama, K., Neuberg, D., Golub, T.R., et al. (2010). Integrative analysis reveals selective 9p24.1 amplification, increased pd-1 ligand expression, and further induction via jak2 in nodular sclerosing hodgkin lymphoma and primary mediastinal large b-cell lymphoma. Blood *116*, 3268–3277. https://doi.org/10.1182/blood-2010-05-282780.

66. Green, M.R., Rodig, S., Juszczynski, P., Ouyang, J., Sinha, P., O'Donnell, E., Neuberg, D., and Shipp, M.A. (2012). Constitutive ap-1 activity and ebv infection induce pd-l1 in hodgkin lymphomas and posttransplant lymphoproliferative disorders: Implications for targeted therapy. Clin. Cancer Res. *18*, 1611–1618. https://doi.org/10.1158/1078-0432.ccr-11-1942.

67. Chen, B.J., Chapuy, B., Ouyang, J., Sun, H.H., Roemer, M.G.M., Xu, M.L., Yu, H., Fletcher, C.D.M., Freeman, G.J., Shipp, M.A., and Rodig, S.J. (2013). Pd-l1 expression is characteristic of a subset of aggressive b-cell lymphomas and virus-associated malignancies. Clin. Cancer Res. *19*, 3462–3473. https://doi.org/10.1158/1078-0432.ccr-13-0855.

68. Ansell, S.M., Lesokhin, A.M., Borrello, I., Halwani, A., Scott, E.C., Gutierrez, M., Schuster, S.J., Millenson, M.M., Cattry, D., Freeman, G.J., et al. (2015). Pd-1 blockade with nivolumab in relapsed or refractory hodgkin's lymphoma. N. Engl. J. Med. *372*, 311–319. https://doi.org/10.1056/nejmoa1411087.

69. Müller-Meinhard, B., Seifert, N., Grund, J., Reinke, S., Yalcin, F., Kaul, H., Borchmann, S., von Tresckow, B., Borchmann, P., Plütschow, A., et al. (2024). Human leukocyte antigen (hla) class i expression on hodgkin–reed–sternberg cells is an ebv-independent major determinant of microenvironment composition in classic hodgkin lymphoma. HemaSphere *8*, e84.

70. Zhou, F. (2009). Molecular mechanisms of ifn-γ to up-regulate mhc class i antigen processing and presentation. Int. Rev. Immunol. *28*, 239–260. https://doi.org/10.1080/08830180902978120.

71. Waibl Polania, J., Hoyt-Miggelbrink, A., Tomaszewski, W.H., Wachsmuth, L.P., Lorrey, S.J., Wilkinson, D.S., Lerner, E., Woroniecka, K., Finlay, J.B., Ayasoufi, K., et al. (2024). Antigen presentation by tumor-associated macrophages drives t cells from a progenitor exhaustion state to terminal exhaustion. Immunity.

72. Garcia-Diaz, A., Shin, D.S., Moreno, B.H., Saco, J., Escuin-Ordinas, H., Rodriguez, G.A., Zaretsky, J.M., Sun, L., Hugo, W., Wang, X., et al. (2017). Interferon receptor signaling pathways regulating pd-l1 and pd-l2 expression. Cell Rep. *19*, 1189–1201. https://doi.org/10.1016/j.celrep.2017.04.031.

73. Morgan, R.A. (2012). Human tumor xenografts: The good, the bad, and the ugly. Mol. Ther. *20*, 882–884. https://doi.org/10.1038/mt.2012.73.

74. M'kacher, R., Frenzel, M., Al Jawhari, M., Junker, S., Cuceu, C., Morat, L., Bauchet, A.-L., Stimmer, L., Lenain, A., Dechamps, N., et al. (2018). Establishment and characterization of a reliable xenograft model of hodgkin lymphoma suitable for the study of tumor origin and the design of new therapies. Cancers *10*, 414. https://doi.org/10.3390/cancers10110414.

75. Drexler, H.G., Pommerenke, C., Eberth, S., and Nagel, S. (2018). Hodgkin lymphoma cell lines: to separate the wheat from the chaff. Biol. Chem. *399*, 511–523. https://doi.org/10.1515/hsz-2017-0321.

76. Herrera, A.F., LeBlanc, M., Castellino, S.M., Li, H., Rutherford, S.C., Evens, A.M., Davison, K., Punnett, A., Parsons, S.K., Ahmed, S., et al. (2024). Nivolumab+ avd in advanced-stage classic hodgkin's lymphoma. N. Engl. J. Med. *391*, 1379–1389.

77. Han, G., Spitzer, M.H., Bendall, S.C., Fantl, W.J., and Nolan, G.P. (2018). Metal-isotope-tagged monoclonal antibodies for high-dimensional mass cytometry. Nat. Protoc. *13*, 2121–2148. https://doi.org/10.1038/s41596-018-0016-7.

78. Ji, A.L., Rubin, A.J., Thrane, K., Jiang, S., Reynolds, D.L., Meyers, R.M., Guo, M.G., George, B.M., Mollbrink, A., Bergenstråhle, J., et al. (2020). Multimodal analysis of composition and spatial architecture in human squamous cell carcinoma. Cell *182*, 497–514.e22. https://doi.org/10.1016/j.cell.2020.05.039.

79. Hickey, J.W., Neumann, E.K., Radtke, A.J., Camarillo, J.M., Beuschel, R.T., Albanese, A., McDonough, E., Hatler, J., Wiblin, A.E., Fisher, J., et al. (2022). Spatial mapping of protein composition and tissue organization: a primer for multiplexed antibody-based imaging. Nat. Methods *19*, 284–295. https://doi.org/10.1038/s41592-021-01316-y.

80. Liu, C.C., Bosse, M., Kong, A., Kagel, A., Kinders, R., Hewitt, S.M., Varma, S., van de Rijn, M., Nowak, S.H., Bendall, S.C., and Angelo, M. (2022). Reproducible, high-dimensional imaging in archival human tissue by multiplexed ion beam imaging by time-of-flight (mibi-tof). Lab. Invest. *102*, 762–770. https://doi.org/10.1038/s41374-022-00778-8.

81. Yeo, Y.Y., Cramer, P., Deisher, A., Bai, Y., Zhu, B., Yeo, W.-J., Shipp, M.A., Rodig, S.J., and Jiang, S. (2024). A hitchhiker's guide to high-dimensional tissue imaging with multiplexed ion beam imaging. Methods Cell Biol. *186*, 213–231.

82. Keren, L., Bosse, M., Marquez, D., Angoshtari, R., Jain, S., Varma, S., Yang, S.-R., Kurian, A., Van Valen, D., West, R., et al. (2018). A structured tumor-immune microenvironment in triple negative breast cancer revealed by multiplexed ion beam imaging. Cell *174*, 1373–1387.e19. https://doi.org/10.1016/j.cell.2018.08.039.

83. Keren, L., Bosse, M., Thompson, S., Risom, T., Vijayaragavan, K., McCaffrey, E., Marquez, D., Angoshtari, R., Greenwald, N.F., Fienberg, H., et al. (2019). Mibi-tof: A multiplexed imaging platform relates cellular phenotypes and tissue structure. Sci. Adv. *5*, eaax5851. https://doi.org/10.1126/sciadv.aax5851.

84. Rovira-Clavé, X., Drainas, A.P., Jiang, S., Bai, Y., Baron, M., Zhu, B., Dallas, A., Lee, M., Chu, T., Holzem, A., et al. (2022). Spatial epitope barcoding reveals subclonal tumor patch behaviors. Cancer Cell *40*, 1423–1439. https://doi.org/10.1016/j.ccell.2022.09.014.

85. Ronneberger, O., Fischer, P., and Brox, T. (2025). U-net: Convolutional networks for biomedical image segmentation. In International Conference on Medical image computing and computer-assisted intervention (Cham: Springer international publishing), pp. 234–241. https://doi.org/10.1007/978-3-319-24574.

86. Baranski, A., Milo, I., Greenbaum, S., Oliveria, J.-P., Mrdjen, D., Angelo, M., and Keren, L. (2021). Maui (mbi analysis user interface)—an image processing pipeline for multiplexed mass based imaging. PLoS Comput. Biol. *17*, e1008887. https://doi.org/10.1371/journal.pcbi.1008887.

87. Van Valen, D.A., Kudo, T., Lane, K.M., Macklin, D.N., Quach, N.T., DeFelice, M.M., Maayan, I., Tanouchi, Y., Ashley, E.A., and Covert, M.W. (2016). Deep learning automates the quantitative analysis of individual cells in live-cell imaging experiments. PLoS Comput. Biol. *12*, e1005177. https://doi.org/10.1371/journal.pcbi.1005177.

88. Greenwald, N.F., Miller, G., Moen, E., Kong, A., Kagel, A., Dougherty, T., Fullaway, C.C., McIntosh, B.J., Leow, K.X., Schwartz, M.S., et al. (2022). Whole-cell segmentation of tissue images with human-level performance using large-scale data annotation and deep learning. Nat. Biotechnol. *40*, 555–565. https://doi.org/10.1038/s41587-021-01094-0.

89. Shaban, M., Bai, Y., Qiu, H., Mao, S., Yeung, J., Yeo, Y.Y., Shanmugam, V., Chen, H., Zhu, B., Weirather, J., et al. (2024). MAPS: Pathologist-level cell type annotation from tissue images through machine learning. Nat. Commun. *15*, 28. https://doi.org/10.1038/s41467-023-44188-w.

90. Van Gassen, S., Callebaut, B., Van Helden, M.J., Lambrecht, B.N., Demeester, P., Dhaene, T., and Saeys, Y. (2015). Flowsom: Using self-organizing maps for visualization and interpretation of cytometry data. Cytometry. A. *87*, 636–645. https://doi.org/10.1002/cyto.a.22625.

91. Schiemann, R., Gherardini, P.F., Kageyama, R., Travers, M., and Kitch, L. (2020). Mantis viewer (v1.2.0-beta.1). Zenodo. https://doi.org/10.5281/zenodo.4909620.

92. Levine, J.H., Simonds, E.F., Bendall, S.C., Davis, K.L., Amir, E.a.D., Tadmor, M.D., Litvin, O., Fienberg, H.G., Jager, A., Zunder, E.R., et al. (2015). Data-driven phenotypic dissection of aml reveals progenitor-like cells that correlate with prognosis. Cell *162*, 184–197. https://doi.org/10.1016/j.cell.2015.05.047.

93. Liu, N., Bhuva, D.D., Mohamed, A., Bokelund, M., Kulasinghe, A., Tan, C.W., and Davis, M.J. (2024). standr: spatial transcriptomic analysis for geomx dsp data. Nucleic Acids Res. *52*, e2. https://doi.org/10.1093/nar/gkad1026.

94. Büttner, M., Miao, Z., Wolf, F.A., Teichmann, S.A., and Theis, F.J. (2019). A test metric for assessing single-cell rna-seq batch correction. Nat. Methods *16*, 43–49. https://doi.org/10.1038/s41592-018-0254-1.

95. Li, B., Zhang, W., Guo, C., Xu, H., Li, L., Fang, M., Hu, Y., Zhang, X., Yao, X., Tang, M., et al. (2022). Benchmarking spatial and single-cell transcriptomics integration methods for transcript distribution prediction and cell type deconvolution. Nat. Methods *19*, 662–670. https://doi.org/10.1038/s41592-022-01480-9.

96. Jones, D.C., Elz, A.E., Hadadianpour, A., Ryu, H., Glass, D.R., and Newell, E.W. (2025). Cell simulation as cell segmentation. Nat. Methods *22*, 1331–1342. https://doi.org/10.1038/s41592-025-02697-0.

97. Korsunsky, I., Millard, N., Fan, J., Slowikowski, K., Zhang, F., Wei, K., Baglaenko, Y., Brenner, M., Loh, P.-ru, and Raychaudhuri, S. (2019). Fast, sensitive and accurate integration of single-cell data with harmony. Nat. Methods *16*, 1289–1296.

98. Black, S., Phillips, D., Hickey, J.W., Kennedy-Darling, J., Venkataraaman, V.G., Samusik, N., Goltsev, Y., Schürch, C.M., and Nolan, G.P. (2021). Codex multiplexed tissue imaging with dna-conjugated antibodies. Nat. Protoc. *16*, 3802–3835.

99. Deisher, A., Yeo, Y.Y., and Jiang, S. (2022). Combined protein and nucleic acid staining in tissues with panini. STAR Protoc. *3*, 101663.

100. Wen-Hsiang, T. (1985). Moment-preserving thresolding: A new approach. Comput. Vis. Graph Image Process *29*, 377–393.

101. Yen, J.C., Chang, F.J., and Chang, S. (1995). A new criterion for automatic multilevel thresholding. IEEE Trans. Image Process. *4*, 370–378.

102. Mitra, B., Beri, N.R., Guo, R., Burton, E.M., Murray-Nerger, L.A., and Gewurz, B.E. (2023). Characterization of target gene regulation by the two epstein-barr virus oncogene lmp1 domains essential for b-cell transformation. mBio *14*, e0233823.

103. Gu, Z., Gu, L., Eils, R., Schlesner, M., and Brors, B. (2014). "circlize" implements and enhances circular visualization in r. Bioinformatics *30*, 2811–2812.

104. Niu, Z., O'Farrell, A., Li, J., Reffsin, S., Jain, N., Dardani, I., Goyal, Y., and Raj, A. (2025). Piscis: A loss estimator of the F1 score enables accurate spot detection in fluorescence microscopy images via deep learning. Cell Syst. *16*. https://doi.org/10.1016/j.cels.2025.101448.

105. Gu, Z., Eils, R., and Schlesner, M. (2016). Complex heatmaps reveal patterns and correlations in multidimensional genomic data. Bioinformatics *32*, 2847–2849.

CellPress
OPEN ACCESS

STAR★METHODS

KEY RESOURCES TABLE

REAGENT or RESOURCE	SOURCE	IDENTIFIER
Antibodies		
CD20 (Clone rIGEL/773)	Novus Bio	Clone ID: rIGEL/773<br>Cat #: NBP2-53190
CD163 (Clone EDHu-1)	Novus Bio	Clone ID: EDHu-1<br>Cat #: NB110-40686
Histone H3 (Clone D1H2)	CST	Clone ID: D1H2<br>Cat #: 4499
CD45RO (Clone UCHL1)	BioLegend	Clone ID: UCHL1<br>Cat #: 304202
CD153 (CD30L) (Polyclonal)	R&D	Clone ID: Polyclonal<br>Cat #: AF1028
Lag3 (Clone 17B4)	Novus Bio	Clone ID: 17B4<br>Cat #: NBP1-97657
CD4 (Clone EPR6855)	Abcam	Clone ID: EPR6855<br>Cat #: ab181724
CD11c (Clone EP1347Y)	Abcam	Clone ID: EP1347Y<br>Cat #: ab216655
CD56 (Clone MRQ-42)	Cell Marque	Clone ID: MRQ-42<br>Cat #: 156R
FoxP3 (Clone 236A/E7)	Abcam	Clone ID: 236A/E7<br>Cat #: ab96048
GATA3 (Clone L50-823)	Cell Marque	Clone ID: L50-823<br>Cat #: 390M
Granzyme B (Clone EPR20129-127)	Abcam	Clone ID: EPR20129-127<br>Cat #: ab219803
PD-L1 (Clone E1L3N)	CST	Clone ID: E1L3N<br>Cat #: 13684
CD16 (Clone SP175)	Abcam	Clone ID: SP175<br>Cat #: ab243925
Ki-67 (Clone 8D5)	Novus Bio	Clone ID: 8D5<br>Cat #: NBP2-22112
PD-1 (Clone D4W2J)	CST	Clone ID: D4W2J<br>Cat #: 86163S
PD-1 (Clone NAT105)	Abcam	Clone ID: NAT105<br>Cat #: ab201811
Pax-5 (Clone D7H5X)	CST	Clone ID: D7H5X<br>Cat #: 93009
Tox/Tox2 (Clone E6I3Q)	CST	Clone ID: E6I3Q<br>Cat #: 73758
CD161 (Polyclonal)	Abcam	Clone ID: Polyclonal<br>Cat #: ab197979
CD68 (Clone D4B9C)	CST	Clone ID: D4B9C<br>Cat #: 26042
B2-Microglobulin (Clone D8P1H)	CST	Clone ID: D8P1H<br>Cat #: 12851
CD8 (Clone C8/144B)	Cell Marque	Clone ID: C8/144B<br>Cat #: 108M
CD3 (Clone MRQ-39)	Cell Marque	Clone ID: MRQ-39<br>Cat #: 103R

(*Continued on next page*)

Continued		
REAGENT or RESOURCE	SOURCE	IDENTIFIER
HLA1 (Clone EMR8-5)	Abcam	Clone ID: EMR8-5<br>Cat #: ab70328
CD15 (Clone MC480)	BioLegend	Clone ID: MC480<br>Cat #: 125602
T-bet (Clone 4B10)	BioLegend	Clone ID: 4B10<br>Cat #: 644802
CD14 (Clone SP192)	Abcam	Clone ID: SP192<br>Cat #: ab230903
CD45RA (Clone HI100)	BioLegend	Clone ID: HI100<br>Cat #: 304102
HLA-DR (Clone EPR3692)	Abcam	Clone ID: EPR3692<br>Cat #: ab92511
CD57 (Clone HNK-1)	BioLegend	Clone ID: HNK-1<br>Cat #: 359602
CD30 (Clone Ber-H2)	Cell Marque	Clone ID: Ber-H2<br>Cat #: 130M
Na-K ATPase (Clone EP1845Y)	Abcam	Clone ID: EP1845Y<br>Cat #: ab167390
CD3 (Clone D7A6E)	CST	Clone ID: D7A6E<br>Cat #: 85061
CD4 (Clone EPR6855)	Abcam	Clone ID: EPR6855<br>Cat #: ab181724
anti-mouse IgG (Polyclonal)	Invitrogen	Clone ID: Polyclonal<br>Cat #: A31571
CD30 (Clone Ber-H2)	Santa Cruz Biotech	Clone ID: Ber-H2<br>Cat #: sc-19658
CD30 (Clone Ber-H2)	CST	Clone ID: E4L4I<br>Cat #: 47448
LMP1 (Clone CS1-4)	Abcam	Clone ID: CS1-4<br>Cat #: ab78113
CD8 (Clone D8A8Y)	CST	Clone ID: D8A8Y<br>Cat #: 85336
CD15 (Clone MMA)	BioLegend	Clone ID: MMA<br>Cat #: 394702
CD206 (Clone E6T5J)	CST	Clone ID: E6T5J<br>Cat #: 87887
Lag3 (Clone D2G4O)	CST	Clone ID: D2G4O<br>Cat #: 15372
Ki67 (Clone B56)	BD Biosciences	Clone ID: B56<br>Cat #: 556003
Biological samples		
FFPE human biopies of cHL	Brigham and Women's Hospital (Boston, MA, USA)	IRB# 2010P002736
FFPE human biopies of cHL	Dana-Farber Cancer Institute (Boston, MA, USA)	IRB# 2016P002769<br>IRB# 2014P001026
FFPE human biopsies of cHL	University of Rochester Medical Center (Rochester, NY, USA)	IRB# STUDY159
FFPE human biopsies of cHL	University Hospital and Comprehensive Cancer Center Tübingen (Tübingen, Germany)	N/A
Chemicals, peptides, and recombinant proteins		
Boca Scientific Inc Antibody Stabilizer PBS Base	Fisher Scientific	Cat #: NC0414486
TCEP	Thermo Fisher Scientific	Cat #: 77720

(*Continued on next page*)

Continued

REAGENT or RESOURCE	SOURCE	IDENTIFIER
VECTABOND® Reagent	Vector Labs	Cat #: SP-1800-7
Dako Target Retrieval Solution, pH 9	Agilent	Cat #: S236784-2
Recombinant Proteinase K Solution (20 mg/mL)	Thermo Fisher Scientific	Cat #: AM2546
Formalin, Neutral, Buffered 10% w/v in Phosphate Buffer	EMS Diasum	Cat #: 15740-04
SSC Buffer 20× Concentrate	Millipore Sigma	Cat #: S6639
Formamide, Molecular Biology Grade	Millipore Sigma	Cat #: 344206-1L
Buffer W	Nanostring	Cat #: 121300313
UltraPure™ DNase/RNase-Free Distilled Water	Invitrogen	Cat #: 10977023
BLOXALL Endogenous Peroxidase and Alkaline Phosphatase Blocking Solution	Vector Labs	Cat #: SP-6000-100
Hematoxylin QS Counterstain	Vector Labs	Cat #: H-3404-100
16% Paraformaldehyde	EMS Diasum	Cat #: 15740-04
Rat IgG	Sigma	Cat #: I4141-10mg
Mouse IgG	Sigma	Cat #: I5381-10mg
Sheared salmon sperm DNA	ThermoFisher	Cat #: AM9680
CODEX oligonucleotide block	Invitrogen	Cat #: AM9849
BS3 fixative	ThermoFisher	Cat #: 21580
Hoechst 33342	ThermoFisher	Cat #: H3570
Critical commercial assays		
Maxpar X8 Multimetal Labeling Kit	Fluidigm	Cat #: 201300
Ionpath Conjugation Kits	Ionpath	Cat #: 600XXX
Nanostring RNA Slide Prep kit	Nanostring	Cat #: 121300313
Nanostring Human Whole Transcriptome Atlas detection probe	Nanostring	Cat #: 121401102
ImmPRESS® HRP Universal Antibody (Horse Anti-Mouse/Rabbit IgG) Polymer Detection Kit, Peroxidase	Vector Labs	Cat #: MP-7500-50
DAB Substrate Kit, Peroxidase (HRP), with Nickel, (3,3′-diaminobenzidine)	Vector Labs	Cat #: SK-4100
Nanostring NGS library preparation kits	Nanostring	Cat #: 121400201<br>Cat #: 121400202<br>Cat #: 121400203<br>Cat #: 121400204
CODEX reporter plate	BRAND Tech	Cat #: 781607
Plate seal	ThermoFisher	Cat #: AB0626
CODEX barcode oligonucleotides	Biomers	N/A
CODEX reporter oligonucleotides	GenScript	N/A
CD8:MHC-I Atto647 proximity labeling kit	Navinci	Cat #: 60035
Deposited data		
All data in this sudy	This Study	Zenodo doi:10.5281/zenodo.18434741.
Data and code availability		
All code in this study	This study	Zenodo doi:10.5281/zenodo.18432666; https://github.com/SizunJiangLab/Hodgkin_EBV_MIBI
Software and algorithms		
Toffy package (v0.1.0)	Angelo lab	https://github.com/angelolab/toffy
Rosetta algorithm	Angelo lab	https://github.com/angelolab/toffy/blob/main/templates/4a_compensate_image_data.ipynb

(*Continued on next page*)

Continued

REAGENT or RESOURCE	SOURCE	IDENTIFIER
UNET (supervised deep learning-based segmentation)	Ronneberger et al. 2015	
Semi-supervised kNN-based clustering method	Baranski et al. 2021	
Deepcell-tf 0.6.0	Valen et al. 2016<br>Greenwald et al. 2021	https://github.com/vanvalenlab/deepcell-tf/releases/tag/0.6.0
Deepcell-tf 0.12.2	Valen et al. 2016<br>Greenwald et al. 2021	https://github.com/vanvalenlab/deepcell-tf/releases/tag/0.12.2
MIBI data processing pipeline	Shaban et al. 2023	https://github.com/mahmoodlab/MAPS
REinforcement Dynamic Spillover EliminAtion (REDSEA) method	Bai et al. 2021	https://github.com/nolanlab/REDSEA
FlowSOM	Gassen et al. 2015	https://bioconductor.org/packages/release/bioc/html/FlowSOM.html
Mantis Viewer	Schiemann et al. 2020	
Spatial-LDA	Chen et al. 2020	
PhenoGraph	Levine et al. 2015	
Nanostring GeoMx Data Analysis software pipeline	Merritt et al. 2020	
GeomxTools R package (v.3.6.2)		
standR (v.1.4.2)	Liu et al. 2024	
R package kBET (version 0.99.6).	Büttner et al. 2019	
GSVA package	Sun et al. 2021	
CellphoneDB	Efremova et al. 2020	
circlize R package (version 0.4.15)	Gu et al. 2014	
ggplot2R package		https://cran.r-project.org/web/packages/ggplot2/index.html
complexheatmap R package	Gu et al. 2016	
scipy 1.15.2		https://github.com/scipy/scipy/releases
networkx 3.4.2		https://github.com/networkx/networkx/releases
**Other**		
MIBIscope™ Xenon ion source (Hyperion, Oregon Physics)	Ionpath	N/A
GeoMx Digital Spatial Profiler	Nanostring	N/A
CosMx Spatial Molecular Imager	Nanostring	N/A
Phenocycler Fusion	Akoya Biosciences	N/A
ImmEdge® Hydrophobic Barrier PAP Pen	Vector Laboratories	H-4000
VWR® Premium Superfrost® Plus Microscope Slides	VWR	Cat #: 48311-703
Amicon® Ultra Centrifugal Filter, 50 kDa MWCO	Millipore Sigma	Cat #: UFC505096
NanoDrop	Thermo Fisher Scientific	Cat #: ND-2000
Grundium Ocus®40	Grundium	Cat #: MGU-00004
Fisherbrand™ Superfrost™ Plus Microscope Slides	Fisher Scientific	Cat #: 12-550-15
Microfiber cleaning cloths	Care Touch	Cat #: BD11945
VWR® Digital Mini Incubator	VWR	Cat #: 10055-006
Leica ST4020 Small Linear Stainer	Leica Biosystems	Cat #: ST4020
Epredia™ Lab Vision™ PT Module	Fisher Scientific	Cat #: A80400012
Hybridization coverslip	EMS Diasum	Cat #: 70329-40
DNA LoBind tubes	Eppendorf	Cat #: 022431021
AMPure XP beads	Beckman Coulter	Cat #: A63881
DynaMag™-2 Magnet	Thermo Fisher Scientific	Cat #: 12321D

(*Continued on next page*)

Continued

REAGENT or RESOURCE	SOURCE	IDENTIFIER
Agilent BioAnalyzer	Agilent	N/A
Collection plates	Nanostring	Cat #: 100473
Vectamount Mounting Medium	Vector Laboratories	H-5000-60

EXPERIMENTAL MODEL AND STUDY PARTICIPANT DETAILS

For MIBI analysis, formalin-fixed paraffin-embedded (FFPE) excisional biopsies from 20 patients with newly diagnosed cHL, and one reactive lymph node were retrieved from the archives of Brigham and Women's Hospital (Boston, MA) with institutional review board approval (IRB# 2010P002736). All tumor regions were annotated by K.W., V.S. and S.J.R. For GeoMx analysis, TMAs from two institutions were used. A Dana-Farber Cancer Institute TMA was constructed by S.S. and S.J.R. (IRB# 2016P002769 and 2014P001026), and includes 1 or 2 cores from each of 10 EBV-positive and 13 EBV-negative patients, as well as one tonsil control core, with each core measuring 1.5 mm in diameter. A University of Rochester Medical Center TMA was constructed by P.R. and W.R.B. (IRB# STUDY159), and includes 1 core from each of 12 EBV-positive and 11 EBV-negative patients, as well as one tonsil control core, with each core measuring 2.0 mm in diameter. For CODEX-PLA analysis, TMAs from two institutions were used: A TMA from University of Rochester (IRB# STUDY159) with 8 EBV-positive and 4 EBV-negative patients, and a TMA from University of Tübingen constructed by L.F., L.K., and C.M.S. with 7 EBV-positive and 8 EBV-negative patients. Tissues were sectioned onto gold slides (see below for more information) for the MIBI and SuperFrost glass slides (VWR, 48311–703) for the GeoMx and CODEX-PLA, with each section measuring 5 μm in thickness. As part of the routine clinical pathology process, all cHL biopsies were confirmed to have HRS cells by immunohistochemical staining for CD30, and EBV status verified using *in situ* hybridization for *EBER*. EBV status for each patient is reported in Table S2.

METHOD DETAILS

MIBI antibody conjugation

Antibody conjugation was performed according to a modified version of a previously published protocol.[77] Maxpar X8 Multimetal Labeling Kit (Fluidigm, 201300) and Ionpath Conjugation Kits (Ionpath, 600XXX) were utilized. Briefly, 100 μg of bovine serum albumin (BSA) free antibody was washed with the conjugation buffer, and then incubated with 4 μM TCEP (Thermo Fisher Scientific, 77720) for 30 min in a 37°C water bath to reduce the thiol groups for conjugation. The reduced antibody was subsequently incubated with Lanthanide-loaded polymers for 90 min in a 37°C water bath. The resulting conjugated antibody was purified by washing five times with an Amicon Ultra filter with 50 kDa NMWL (Millipore Sigma, UFC505096). The conjugated antibody was quantified in IgG mode at A280 using a NanoDrop (Thermo Scientific, ND-2000). The final concentration was adjusted by adding at least 30% v/v Candor Antibody Stabilizer (Thermo Fisher Scientific, NC0414486) supplemented with 0.2% sodium azide, and the antibody was stored at 4°C.

Antibody panel titration and validation

The antibody candidates used for sample staining throughout the study contain previously validated antibody clones.[24,60,78] In brief, antibody candidates were first validated for specificity via traditional immunohistochemistry (IHC) to ensure compatibility and robustness of staining. Working clones were then conjugated as described above, and subject to validation and titration on the MIBI platform as previously described.[24] All final images were visually inspected by S.J.R., a board-certified hematopathologist. Fluorophore-conjugated and oligonucleotide-conjugated antibodies respectively used for the GeoMx and CODEX-PLA were also validated via immunofluorescence. Details regarding the antibody clones, vendors, conjugated channels, and titers can be found in Table S1. Readers of interest are referred to the following publications for a more detailed guide on antibody target selection and optimization.[79–81]

MIBI gold slide preparation

The procedure for preparing gold slides was previously described in several studies.[78,82,83] Briefly, superfrost plus glass slides (Thermo Fisher Scientific, 12-550-15) were first soaked in ddH $_2$ O and then cleaned by gently rubbing with microfiber cleaning cloths (Care Touch, BD11945) and diluted dish detergent. Subsequently, the slides were rinsed with flowing ddH $_2$ O to eliminate any residual detergent, then air-dried using a continuous stream of airflow to remove water droplets. The process of coating the slides with 30 nm of Tantalum followed by 100 nm of Gold was performed by the Microfab Shop at Stanford Nano Shared Facility (SNSF) or by New Wave Thin Films (Newark, CA).

MIBI slides vectabonding

Coated gold slides were silanized by VECTABOND Reagent (Vector Laboratories, SP-1800-7) per the protocol provided by the manufacturer. The slides were first quickly rinsed with ddH $_2$ O to remove dust, then soaked in neat acetone for 5 min, and transferred

into 1:50 diluted VECTABOND reagent in acetone and incubated for 10 min. Slides were then quickly dipped in ddH_2O multiple times to quench and remove remaining reagents. Remaining water was removed by tapping over Kimwipe without rubbing, air-dried at room temperature (RT) or 37°C overnight, and stored subsequently at RT.

MIBI staining

The procedure of the MIBI staining follows previously described methods.[24,81,82,84] Briefly, slides with FFPE sections were baked in an oven (VWR, 10055–006) at 70°C for 1 h, then immersed in xylene and incubated for 2 × 10 min to thoroughly remove the paraffin. The slides were then subject to a series of solutions for deparaffinization and rehydration using a linear stainer (Leica Biosystems, ST4020): 3× xylene, 3 × 100% EtOH, 2 × 95% EtOH, 1 × 80% EtOH, 1 × 70% EtOH, 3× ddH_2O, 180 s each step with constant dipping, and left in ddH_2O. Antigen retrieval was then performed at 97°C for 10 min with Target Retrieval Solution (Agilent, S236784-2) on a PT Module (Thermo Fisher Scientific, A80400012). After PT Module processing, the cassette containing the slides and solution was allowed to cool to RT on the benchtop. Following a brief minutes rinse with 1× PBS, tissue regions were circled with a PAP pen (Vector Laboratories, H-4000) and then blocked using BBDG (5% normal donkey serum (NDS), 0.05% sodium azide in 1× TBS IHC wash buffer with Tween 20), before an overnight incubation at 4°C with the antibody cocktail (Table S1). The next day, slides were subject to 3 × 5 min washes with the washing buffer (1× TBS IHC wash buffer with Tween 20 and 0.1% BSA) to remove unbound and non-specific antibodies. The samples were briefly rinsed with 1× PBS, fixed with a post-fixation buffer (4% PFA +2% glutaraldehyde in 1× PBS buffer) for 10 min, and quenched with 100 mM Tris HCl (pH 7.5). The slides then underwent a series of dehydration steps on the linear stainer (3 × 100 mM Tris pH 7.5, 3× ddH_2O, 1 × 70% EtOH, 1 × 80% EtOH, 2 × 95% EtOH, 3 × 100% EtOH) with 60 s for each step. Dried slides were stored in a vacuum desiccator until acquisition.

MIBI-TOF imaging and image extraction

Datasets were acquired using a commercially available MIBIscope system from Ionpath, which is equipped with a Xenon ion source (Hyperion, Oregon Physics). The typical operation parameters for the instrument are listed below:

Production MIBI.

- Pixel dwell time: 2 ms
- Image area: 400 × 400 μm
- Image size: 512 × 512 pixels
- Probe size: ~400 nm
- Primary ion current: Fine mode (5–5.5 nA on a built-in Faraday cup)
- Number of planes: 1 depth

The single-channel MIBI images were extracted from raw files generated by the MIBIscope machine using the toffy package (v0.1.0) developed by the Angelo lab https://github.com/angelolab/toffy. For the extraction of most mass channels, a mass range of [-0.25, 0] was employed, while for the 113-Histone H3 channel, a mass range of [-0.25, 0.25] was used.

MIBI channel crosstalk removal

Mass spectrometry based analysis and imaging methodologies such as MIBI can suffer from channel crosstalk caused by adduct formation[83] or isotopic impurities in the elemental labels used. Thus, the Rosetta algorithm was applied to the extracted raw images to remove noise resulting from channel crosstalk with a similar manner as compensating flow cytometry data https://github.com/angelolab/toffy/blob/main/templates/4a_compensate_image_data.ipynb. Similarly, background signals arising from bare slides or organic fragments can be partially reflected by the gold and "Noodle" background channels. Together, a fine-tuned coefficient matrix was used to remove those channel crosstalk using a local implementation of toffy package (v0.1.0) with minimal modification.

MIBI image denoising

The additional noise apart from the channel crosstalks were further removed with a deep learning-based method developed by M.S. and F.M., which poses image denoising as a background-foreground segmentation problem. The underlying concept involves considering the genuine signal as the foreground and the noise as the background. The proposed method employs a supervised deep learning-based segmentation model called UNET,[85] to accurately segment the foreground from the given image. To train the model, ground truth data was first generated using a semi-supervised kNN-based clustering method.[86] Once the model is trained, it is applied to all markers in all images, producing predicted foreground segmentation maps. These segmentation maps are then multiplied with the original images, eliminating noise and yielding clean images. We observed subpar signal to noise for the GATA3 signals, as noted by a lack of robust GATA3 staining in T cells in reactive tonsil. We note that the antibody exhibited signals on HRS cells and was thus used to guide HRS cell phenotyping, but was excluded from downstream analyses.

MIBI cell segmentation

Cell segmentation of the cHL MIBI datasets was performed using a local implementation of deepcell-tf 0.6.0 as described.[87,88] Histone H3 channel was used for the nucleus, while the summation of HLA-DR, HLA1, Na-K-ATPase, CD45RA, CD11c, CD3, CD20, and

CD68 was used as the membrane feature. Prior to input into the model, the signals from these channels were capped at the 99.7th percentile. The deepcell-tf version used to generate the final segmentation mask, along with the detailed parameters, are summarized in Table S3.

MIBI image intensity normalization

Due to the inherent limitations of the MIBI instrument, an FOV routinely acquired is restricted to a size of 400 × 400 μm. Thus, most tiles of the cHL MIBI dataset are composed of multiple stitched adjacent FOVs. Within each tile, the inter-FOV signal level difference and boundary effects were corrected with a series of publicly available scripts as previously described.[24,89]

MIBI image to cell expression matrix, REDSEA signal compensation, and across-runs normalization

To generate the cell expression matrix, the counts of each marker within each segmented cell were summed and then divided by the corresponding cell size. This process utilized the normalized stitched TIFs and their respective segmentation masks. To address signal spillover between adjacent cells, REDSEA was applied to the extracted cell expression matrix along with the segmentation mask, as previously described.[26] It was observed that Histone H3 signal is positively correlated with most markers, which means that if an FOV has higher Histone H3 signal, the signal of other markers of that FOV would also tend to be higher. Thus, to minimize the unwanted intensity variation between tiles and to make the same channel more comparable across different tiles, the median of Histone H3 counts was found for each tile. Then, the intensity of each marker within each tile was divided by its corresponding median Histone H3.

MIBI cell phenotyping

Cell phenotyping of the MIBI dataset was performed through an iterative clustering and annotating process with FlowSOM.[90] The scaled cell expression matrix was initially clustered with CD11c, CD14, CD15, CD153, CD16, CD163, CD20, CD3, CD30, CD4, CD56, CD57, CD68, CD8, FoxP3, GATA3, Granzyme B, and Pax-5 to capture most of the cell phenotypes present in the dataset. The resulting clusters were then manually annotated by examining the predominantly enriched markers of each cluster, which was done by plotting *Z* score and mean expression heatmaps across all clusters and the phenotypic markers used. Clusters with a clear enrichment pattern were annotated, while clusters with mixed pattern underwent additional rounds of FlowSOM clustering and annotation. To confirm the assigned annotations, Mantis Viewer[91] was utilized. The annotation for each cell cluster was mapped and the raw images of the enriched markers were overlaid for visual inspection. This interactive process was repeated until no additional useful information could be extracted. Note that HRS cells were annotated primarily through identifying multinucleated cells with dim Pax5 staining with CD30 serving as a secondary marker, but CD30 was used for visualization in the figures for the general readership. Cells within clusters that lacked clear enrichment patterns were assigned as "Others". For the MIBI dataset, a total of 1,538,433 out of 1,669,853 cells (92.2%) were successfully assigned a final annotation. To ensure the accuracy of the annotations, all final annotations were assessed by S.J. and S.J.R.

GeoMx staining

Tissue slides were prepared with modifications from the official Nanostring GeoMx-NGS RNA Manual Slide Preparation protocol. Deparaffinization, rehydration, and antigen retrieval on the two TMA tissue slides were performed using the same procedures as described under the MIBI Staining protocol. Tissue slides were then allowed to cool to RT on the benchtop, and washed in 1× PBS for 5 min at RT. Next, they were digested by Proteinase K (0.5 μg/mL) (Thermo Fisher Scientific, AM2546) for 5 min at 37°C, and then washed in 1× PBS for 5 min at RT. Subsequently, tissue slides were fixed in 10% neutral buffered formalin (NBF) (EMS Diasum, 15740–04) for 5 min at RT. The fixation process was stopped by incubating twice in 1× NBF stop buffer (0.1 M Tris and 0.1 M Glycine) for 5 min each at RT, followed by a 1× PBS wash for 5 min at RT. Tissue slides were then transferred to fresh 1× PBS while the RNA probe staining cocktail was prepared using the Nanostring RNA Slide Prep kit (Nanostring, 121300313), by combining the Nanostring Human Whole Transcriptome Atlas detection probe (Nanostring, 121401102) set with a custom spike-in panel of probes against 14 targeted EBV genes (*EBER1*, *EBER2*, *EBNA1*, *EBNA2*, *EBNALP*, *LMP1*, *RPMS1*, *BALF1*, *BCRF1*, *BHRF1*, *BNLF2A*, *BNLF2B*, *BNRF1*, *BZLF1*). The RNA probe staining cocktail was then applied to the tissue slides, sealed with a hybridization coverslip (EMS Diasum, 70329–40), and incubated overnight (~16 h) at 37°C. After RNA probe hybridization, tissue slides were washed twice in Stringent Wash Buffer (2× saline sodium citrate (SSC) (Millipore Sigma, S6639) in 50% formamide (Millipore Sigma, 344206-1L-M)) for 5 min each at 37°C. Tissues were blocked with Buffer W (Nanostring, 121300313) for 30 min, followed by antibody staining for 1 h with antibodies against CD3, CD4, and CD45RO, as well as SYTO13 (100 nM) to indicate nucleus and nuclear morphologies. Tissue slides were washed twice in 2× SSC for 2 min each at RT, then stained with anti-mouse antibody for 20 min at RT. Slides were washed twice again in 2× SSC for 2 min each at RT, and then loaded onto the GeoMx to be scanned and selection of region of interest (ROI).

IHC staining

To guide ROI selection and HRS cell annotation, we performed IHC on an adjacent TMA section for both TMAs with CD30 to identify HRS cells. Deparaffinization, rehydration, antigen retrieval, and cooling to RT for these TMA tissue slides were performed in parallel with those used for GeoMx (see above section). Tissue slides were then washed in UltraPure water (Invitrogen 10977–023) for 5 min at RT, blocked with peroxidase and alkaline phosphatase blocking solution (Vector Laboratories SP-6000-100) for 10 min at RT, and

washed with washing buffer (1× TBS-T, 0.1% BSA). Tissue sections were then blocked with 2.5% Normal Horse Serum (Vector Laboratories MP-7500-50) for 1 h at RT, and stained overnight at 4°C with anti-CD30 antibody diluted in antibody diluent buffer (1× TBS-T, 5% donkey serum, 0.05% sodium azide). Tissue slides were then washed twice in washing buffer, and then stained with secondary anti-mouse IgG and anti-rabbit IgG HRP-conjugated antibodies for 30 min at RT (Vector Laboratories, MP-7500-50). After washing twice in washing buffer for 5 min each, the DAB peroxidase substrate (Vector Laboratories SK-4100) was introduced, and brown coloration was allowed to develop over 2.5 min. Tissue slides were briefly rinsed in tap water and counterstained with hematoxylin (Vector Laboratories H3404-100) for 2 min, and blue coloration was allowed to develop by rinsing in tap water for five times at 3 min each. Finally, tissues were dried in an oven (VWR 10055–006) at 70°C for 20 min, mounted on a glass coverslip using Vectamount Permanent Mounting Medium (Vector Laboratories H-5000-60), and digitized using a Grundium Ocus40 microscope (Grundium MGU-00004) to identify CD30-positive cells.

GeoMx ROI selection

Individual ROIs were selected at regions that contain abundant T cells and HRS cells, by referring to CD3 and CD30 staining patterns respectively, the latter determined through IHC staining on adjacent TMA tissue sections. The ROIs drawn were focused on regions densely populated in tumor cells. ROI selections were also optimized to ensure that at least 2 ROIs were obtained from each patient. A total of 126 ROIs (62 EBV-positive, 62 EBV-negative, 2 tonsil) were drawn across both TMAs, and each ROI was drawn as 760 × 660 μm rectangles (501,600 μm^2 area each). ROIs were then exported from the GeoMx for cell segmentation and annotation, in order to obtain cell masks that guide transcript extraction from cell populations of interest.

GeoMx cell segmentation

Cell segmentation of the GeoMx datasets was performed similarly, but instead using a local implementation of deepcell-tf 0.12.2.[87,88] SYTO13 channel was used for the nucleus, while the summation of CD3, CD4, and CD45RO was used as the membrane feature. Prior to input into the model, the signals from these channels were capped at the 99.7th percentile. The deepcell-tf version used to generate the final segmentation mask, and the detailed parameters are also summarized in Table S3.

GeoMx cell phenotyping

Regions corresponding to HRS cells were identified based on IHC staining, and HRS cells were manually identified based on nuclear (SYTO13) morphology and adjacent CD30 staining patterns. The visualization of cell nucleus and cells around tumor regions was performed through Mantis Viewer.[91] Background signals from CD3, CD4, and CD45RO channels in the GeoMx dataset were first removed by applying percentile cutoffs and visually validated. Cell phenotyping of the GeoMx dataset was performed through PhenoGraph,[92] with k = 100 and using CD3, CD4, and CD45RO to identify memory T cells, naive T cells within the TME. The remaining cells which were unidentifiable based on CD3, CD4, and CD45RO were categorized as "other cells". Finally, Mantis Viewer[91] was utilized to confirm all assigned annotations, where the annotation for each cell phenotype was overlaid over the enriched markers for visual inspection. Binary cell masks corresponding to these 6 cell phenotypes of interest for each ROI were then generated to facilitate downstream transcript extraction through the GeoMx. The marker combinations corresponding to each cell type can be found in Table S5.

GeoMx transcript extraction and sequencing library preparation

Cell masks were imported into the GeoMx, and transcripts for each cell phenotype across every ROI were aspirated based on the order described in the prior section. In total, 8 collection plates (Nanostring, 100473) were used to collect all aspirates, and aspirates were dried at RT overnight and resuspended in 10 μL of UltraPure water (Invitrogen 10977–023). Each aspirate was then uniquely indexed using the Illumina i5 × i7 dual indexing system through Nanostring NGS library preparation kits (Nanostring, 121400201, 121400202, 121400203, 121400204). The PCR reaction was prepared in 96-well plates, where each well contained 4 μL of aspirate, 1 μM of i5 primer, 1 μM of i7 primer, and 1× library preparation PCR Master Mix, adding up to 10 μL in total volume. The PCR reaction conditions were 37°C for 30 min, 50°C for 10 min, 95°C for 3 min, followed by 18 cycles of 95°C for 15 s, 65°C for 60 s, 68°C for 30 s, followed by a final extension of 68°C for 5 min before holding at 4°C. Next, 4 μL of PCR products from each plate were then pooled into DNA LoBind tubes (Eppendorf 022431021) for purification, where 1.2× volume of AMPure XP beads (Beckman Coulter A63881) were first added to the pooled PCR products and at RT for 5 min. Beads were then pelleted on a magnetic stand (Thermo Fisher Scientific 12321D), washed twice with 1 mL of 80% ethanol, and eluted with 54 μL of elution buffer (10 mM pH 8.0 Tris-HCl, 0.05% Tween 20). Another round of purification was performed using 50 μL of eluted DNA in the same approach, using 1.2× volume of AMPure XP beads and washing twice in 1 mL of 80% ethanol. A final elution was done at 2:1 ratio of aspirate (number of wells) to elution buffer (volume in μL), and 0.2 μL of the final eluate was diluted in 9.8 μL of UltraPure water (Invitrogen 10977–023) (1:10 dilution) to confirm library purity through Agilent BioAnalyzer. Finally, libraries were paired-end sequenced on NovaSeq6000 with a sequencing depth of ~7.5 billion reads, which is at least 1.2× greater than recommended by the official protocol.

GeoMx transcript mapping and counting

The NGS barcodes from the Nanostring human WTA panel and custom EBV-specific probes were mapped and counted using the commercial GeoMx Data Analysis software pipeline,[21] using FASTQ files generated from NGS sequencing. Subsequently, the '.dcc' files produced by the GeoMx Data Analysis software pipeline were used as input to generate the gene-level counts table. R package

GeomxTools (v.3.6.2) with default setting was implemented, and raw gene counts table was produced and used for normalization and batch effect correction.

GeoMx data normalization and batch effect removal

The standR (v.1.4.2) workflow included normalization, negative control gene (NCG) searching, and batch effect removal using the RUV4 method,[93] and thus was used to normalize raw gene counts and reduce patient-level batch effects induced by individual variability.[93] To fit the best practice of batch effect removal, a grid search was implemented to find the optimal hyperparameter combinations that minimized individual variations while keeping EBV condition and cell type variations. By following the standR workflow, four normalization methods were adopted, including the trimmed mean of M-values (TMM), log counts-per-million reads (CPM), quantile normalization, and size factor normalization. Nine grids of the number of NCG genes were selected (100, 300, 500, 700, 900, 1100, 1300, and 1500). The five grids of the number of k-covariance matrices for the RUV4 method were set to 3, 5, 7, 9, and 11. In total, 180 parameter sets were evaluated.

GeoMx data assessment after batch effect removal

The effect of batch correction was assessed using the silhouette and the kBET scores,[94] as implemented in the R package kBET (version 0.99.6). The silhouette score quantifies cluster separation, with a higher score signifying more distinct clustering. The kBET assessment involved quantifying the rejection rate through Pearson's chi-square test and comparing the distribution of local and global batch labels among the k-nearest neighbors. Specifically, a high kBET score of a factor indicated that less bias was introduced by this factor. Both silhouette and kBET scores were used to evaluate the consistency across all samples in terms of patient factor for individual variations, cell type, and EBV condition factor for biological variations. Therefore, the objective of the standR workflow's grid search was to identify a parameter set that minimized the batch effect related to the patient factor while maximizing the differentiation by cell type and EBV condition in the following steps. First, post-corrected expression matrices were derived based on the standR pipeline under a specific parameter set. Second, silhouette and kBET scores for each parameter set were computed according to three factors. Third, adopting the concept from the previous study,[95] silhouette and kBET scores were ranked in following rules. Specifically, scores determined by the patient factor were ranked in ascending order, where the lowest score received ranked one, and the highest score was assigned ranked 180. Scores determined by cell type and EBV condition were ranked in decreasing order, where the highest score received ranked one, and the lowest score was assigned ranked 180. Lastly, an overall rank was calculated by averaging the rank of three factors and two scores, and the optimized parameters were selected based on the overall rank. The batch effect corrected data was then used for all subsequent analysis.

The detailed evaluation scores during the batch effect correction process can be found in Table S7, which provides an assessment of different parameters set against various factors (e.g., patient, cell type (CT), and EBV status), while incorporating both kBET and silhouette scores as well as their corresponding ranks to evaluate batch effect correction.

CosMx data processing

The CosMx data was generated through Technology Access Program (TAP) with nanostring using a 1k-plex RNA probe panel added with custom designed probes against 8 EBV transcripts (*BCRF1*, *BLLF1*, *BZLF1*, *EBNA2*, *EBNA3A*, *EBNA3BC*, *LMP1*, *RPMS1*). The TMA cores to prioritize here were selected after pathology review with S.J.R. for the present of tumor cells, and subsequent capture regions within the cores here were randomly selected within the TMA core. The raw data consisting of table of transcript positions was obtained from the CosMx platform and subjected to cell segmentation using Proseg, which is based on an unsupervised probabilistic model of the spatial distribution of transcripts.[96] Afterward, data from these two TMAs were pooled and subjected to multi-dataset integration using Harmony. Data from two TMAs containing a total of 43 cores (1 FOV each, 21 EBV negative and 22 EBV positive) were then merged and subjected to multi-dataset integration using Harmony.[97]

CODEX-PLA antibody conjugation

Antibody conjugation was performed according to a modified version of a previously published protocol.[98] Briefly, 100 μg of carrier-free antibody was concentrated using a 50kDa filter (Sigma Millipore, UFC5050BK) pre-wetted with 1× PBS-T, and then incubated with 0.9 μM TCEP (Sigma, C4706-10G) for 15–30 min in a 37°C water bath to reduce the thiol groups for conjugation. Reduction was quenched by two washes with Buffer C (1 mM Tris pH 7.5, 1 mM Tris pH 7.0, 150 mM NaCl, 1 mM EDTA) supplemented with 0.02% sodium azide. Maleimide oligos were resuspended in Buffer C supplemented with 250 mM NaCl, and the reduced antibody was subsequently incubated with 200 μg of maleimide oligos (Biomers, 5′-Maleimide) in a 37°C water bath for 2 h. The resulting conjugated antibody was purified by washing thrice with high-salt PBS (1× DPBS, 0.9M NaCl, 0.02% sodium azide) using a 50kDa filter. The conjugated antibody was quantified under IgG mode at A280 using a NanoDrop (Thermo Scientific, ND-2000). The final concentration was adjusted by adding at least 30% v/v Candor Antibody Stabilizer (ThermoFisher, NC0414486) supplemented with 0.2% sodium azide, and the antibody was stored at 4°C.

CODEX-PLA antibody staining

The antigen retrieval step and pap pen drawing is identical as the MIBI Staining protocol described above, and the procedure of the CODEX-PLA staining follows previously described methods.[98,99] Tissues after PLA were blocked using BBDG supplemented with

50 μg/mL mouse IgG (Sigma, I5381-10mg), 50 μg/mL rat IgG (Sigma, I4141-10mg), 500 μg/mL sheared salmon sperm DNA (ThermoFisher, AM9680), and 50 nM oligonucleotide block (contains each CODEX oligonucleotide (Invitrogen, AM9849)) for 1 h before an overnight incubation with the antibody cocktail (Table S1). The next day, tissues were washed in S2 Buffer (2.5 mM EDTA, 0.5× DPBS, 0.25% BSA, 0.02% sodium azide, 250 mM NaCl, 61 mM Na_2HPO_4, 39 mM NaH_2PO_4) 2 × 2 min each at RT. The tissues were first fixed in 1.6% PFA (diluted from 16% stock (EMS Diasum, 15740–04) in S4 Buffer (4.5 mM EDTA, 0.9× DPBS, 0.45% BSA, 0.02% sodium azide, 500 mM NaCl)) 2 × 5 min each at RT, after which the tissues were rinsed twice in 1× PBS followed by a 2 min wash in 1× PBS. The tissues were next fixed with ice-cold methanol for 5 min, after which the tissues were immediately rinsed 2× in 1× PBS followed by another 2 min wash in 1× PBS. The tissues were finally fixed in 4 μg/μL of BS3 Final Fixative (diluted from 200 μg/μL stock (ThermoFisher, 21580) in 1× PBS) 2 × 10 min each in the dark at RT, after which the tissues were rinsed twice in 1× PBS followed by a final 2 min wash in 1× PBS.

CODEX-PLA antibody panel imaging

A reporter plate was first prepared for each tissue such that each well corresponds to each imaging cycle. Briefly, a 96-well black reporter plate (BRAND Tech, 781607) was prepared by filling each well with plate buffer (500 μg/mL sheared salmon sperm DNA in 1× CODEX buffer (10 mM Tris pH 7.5, 0.02% sodium azide, 0.1% Triton X-100, 10 mM $MgCl_2$-6H_2O, 150 mM NaCl) supplemented with 1:300 (54.11 mM) of Hoechst 33342 (ThermoFisher, H3570), adding 100 nM of each complementary reporter oligonucleotides conjugated with either an ATTO550 or Alexa Fluor 647 (GenScript, HPLC purified) fluorophore, then sealed using aluminum plate seal (ThermoFisher, AB0626). The first and last wells did not contain any reporter oligonucleotides for the blank channels.

The CODEX microfluidics was set up by loading the reporter plate and buffer bottles (ddH_2O, DMSO, and 1× CODEX buffer), and emptying the waste bottle. The tissue was stained with 300 μL of nuclear stain solution (1:1000 Hoechst 33342 (ThermoFisher, H3570) in 1× CODEX buffer) for 3 min and then washed 3× with 1× CODEX buffer, after which the nuclear stain was used to visualize tissue morphology to select imaging areas. Each imaging cycle contained three channels: nuclear stain imaged at 33 ms, and two markers on the Cy3 and Cy5 filter channels imaged at 250 ms. Images taken on the Cy3 and Cy5 filter channels during the first and last blank cycles were used as blanks for background subtraction. CODEX imaging was operated using the Phenocycler Fusion (Akoya Biosciences).

CODEX-PLA interaction detection and imaging

After the CODEX run was performed, slides were subjected to another antigen retrieval as described above, but for 10 min to remove bound antibodies. PLA was then performed according to manufacturer's protocol using the CD8:MHC-I Atto647 kit (Navinci, 60035).

After PLA staining, tissues were then washed twice in 1× TBS with gentle agitation. Tissues were then stained with nuclear stain solution (1:1000 Hoechst 33342 (ThermoFisher, H3570) in 1× CODEX buffer) for 3 min and then washed 3× with 1× CODEX buffer, after which the nuclear stain was used to visualize tissue morphology to select imaging areas. The slide was then scanned on the Phenocycler Fusion (Akoya Biosciences) and aligned back to the original CODEX-PLA image stack.

CODEX-PLA image processing

In order to maximize the signal to noise ratio (SNR) and thus ensure the robustness of the downstream analyses, a biology-informed background subtraction was performed on the CODEX images. Ideally, nuclear markers should only stain the nucleus of a cell and membrane markers should only stain the membrane of a cell. Based on the assumption, a nuclear mask was generated by binarizing the DAPI channel of a tissue, and then the non-nuclear mask can be obtained by finding the complement of the nuclear mask. With the two masks, the background of a marker can be inferred from the pixel values within them. If a marker is a nuclear marker, the background was inferred by taking the median of non-zero pixel values sampled between the 90th and the 100th quantile of the non-nuclear part of the image with a step of 0.0001. On the other hand, if a marker is a membrane marker, the background was inferred by taking the median of non-zero pixel values sampled between 50th and the 100th quantile of the nuclear part of the image with a step of 0.0001. The inferred background level was then subtracted from all pixels within the image.

The raw PLA images also included background signal and auto-fluorescence emitted by erythrocytes. A two-step thresholding was performed to ensure that only accurate PLA punctate signals were retained, and to enable optimal data input for the spot-counting algorithm. The first threshold was determined using moment-preserving thresholding.[100] Pixels less than the threshold were from background signal and were filtered out. Then, Yen's thresholding[101] was applied to the images to extract the auto-fluorescence emitted by erythrocytes.

CODEX-PLA cell segmentation

Cell segmentation of the CODEX-PLA datasets was performed using a local implementation of deepcell-tf 0.12.2[87,88] as described above. DAPI channel was used for the nucleus, while the summation of CD11c, CD3, CD4, CD68, CD163, HLA1, HLA-DR was used as the membrane feature. The deepcell-tf version used to generate the final segmentation mask, and the detailed parameters were summarized in Table S3.

CODEX-PLA cell phenotyping

Similar to MIBI cell phenotyping, the scaled single-cell features of the CODEX-PLA dataset were iteratively clustered using PhenoGraph[92] and annotated while using Mantis Viewer[91] to visually confirm each assigned annotation by overlaying clusters

CellPress
OPEN ACCESS

with the respective enriched markers. A similar strategy was used for HRS cell annotation, where cell morphology and dim Pax5 were the primary features followed by CD30 as a secondary marker. Remaining cells which were unidentifiable based on sparse expression of the phenotyping markers listed above were assigned as "Others". A total of 934478 out of 1059112 cells (88.2%) were successfully assigned a final annotation. All annotations were visually confirmed by S.J.R.

QUANTIFICATION AND STATISTICAL ANALYSIS

MIBI cell level analysis

Expression heatmap and cell type enrichment heatmap

The expression heatmap shows the enrichment of specific markers within a certain cell type when compared with the average expression levels across all cell types. Let $\mathbf{I} = \{1, \ldots, n\}$ denotes the indices of the lineage and functional markers of interest; $\mathbf{J} = \{1, \ldots, m\}$ denotes the indices of the cell types identified; $\mathbf{m}_i$ denotes the vector of marker expression for all cells; $\mathbf{m}_{i,j}$ denotes the vector of marker expression for all the j cell type. The sample mean marker expression for marker i, μ_i, the within j cell type mean marker expression for marker i, $\mu_{i,j}$, and the sample standard deviation for marker i, s_i, were calculated. Then, for each pair of i and j,

$$z_{i,j} = \frac{\mu_{i,j} - \mu_i}{s_i}$$

was calculated and presented in the expression heatmap. For the cell type enrichment heatmap shown in Figures 3A and 7B, the same procedure was followed except that marker expression was substituted by cell count.

Cell neighborhood calling by spatial LDA

Cell neighborhoods were identified using spatial LDA,[42] with parameters admm_rho = 0.1, primal_dual_mu = 10^5, and *max*_dirichlet_ls_iter = 100. In order to find the optimal number of neighborhoods which strikes a balance between resolution and legibility, we fitted the model with $n \in \mathbf{N} = \{3,4,5,6,7,8,9,10,11\}$ neighborhoods and plotted a heatmap for cell preference for each model. Starting from $n = 8$, the cell preference of each neighborhood started to stabilize. Meanwhile, a neighborhood with strong preference of "Others" cell type emerged, starting from $n = 9$. Therefore, $n = 8$ was picked as the optimal number of neighborhoods.

Cell phenotype and cell neighborhood enrichment

The cell phenotype enrichment of each cell type j was calculated as

$$\log_2\left(\frac{n_{j,EBV+} / n_{EBV+}}{n_{j,EBV-} / n_{EBV-}}\right).$$

Here, $n_{j,EBV+}$ is the number of cell for cell type j among all the EBV+ patients; $n_{j,EBV-}$ is the number of cell for cell type j among all the EBV- patients; n_{EBV+} and n_{EBV-} are the total number of cells among EBV+ and EBV- patients respectively. If a cell type is more prevalent in EBV+ patients than EBV- patients, the log_2 fold enrichment would be positive. Otherwise, the log_2 fold enrichment would be negative. If a cell type is equally enriched in both EBV status, the log_2 fold enrichment would be 0. The same approach was adopted in Figure 5C.

Simpson's diversity index calculation

The Simpson's Diversity Index was calculated based as

$$D = 1 - \sum p_i^2.$$

on a cell neighborhood level, where p_i is the proportion of cell type i in each tissue. Therefore, a larger D represents greater cellular diversity. The SDI was implemented using the R package 'vegan' (v2.6.10).

Tumor score and tumor density classification

For each non-tumor cell, a tumor score was calculated based on its distance to tumors within a closed neighborhood of radius r. Let $\mathbf{J} = \{1, \ldots, m\}$ denote the indices of all the tumors in the dataset and $d_{i,j}$ denote the distance from the cell i to tumor j. Then, the tumor score is calculated as

$$S_i = \sum_{j \in \{k | d_{i,j} \le r\}} \frac{1}{d_{i,j}}$$

Then, non-tumor cells were classified into tumor dense or tumor sparse based on their tumor score. The cut-off that separated tumor dense and tumor sparse classes was found by identifying the tangent point with the steepest slope to the right of the peak of the distribution of the tumor score.

Immune dysfunction level stratified by tumor density and EBV status

Non-tumor cells' dysfunction level was defined based on combinations of functional markers. For CD4 T cell, CD8 T cell, CD4 CTL, CD8 CTL and Treg, their dysfunction score was defined as

$$\gamma_{CD45RO} - \gamma_{CD45RA} + \gamma_{Tox} + \gamma_{PD-1} - \gamma_{Ki-67} + \gamma_{Lag3},$$

where γ_{marker} stands for the intensity of the specified marker. For DC, M1-like, and M2-like cells, their dysfunction score was defined as

$$\gamma_{PD-L1} - \gamma_{Ki-67}.$$

Then, cells were stratified into (tumor dense,*EBV*+), (tumor sparse,*EBV*+), (tumor dense,*EBV*-), and (tumor sparse,*EBV*-). A one-sided paired *t* test was conducted to compare the dysfunction score between tumor dense and tumor sparse regions within a given EBV status for each of the above mentioned cell types, with the alternative hypothesis that cells in tumor dense regions are more exhausted than those in tumor sparse regions. The test results were corrected for multiple comparisons via Benjamini-Hochberg procedure with a targeted false discovery rate (FDR) of 0.05. The unadjusted *p*-value and Benjamini-Hochberg procedure statistics and corrected test results can be found in Table S4. The dysfunction score approach was similarly adopted for downstream CODEX-PLA analyses in Figure 7F.

Bulk RNAseq analysis

The RNAseq paired-end reads were obtained from[102] that consisted of EBV-positive GM12878 cell lines untreated ($n = 3$) or treated ($n = 3$) with LMP1-targeted knockdown. The reference genome was generated by concatenating the hg38 human and EBV Akata strain (GenBank: GCA_900004315.1) genomes, which was used for aligning fastq reads using STAR (v2.7.11a). Transcript abundances were then quantified using salmon (v1.10.2) and scaled by transcript length and library size using the R package 'tximport' (v1.26.1). The expression of *CD274* was then quantified and compared.

GeoMx data analysis

General analysis related to gene expression

To identify significantly upregulated genes in each of the 6 region types (CD4 memory, CD8 memory, CD4 naive, CD8 naive, Tumor, and Other), the function 'findallmarkers' from the R package 'seurat' were implemented. For visualization, only the top 30 significant genes ($p.adj < 0.05$) with the highest log-fold change were plotted in the heatmap. Differentially expressed genes were identified by R package 'limma' with default parameters. G2M scores were calculated in the Tumor regions, with function 'CellCycleScoring' from R package 'seurat'. GSVA scores (gene pathway scores) were calculated using the function 'gsva' from R package 'GSVA' (v.1.42.0) with default parameters.

Receptor-ligand interaction analysis

Statistical inference of chemokine receptor-ligand interactions between tumor and T cell regions were performed via CellphoneDB v5[49,50] using the repository of chemokines curated within. Briefly, after quantifying the co-expression of receptor-ligand pairs between T cell and tumor regions within each ROI, an expression heatmap was generated based on the mean co-expression score across all ROIs. Statistical significance was assessed by comparing receptor-ligand interactions against a null distribution that was generated by simulating random receptor-ligand interactions across 1000 iterations.

Regression analysis for clinical metadata

A linear regression model, with the function 'lm' in R, was implemented to evaluate the contribution of each patient clinical feature (see Table S2 for the clinical features) to progenitor and terminal T cell exhaustion signature[56] GSVA scores.

Analysis related to EBV genes and T cell functional state

GSVA scores for CD8 and CD4 T cells were first treated as T cells. Next, within the same tissue core, pearson correlation was applied between T cell terminal GSVA exhaustion scores and individual EBV genes from Tumor regions. The function 'stat_cor' was used to generate the correlation coefficient significance values. To identify EBV high and EBV low infection samples, the tumor transcriptomes from EBV-positive patients were gathered for hierarchical clustering (R function 'hclust' with k = 2) based on the expression of EBV-related transcripts, from which the cluster with higher expression is categorized as EBV high, with the lower categorized as EBV low (Figure S11D). This stratification was then used to compare T cell terminal exhaustion signatures.

Circos plots

To determine the association between the targeted pathway and its corresponding genes, this analysis involved the assessment of each gene's contribution to a specified biological pathway using Spearman's rank correlation coefficient. The samples were first stratified based on EBV condition and cell types, and then Spearman correlation coefficients were computed for these subgroups. A Circos plot, implemented by the 'circlize' R package (version 0.4.15),[103] was subsequently utilized to graphically represent the relationships between gene expression and pathway activity.

CosMx data analysis

All CosMx analyses were performed using the ’seurat’ package. Briefly, data was preprocessed to filter and normalize the expression matrix. Subsequently, the data underwent dimensionality reduction using Principal Component Analysis (PCA), followed by clustering and uniform manifold approximation and projection (UMAP) to identify distinct cellular populations. Manually curated annotation was performed by differential expression analyses starting from the obtained clusters followed by annotation according to the cell type markers specific to each cluster. In particular, HRS cells were annotated on the basis of their characteristic high expression of CD274 (PD-L1), combined with concomitantly low expression of the myeloid marker CD68, allowing discrimination of HRS cells from macrophage populations that also express CD274.[28] Tumor annotations were visually verified by expert pathologists.

Sample stratification based on EBV genes

Samples were stratified according to EBV status as defined by clinical *EBER* stain results. EBV-positive samples were further stratified into LMP1-high and LMP1-low by taking into account both median distribution of pseudobulk FOV LMP1 expression and LMP1 quantile single cell expression in HRS cells.

Distance analysis

The imcRtools package (v1.8.0) was used to calculate the minimum distance of each annotated cell type to tumor cells. This spatial metric served as a proxy for cellular proximity within the heterogeneous tumor microenvironment. To explore these interactions, the GSVA package was used to quantify exhaustion signatures (*CTLA4*, *EOMES*, *HAVCR2*, *LAG3*, *PDCD1*, *TIGIT*, *TOX*, *PRF1*) of $CD4^+$ and $CD8^+$ T cells, allowing to correlate spatial positioning with functional immune cell states.

CODEX-PLA cell level analysis

Assignment of PLA spots

We employed a deep learning model, Piscis,[104] to detect PLA spots. To enhance detection accuracy, we refined the pixel range of the images to compensate for potential variations in spot intensity and background noise. After detection, we assigned each PLA spot to specific pairs of interacting cells based on a set of predefined spatial criteria. Each spot was evaluated within a 10 micron radius to identify neighboring cells. The specific assignment strategy is as follows: Any spot must be adjacent to at least one CD8 T cell as well as one non-CD8 T cell, If this condition was not met, the spot is excluded from further analysis, and if there are multiple CD8 T cells and non-CD8 T cells, we calculated the closest cell based on the Euclidean distance between the spot coordinate and the cell center coordinate. In general, interaction pairs were determined by associating each spot with its nearest CD8 T cell and non-CD8 T cell.

Cell network graph

The (*x*,*y*) coordinate of each cell centroid, derived based on the cell segmentation mask, was used for Delaunay triangulation (scipy 1.15.2). This served as a basis of building a cell network graph (networkx 3.4.2) using the simplices, where two nodes were connected if their Euclidean distance was less than or equal to 10 microns. The adjacent nodes of HRS cells were identified thereafter.

Stratification into LMP1 high and LMP1 low samples

As there was heterogeneity of LMP1 tissue expression across EBV-positive cHL samples (Figure S15B), we visually separated them into samples with high or low abundance of LMP1-expressing HRS cells. This information is in Table S2.

Data visualization

Single channel and multi-color images were assembled with ImageJ. Visualizations of the analysis results were either produced using Excel, or R packages ‘ggplot2’ and ‘complexheatmap’.[105]

Cell Reports Medicine, Volume *7*

Supplemental information

Epstein-Barr virus orchestrates spatial reorganization and immunomodulation in the classic Hodgkin lymphoma tumor microenvironment

Yao Yu Yeo, Huaying Qiu, Yunhao Bai, Bokai Zhu, Yuzhou Chang, Fabio Iannelli, Stephanie Pei Tung Yiu, Jason Yeung, Hendrik A. Michel, Yuchen Wang, Yang Wang, Wenrui Wu, Kyle Wright, Muhammad Shaban, Sam Sadigh, Dingani Nkosi, Vignesh Shanmugam, Philip Rock, Precious Cramer, Julia Paczkowska, Pierre Stephan, Guanrui Liao, Amy Y. Huang, Hongbo Wang, Han Chen, Leonie Frauenfeld, Louisa Kaufmann, Stefano Pileri, Bidisha Mitra, Benjamin E. Gewurz, Bo Zhao, Garry P. Nolan, Baochun Zhang, Alex K. Shalek, Michael Angelo, Christian M. Schürch, Faisal Mahmood, Roberto Chiarle, Qin Ma, W. Richard Burack, Margaret A. Shipp, Scott J. Rodig, and Sizun Jiang

Supplementary Figures

MIBI staining: Antibody Validation

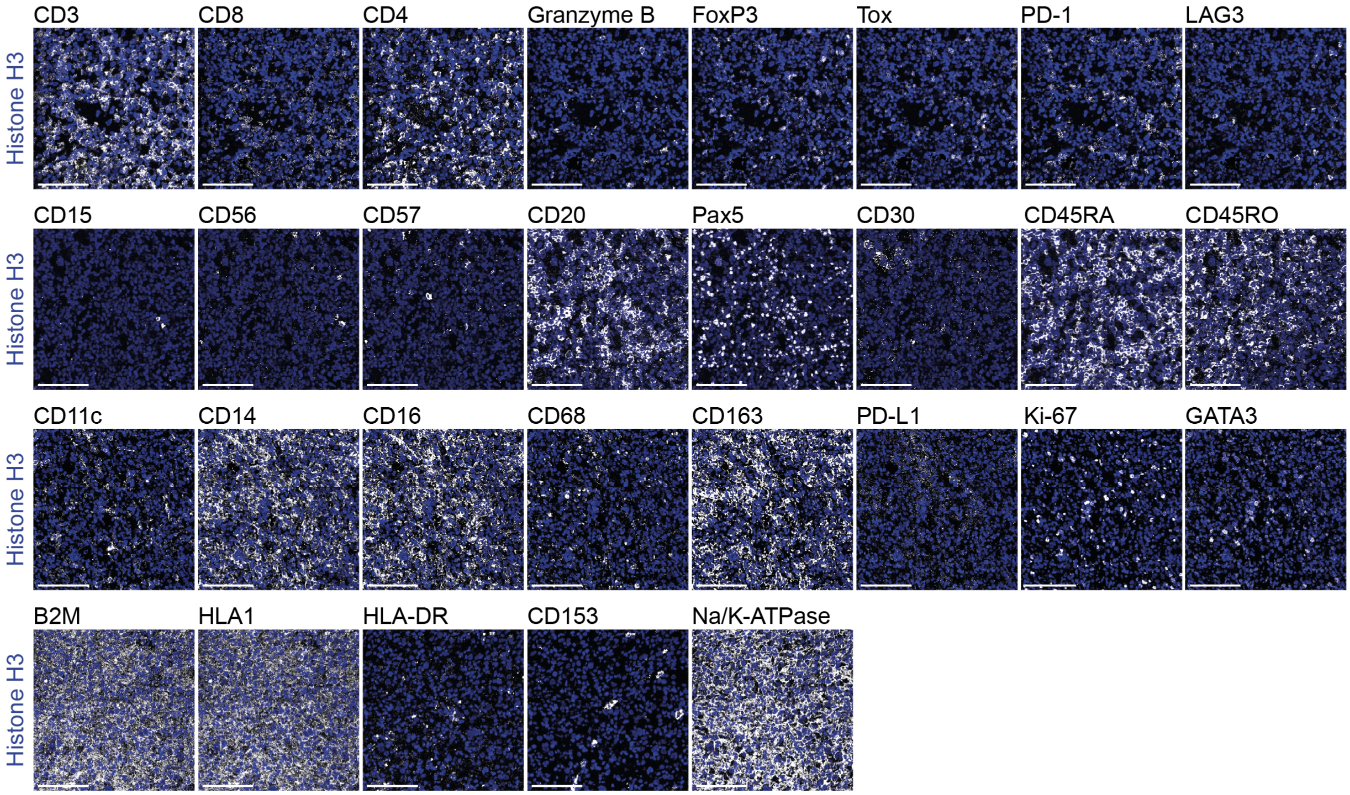

Figure S1, related to Figure 1. Validation of MIBI antibody staining specificity. Representative MIBI images across cHL tissue sections, showing 29 antibody markers (white) overlaid with the cell nucleus antibody marker Histone H3 (blue). Scale bar: 100 µm.

Supp. Fig. 2

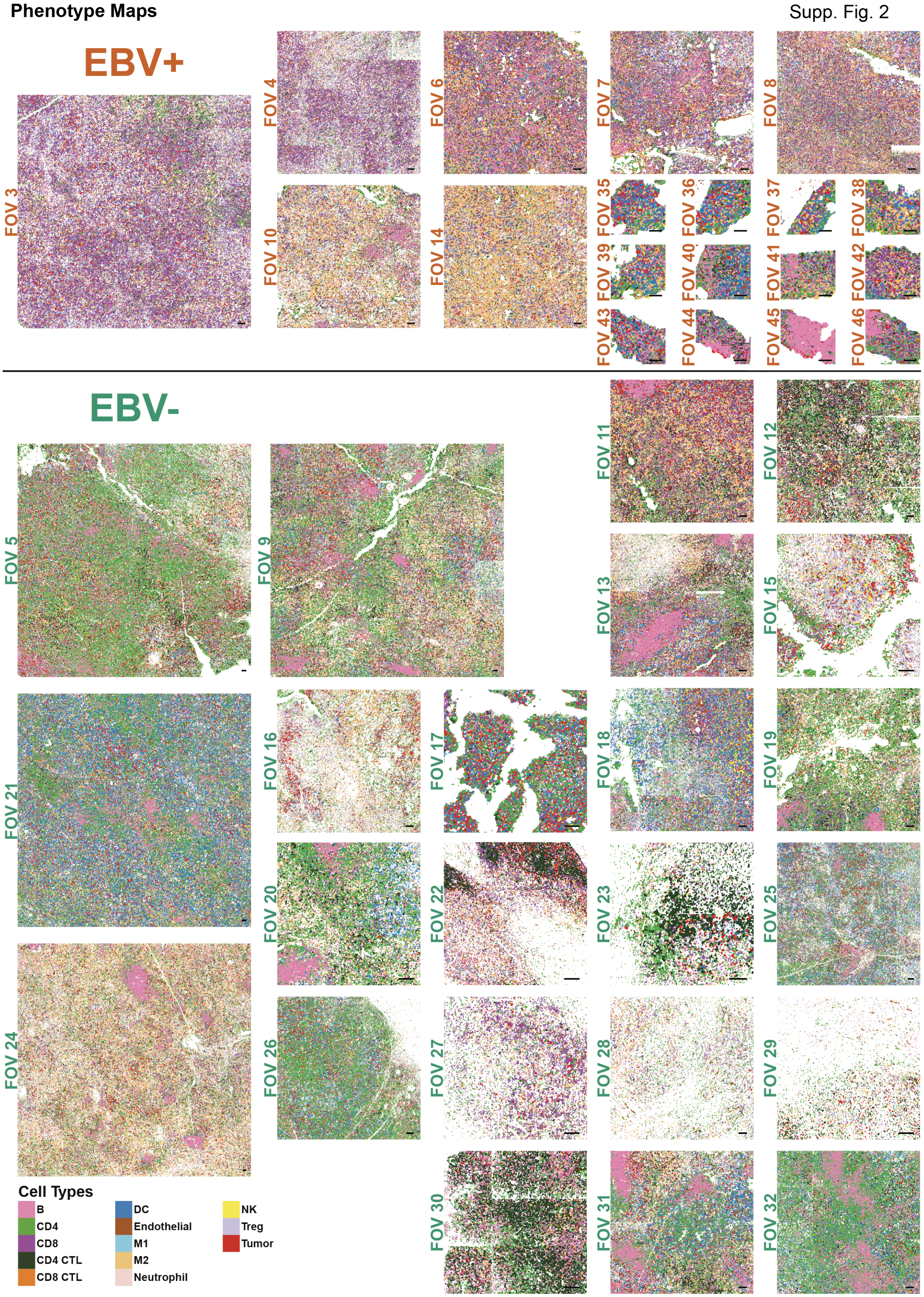

Figure S2, related to Figure 2. Validation of cell phenotyping from MIBI images. Phenotype maps of all the MIBI-acquired FOVs across cHL tissue sections, generated through iterative clustering and annotation based on single-cell MIBI features. Scale bar: 100 µm.

Supp. Fig. 3

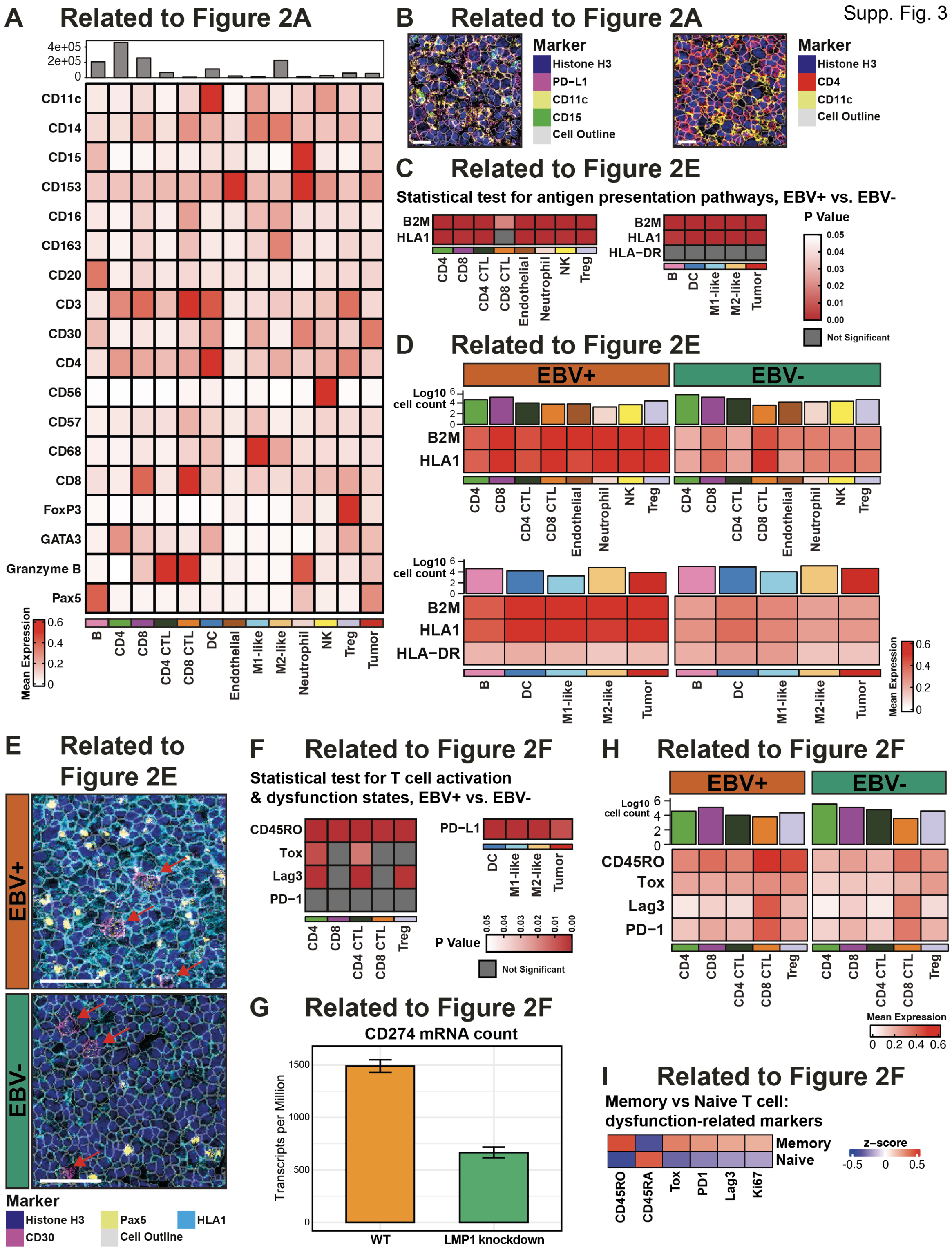

Figure S3, related to Figure 2. Validation and quantification of marker expression on a cell type level. (A) Relative mean expression levels of phenotypic markers for the annotated cell phenotypes in the EBV-positive and EBV-negative cHL MIBI dataset. **(B)** Representative MIBI images showing neutrophils (CD15) close to immune constituents that have express PD-L1, such as dendritic cells (CD11c) (left), and dendritic cells (CD11c) in close contact with CD4 T cells (CD4) (right). Cell outlines are shown to indicate segmented cell boundaries. Scale bar: 20 µm. **(C)** and **(F)** Test results for **Figs. 2E** & **2F**, respectively. P values were generated from one-sided Wilcoxon rank sum tests, with the alternative hypothesis that the distribution of a given marker for the EBV+ population is shifted to the right of the distribution for the EBV- population. The test results were adjusted for multiple comparisons using the Benjamini-Hochberg method with a targeted FDR at 0.05. Unadjusted p-value and Benjamini-Hochberg corrected test results are in **Supp Table 4**. **(D)** and **(H)** Relative mean expression level heatmaps, a re-representation of relative z-score expression level heatmaps in **Figs. 2E** & **2F**. **(E)** Representative MIBI images showing MHC Class I (HLA1) expression differences between the EBV-positive and EBV-negative cHL TME, with additional markers for B cells (Pax5^{hi}) and HRS cells (CD30, Pax5^{lo}) shown. Red arrows point to HRS cells. Cell outlines are shown to indicate segmented cell boundaries. Scale bar: 50 µm. **(G)** Relative expression of *CD274* that encodes for PD-L1 upon knockdown of LMP1 in the EBV-positive GM12878 cell line (n=3 each). The data used to generate this barplot is in **Supp Table 9**. **(I)** Expression heatmap of immune exhaustion markers on T cells stratified into memory and naive populations based on the relative expression of CD45RO and CD45RA. The data used to generate this heatmap is in **Supp Table 10**.

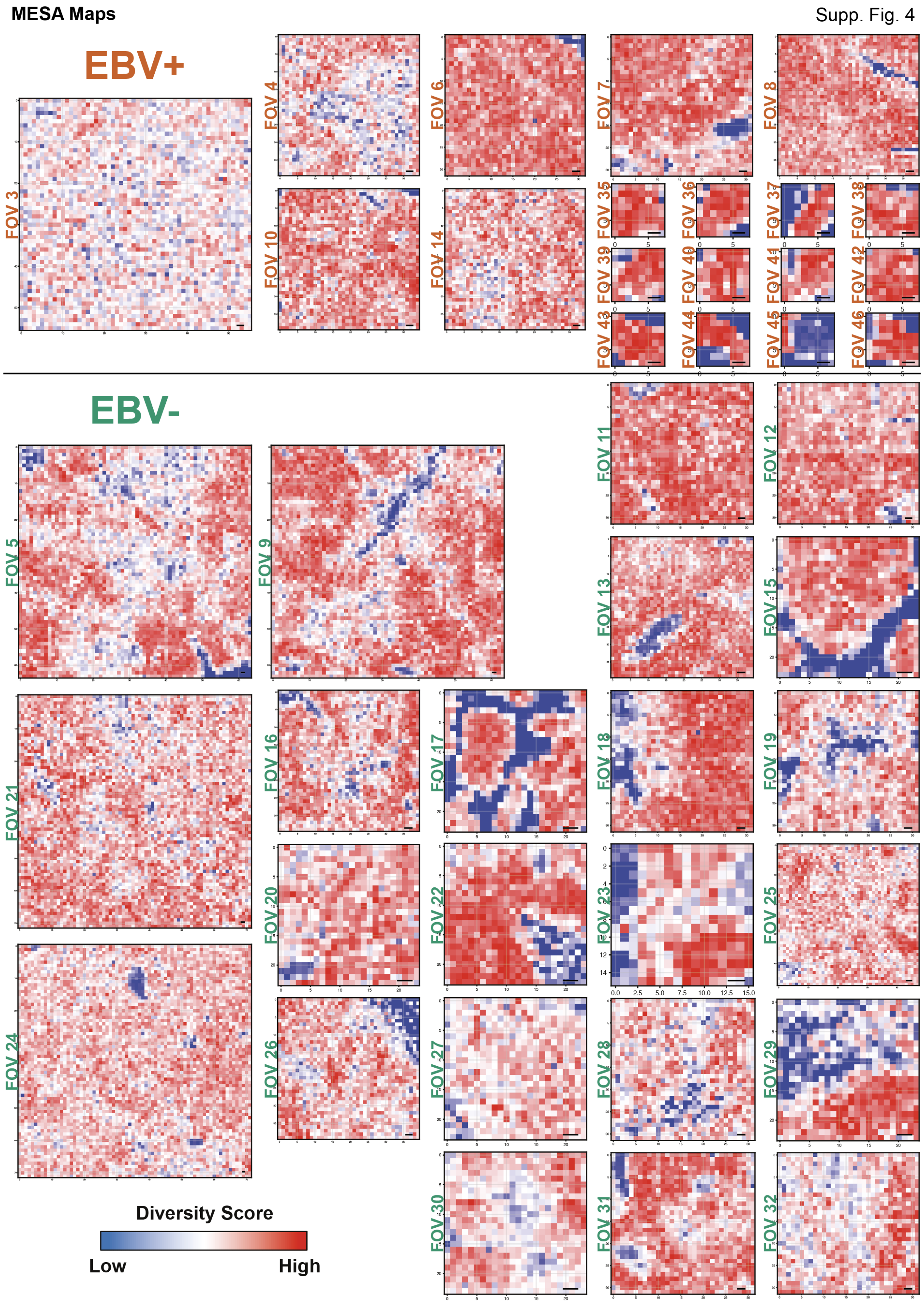

Figure S4, related to Figure 3. Validation of spatial diversity hotpot identification from the annotated cell types. Spatial diversity maps of all MIBI-acquired FOVs across cHL tissue sections, generated through MESA (1) based on annotated cell phenotypes (see **Supp Fig. S2**). Scale bar: 100 µm.

Cell Neighborhood Maps

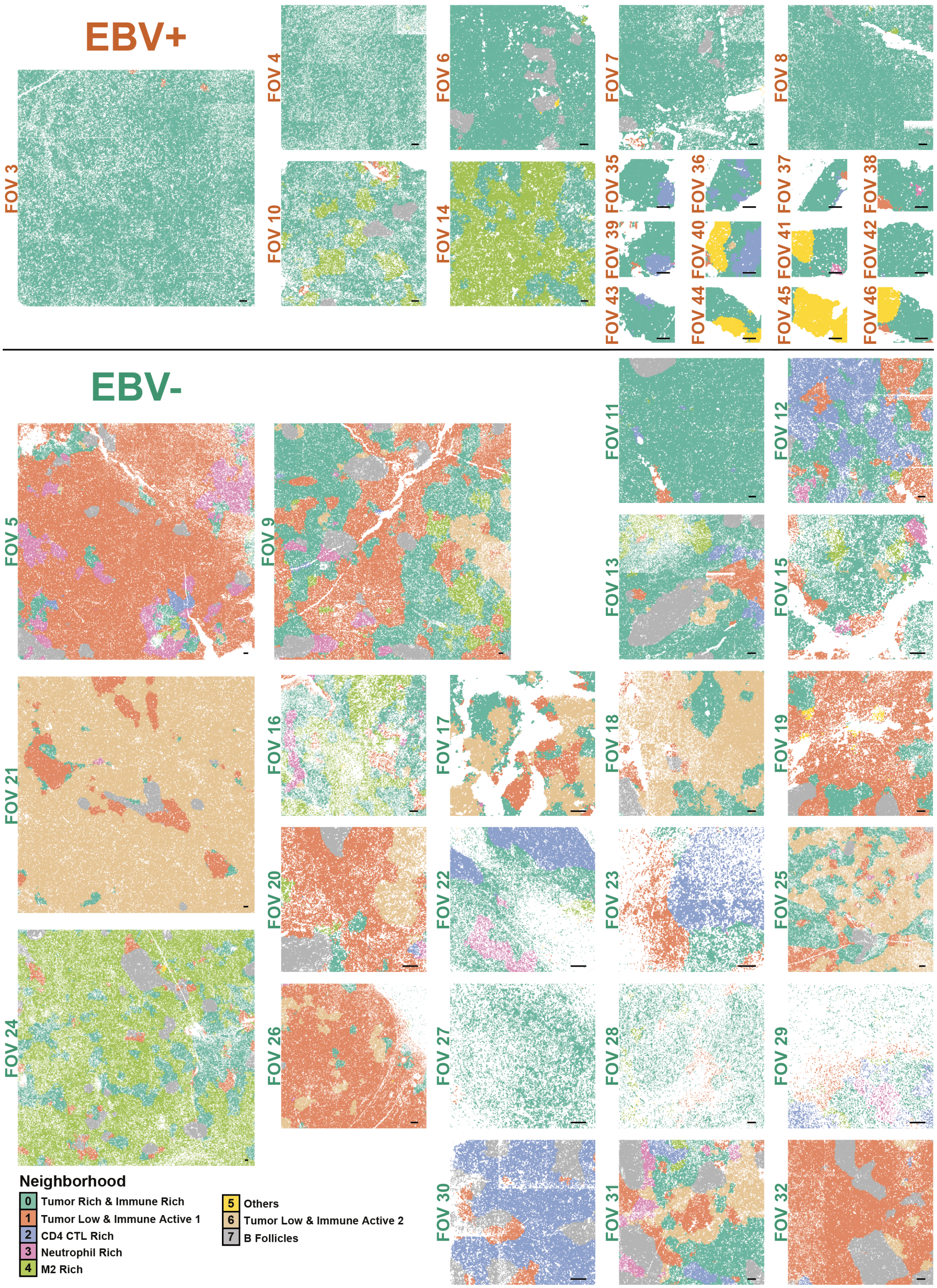

Figure S5, related to Figure 3. Validation of cell neighborhood identification from annotated cell types. Cell neighborhood maps of all MIBI-acquired FOVs across cHL tissue sections, generated through spatial LDA (2) based on annotated cell phenotypes (see **Supp Fig. S2**). Scale bar: 100 µm.

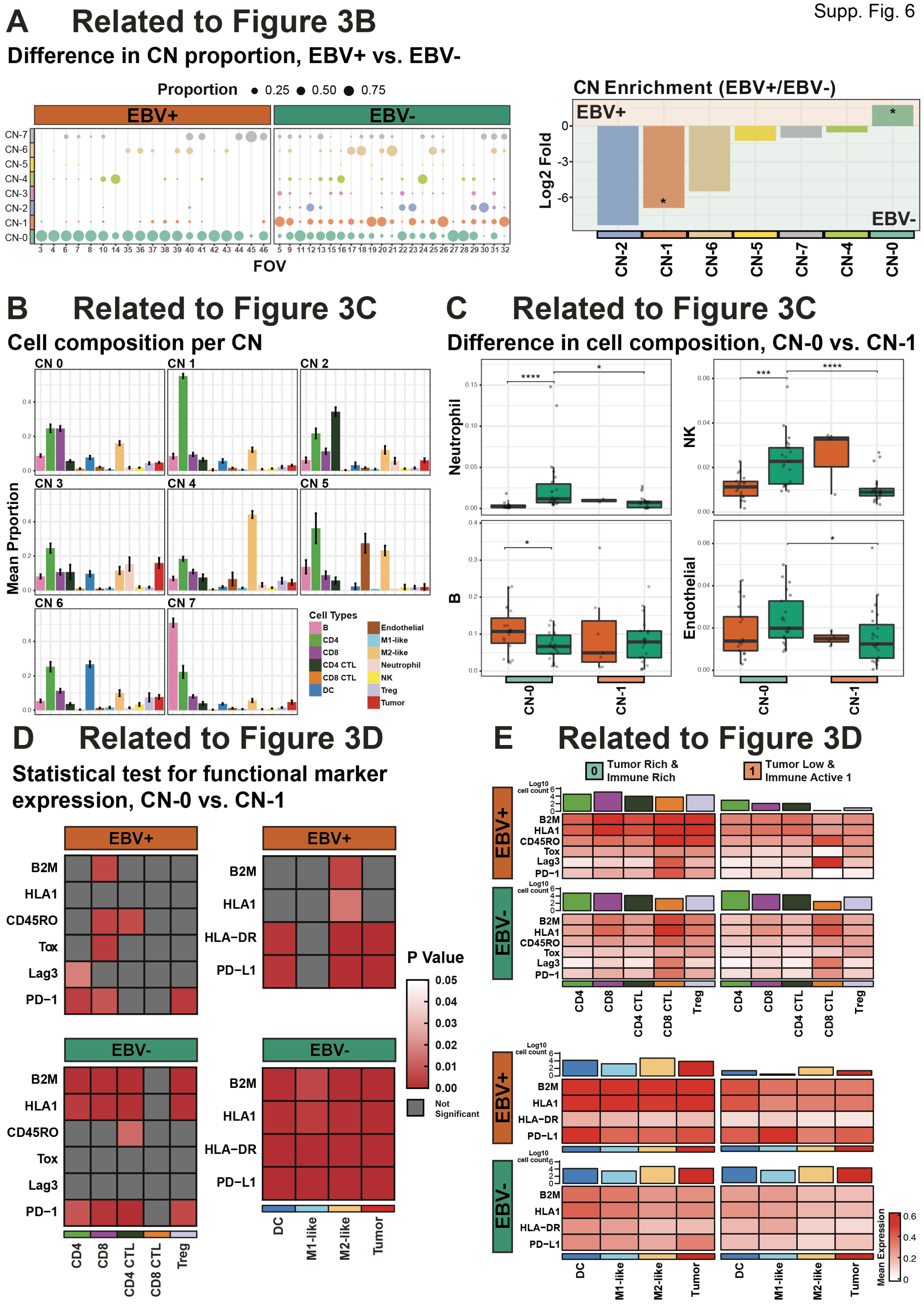

Figure S6, related to Figure 3. Statistical test results for comparison of cell proportions and functional marker expression on a cell neighborhood level. **(A)** Left: Relative proportion of cells assigned to each CN across EBV-positive and EBV-negative cHL FOVs. Right: Log2 fold enrichment plot of cell proportions assigned to each CN between EBV-positive and EBV-negative cHL FOVs. Note that CN3 is not shown due to its absence from the EBV+ cHL TME. Significance stars (* $p \leq 0.05$) are only shown for statistically significant comparisons ($p \leq 0.05$). Two-sided Wilcoxon rank sum tests were conducted for all cell type proportions within each CN, with alternative hypotheses that the proportions of the given cell type in each CN present in the two strata were not equal. Test results were adjusted for multiple comparisons using the Benjamini-Hochberg method with a targeted false discovery rate (FDR) at 0.05. **(B)** Abundance of each cell type within each CN (mean ± 1 s.e.). **(C)** Relative proportion of other cell types within CN-0 and CN-1, stratified by EBV status. Significance stars are only shown for statistically significant comparisons ($p \leq 0.05$). **(D)** Test results for **Fig. 3E**. P values were generated from two-sided Wilcoxon rank sum tests, with the alternative hypothesis that within the EBV+ or EBV- population respectively, the distribution of a given marker is different across CN-0 and CN-1. The test results were adjusted for multiple comparisons using the Benjamini-Hochberg method with a targeted FDR at 0.05. Unadjusted p-value and Benjamini-Hochberg corrected test results are in **Supp Table 4**. **(E)** Relative mean expression level heatmap, a re-representation of relative z-score expression level heatmap in **Fig. 3E**.

Tumor Score Maps

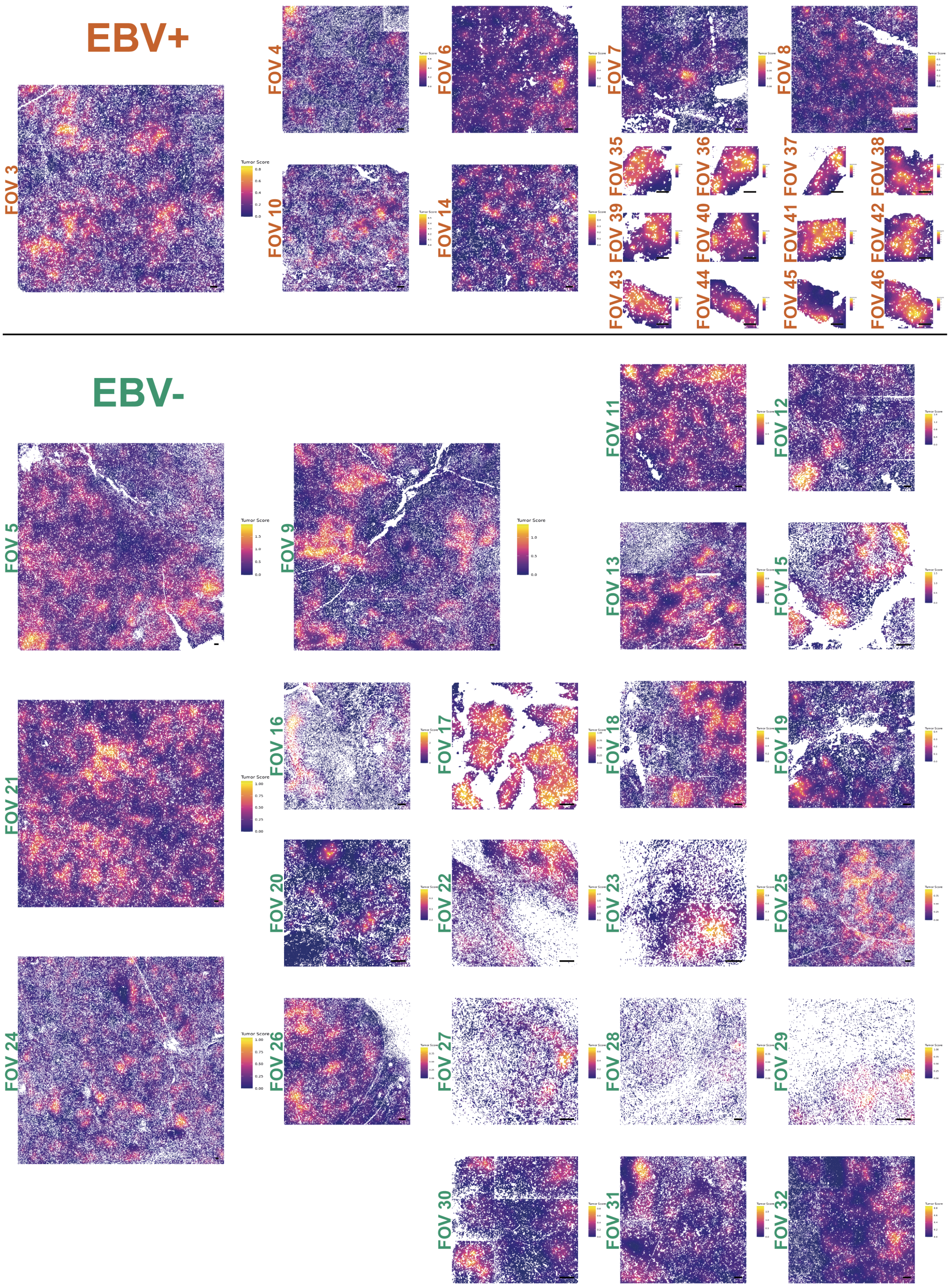

Figure S7, related to Figure 4. Validation of tumor score metric. Tumor score maps of all MIBI-acquired FOVs across cHL tissue sections, generated based on the spatial proximity to tumor cells (see **Materials & Methods**). HRS cells are not considered in the tumor score metric and do not have a color assigned. Scale bar: 100 µm.

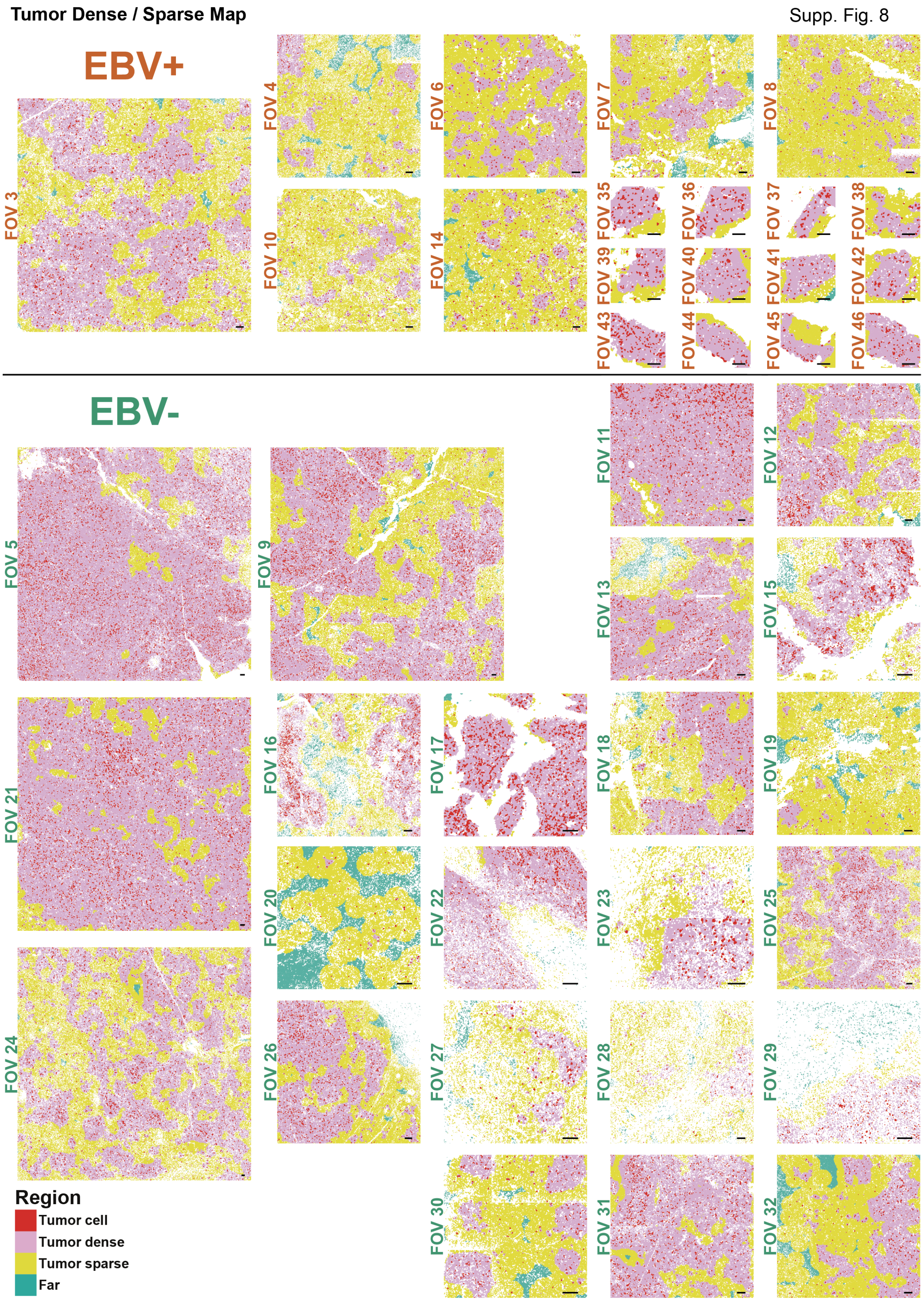

Figure S8, related to Figure 4. Validation of tumor dense/sparse region stratification. Tumor dense and tumor sparse maps of all 34 MIBI-acquired FOVs across cHL tissue sections, generated based on the tumor score metric of each non-tumor cell (see **Materials & Methods**). Scale bar: 100 µm.

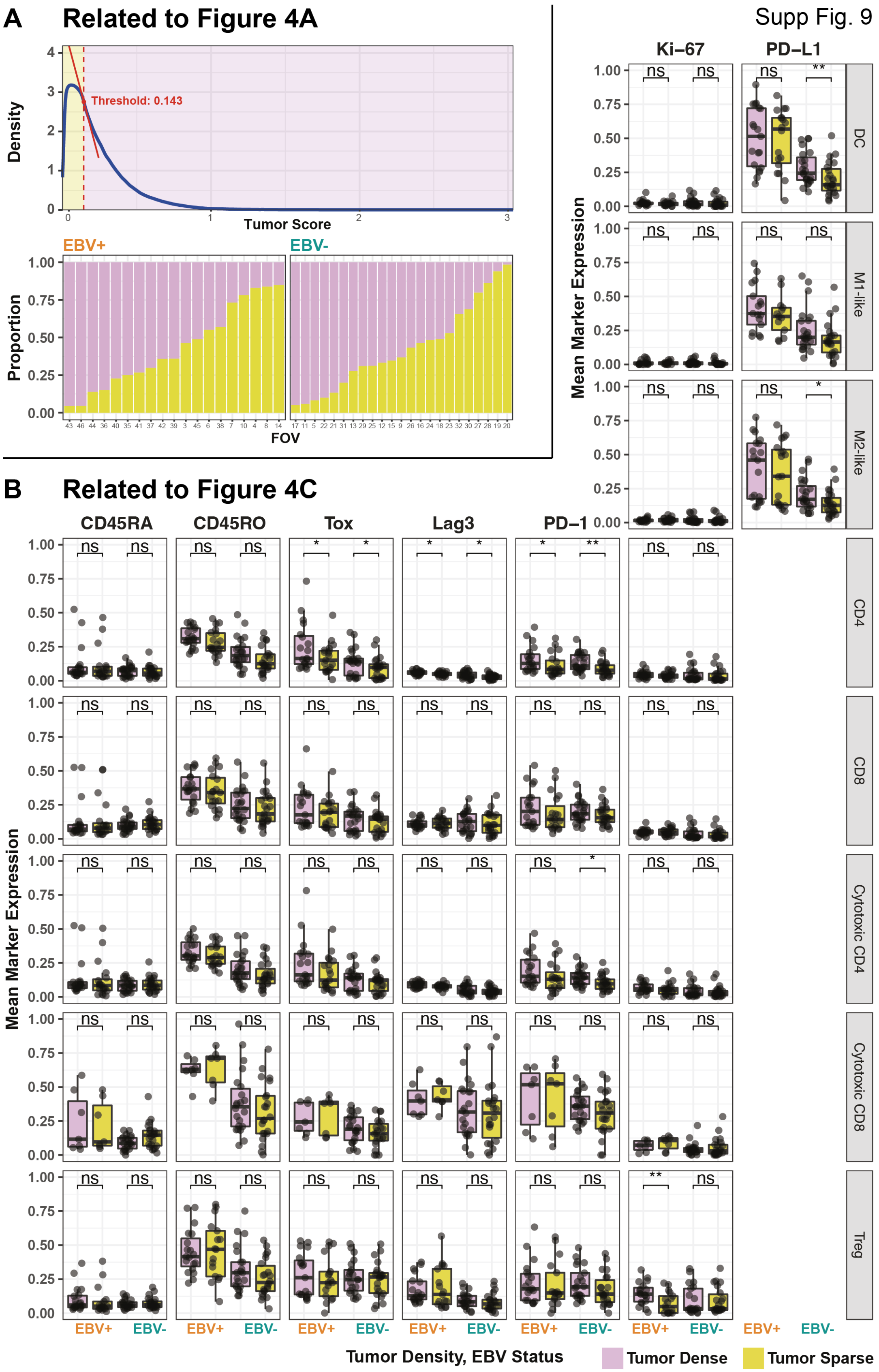

Figure S9, related to Figure 4. Tissue stratification into tumor dense/sparse regions and comparison of the corresponding functional marker expression. (A) Top: Cutoff point for stratifying cells into tumor dense/sparse regions. Bottom: Barplot showing the proportion of cells in each FOV that are assigned to tumor dense/sparse regions, stratified by EBV status. **(B)** Relative expression of markers that comprise the exhaustion score metric. For each tumor dense and tumor sparse pair, a one-sided T test was performed, with the alternative hypothesis that the difference between the mean exhaustion score of the tumor dense region in an FOV and that of the tumor sparse region is greater than 0 (* $p \leq 0.05$, ** $p \leq 0.01$, *** $p \leq 0.001$, **** $p \leq 0.0001$). The test results were adjusted for multiple comparisons using the Benjamini-Hochberg method with a targeted false discovery rate (FDR) at 0.05. Unadjusted p-value and Benjamini-Hochberg corrected test results are in **Supp Table 4**.

Supp Fig. 10

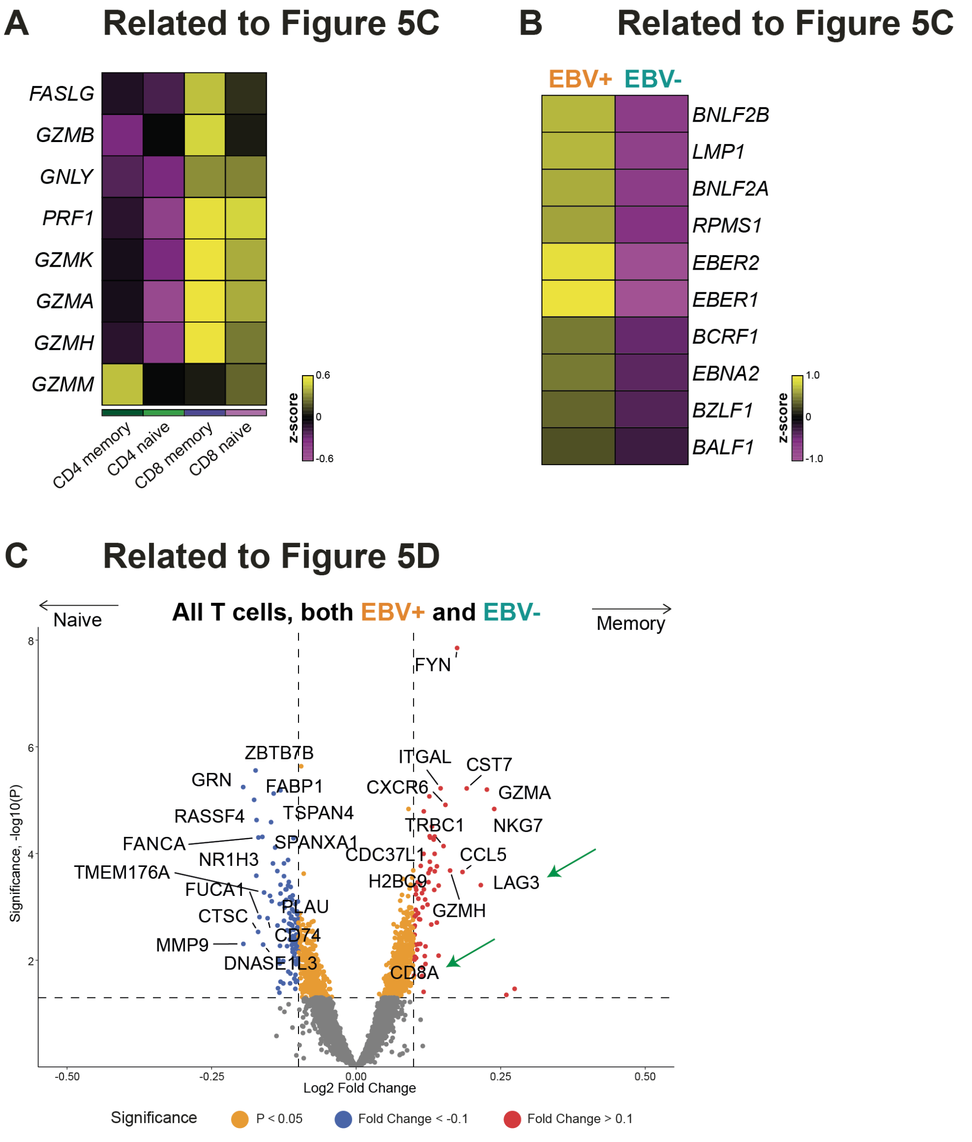

Figure S10, related to Figure 5. Comparison EBV-linked transcriptional differences. (A) Expression heatmap of cytotoxic transcripts associated with each annotated cell region. **(B)** Expression heatmap of EBV genes in EBV-positive and EBV-negative tumor regions. **(C)** Volcano plot of memory (CD45RO+) vs. naive (CD45RO-) T cells, with some of the most differentially expressed genes shown. *CD8A* and *LAG3* are indicated by the green arrows. The differential gene expression data for each capture region are in **Supp Table 11**.

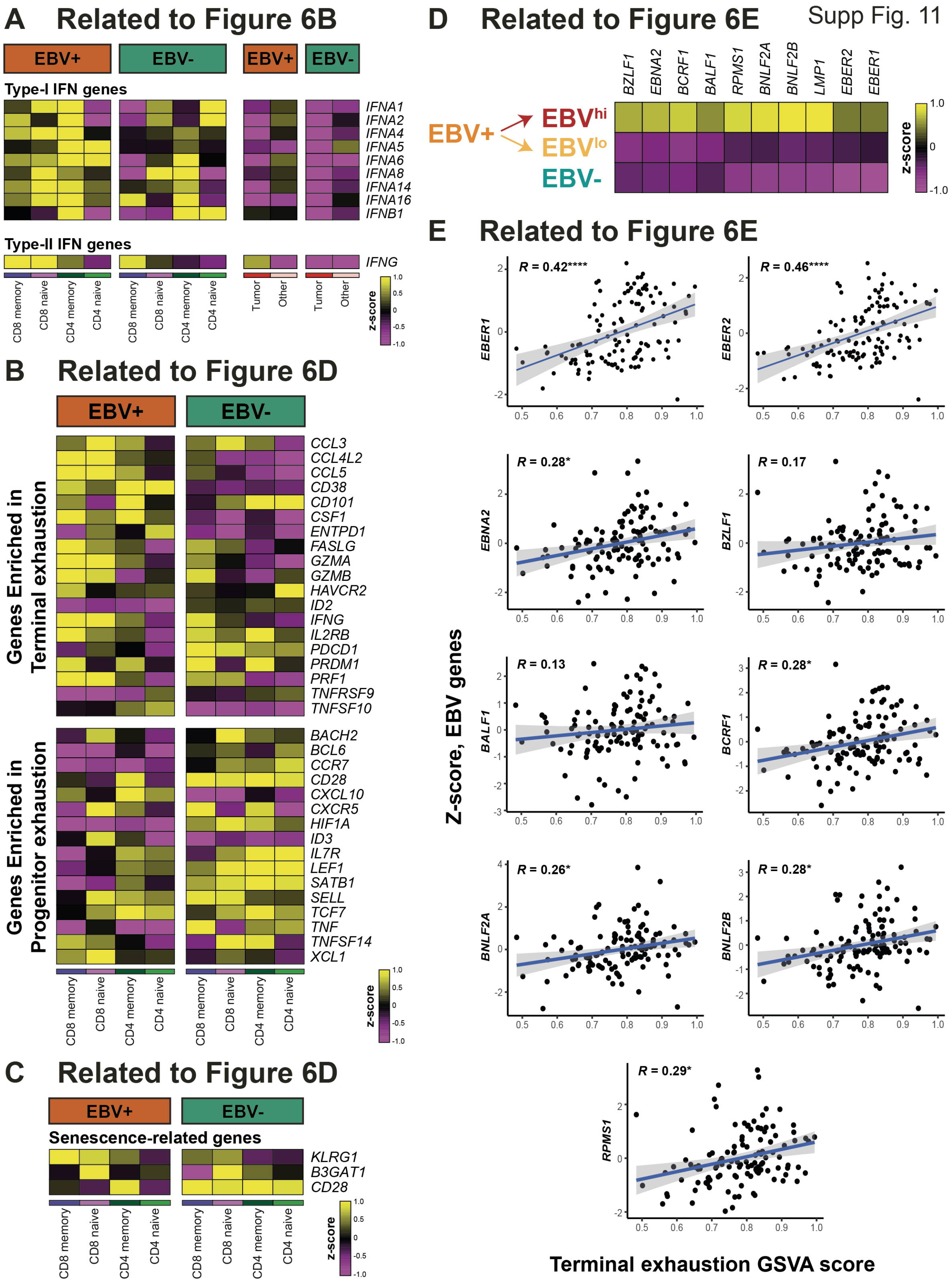

Figure S11, related to Figure 6. Expressions of genes used to score specific gene sets. **(A)** Expression heatmap of interferons used to score "Type-I IFN genes" and "Type-II IFN genes" pathways in **Fig. 6B**. This list does not include all known interferons because it only contains probes available in the GeoMx probe set. **(B)** Expression heatmap of key differentially expressed genes between terminal and progenitor T-cell exhaustion states (see Fig. 2D in (3)). **(C)** Expression heatmap of genes and pathways associated with T-cell senescence (4). **(D)** Spearman correlation of each EBV transcript with T-cell terminal exhaustion GSVA score. **(E)** Expression of each EBV transcript upon stratifying EBV-positive samples into EBV-high and EBV-low categories.

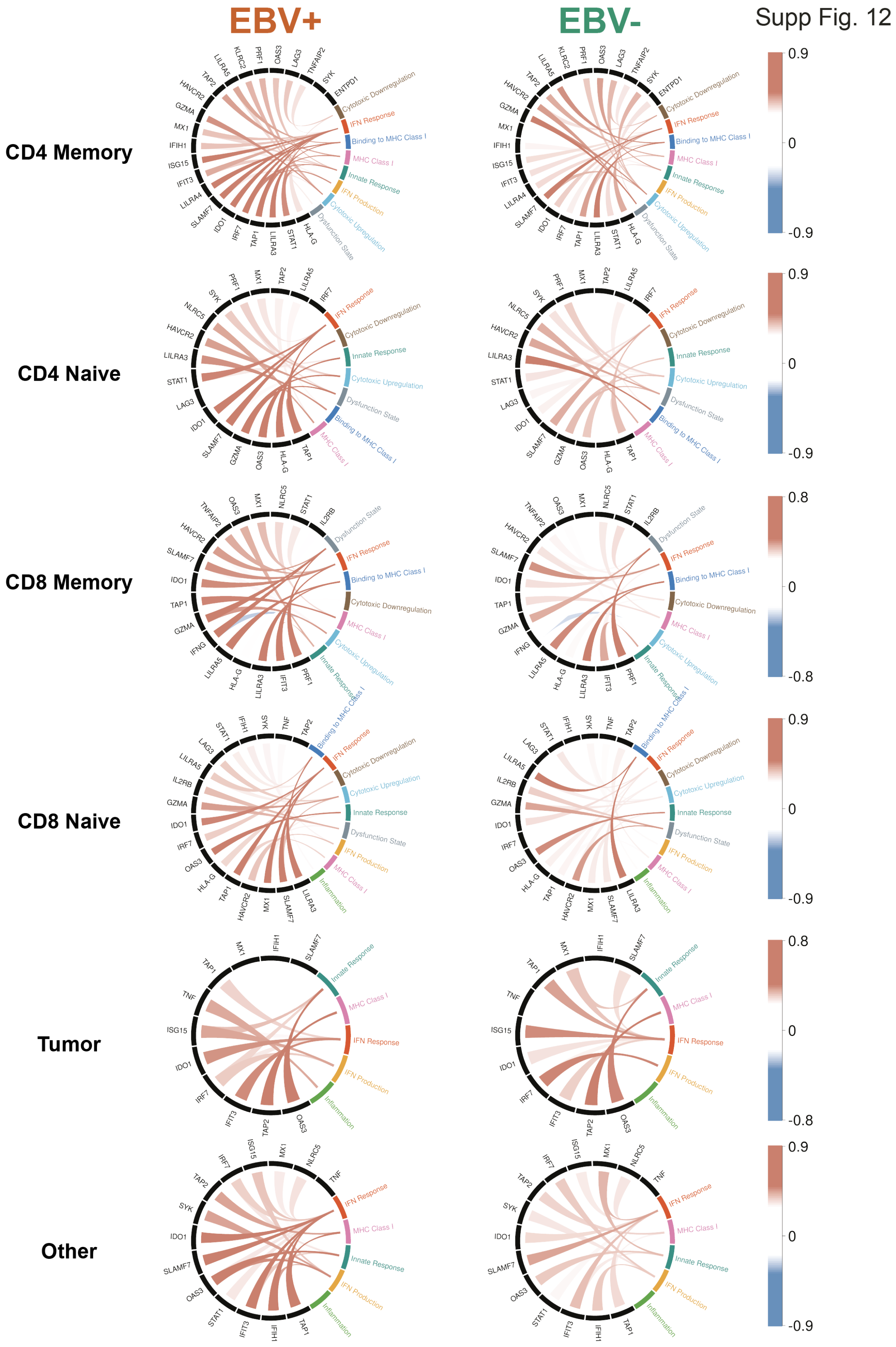

Figure S12, related to Figure 6. Comparison of representative gene-pathway correlations for each cell population, stratified by EBV status. Circos plot for each cell population profiled with the GeoMx.

CODEX PLA staining: Antibody Validation

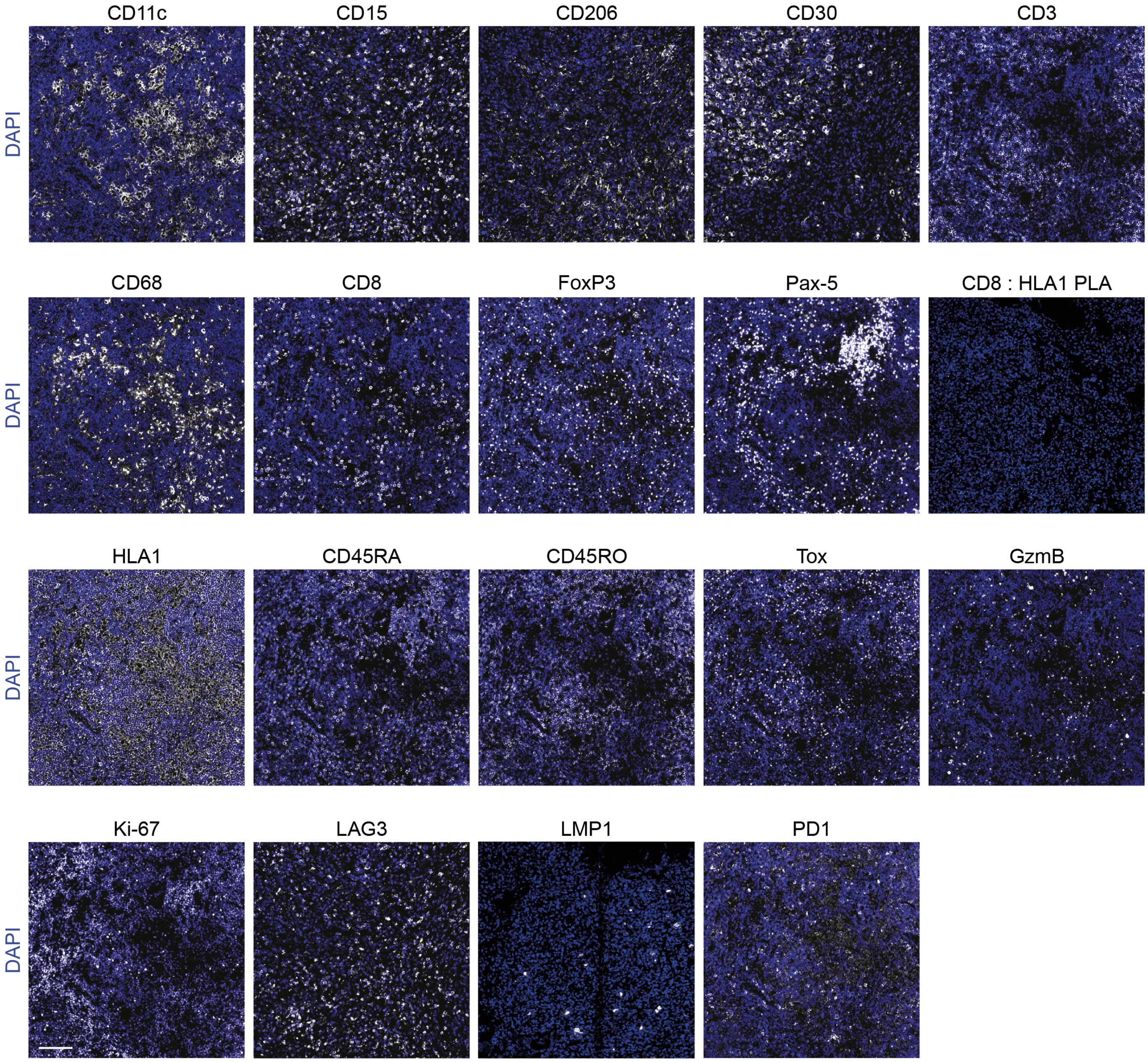

Figure S13, related to Figure 7. Validation of CODEX staining specificity. Representative CODEX images across cHL tissue sections, showing each (white) overlaid with the cell nucleus dye DAPI (blue). Scale bar: 100 µm.

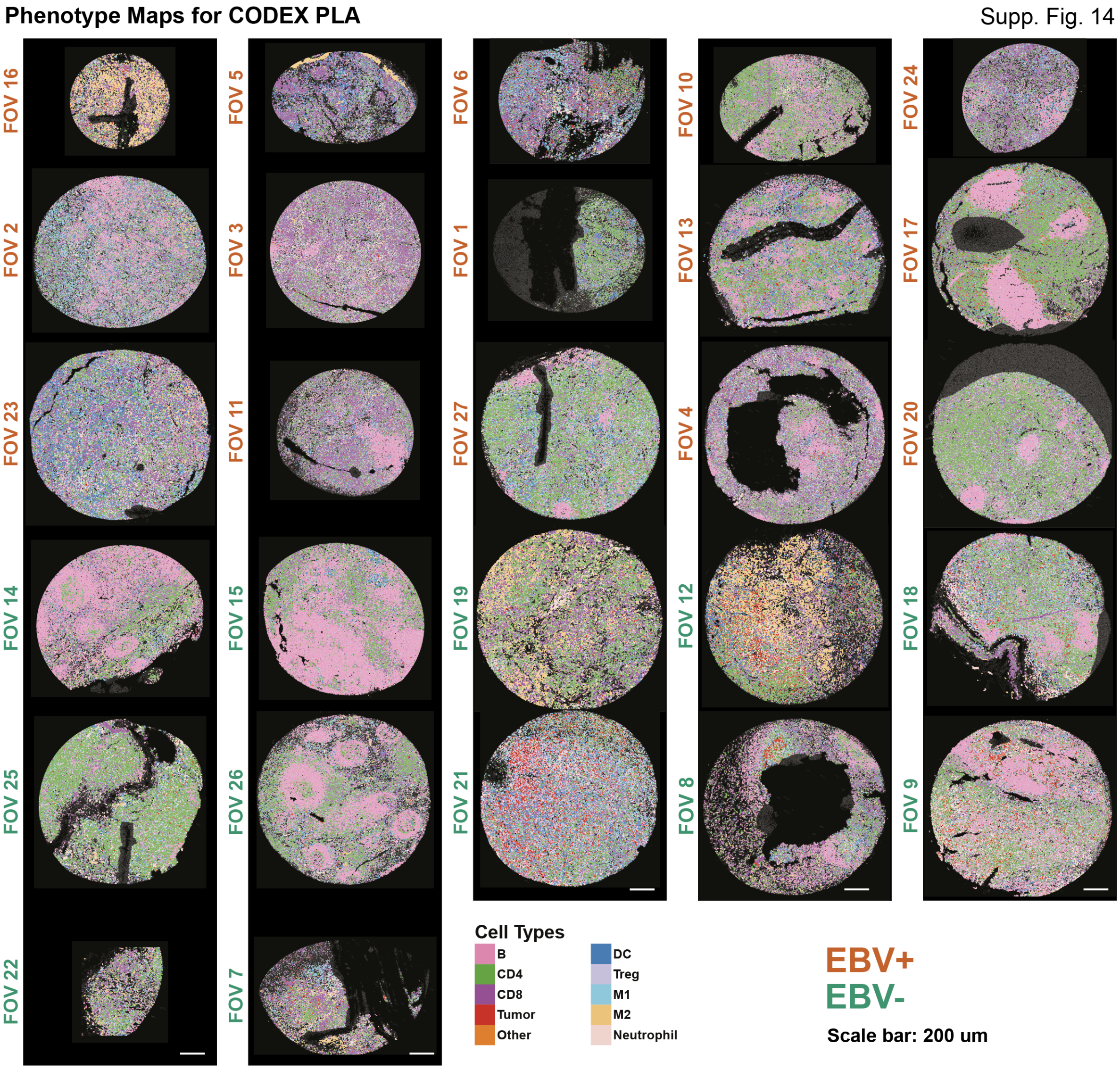

Figure S14, related to Figure 7. Validation of cell phenotyping from CODEX images. Phenotype maps of all the CODEX acquired FOVs across cHL TMA, generated through iterative clustering and annotation based on single-cell CODEX features. Scale bar: 200 µm.

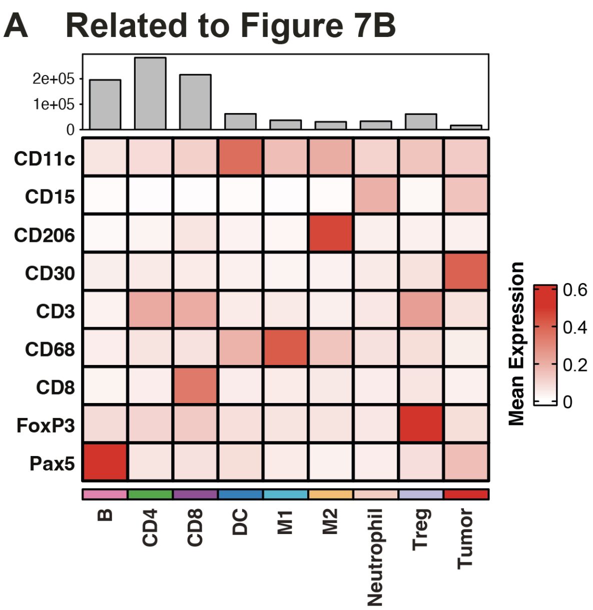

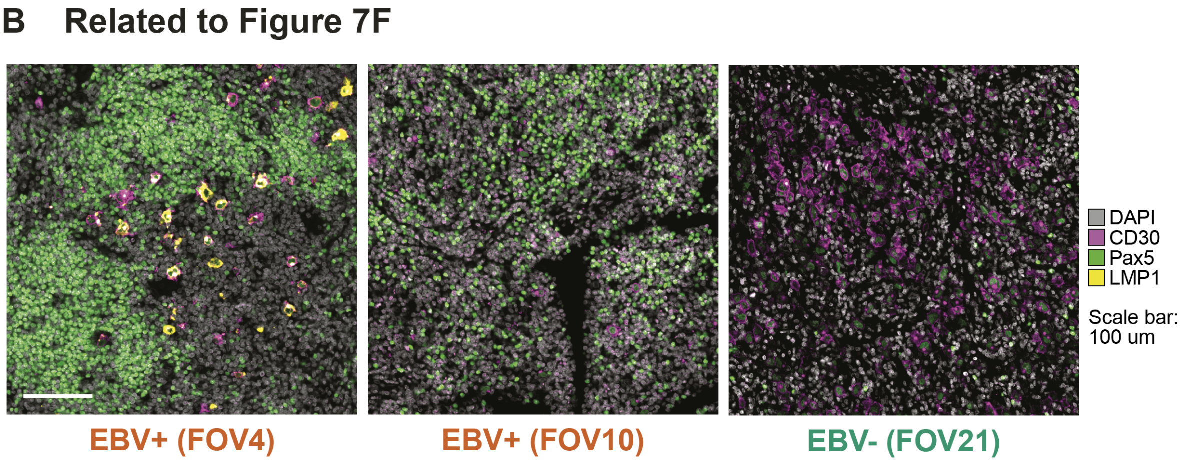

Figure S15, related to Figure 7. Validation and quantification of marker expression. (A) Relative mean expression levels of phenotypic markers for the annotated cell phenotypes across the CODEX-PLA dataset. **(B)** Representative images showing high and low abundance of LMP1 expression in HRS cells within EBV-positive cHL, as well as the lack of LMP1 staining in EBV-negative cHL.

Reference

1. Daisy Yi Ding, Zeyu Tang, Bokai Zhu, Hongyu Ren, Alex K Shalek, Robert Tibshirani, and Garry P Nolan. Quantitative characterization of tissue states using multiomics and ecological spatial analysis. *Nature Genetics*, pages 1–12, 2025.
2. Z. Chen, I. Soifer, H. Hilton, L. Keren, and V. Jojic. Modeling multiplexed images with spatial-lda reveals novel tissue microenvironments. *J Comput Biol*, 27(8):1204–1218, 2020. doi: 10.1089/cmb.2019.0340.
3. BC Miller, DR Sen, RA Abosy, K Bi, YV Virkud, MW LaFleur, KB Yates, A Lako, K Felt, GS Naik, and et al. Subsets of exhausted cd8+ t cells differentially mediate tumor control and respond to checkpoint blockade. *Nat Immunol*, 20(3):326–336, 2019. doi: 10.1038/s41590-019-0312-6.
4. EJ Wherry and M Kurachi. Molecular and cellular insights into t cell exhaustion. *Nat Rev Immunol*, 15(8):486–499, 2015. doi: 10.1038/nri3862.
